# Mass media impact on opinion evolution in biased digital environments: a bounded confidence model

**DOI:** 10.1038/s41598-023-39725-y

**Published:** 2023-09-05

**Authors:** Valentina Pansanella, Alina Sîrbu, Janos Kertesz, Giulio Rossetti

**Affiliations:** 1 Faculty of Science, Scuola Normale Superiore, Pisa, Italy; 2 Department of Computer Science, University of Pisa, Largo Bruno Pontecorvo 3, Pisa, Italy; 3 Department of Network and Data Science, Central European University, Vienna, Austria; 4 Institute of Information Science and Technologies “A. Faedo” (ISTI), National Research Council (CNR), G. Moruzzi 1, Pisa, Italy

**Keywords:** Complex networks, Computational science, Computer science, Human behaviour

## Abstract

People increasingly shape their opinions by accessing and discussing content shared on social networking websites. These platforms contain a mixture of other users’ shared opinions and content from mainstream media sources. While online social networks have fostered information access and diffusion, they also represent optimal environments for the proliferation of polluted information and contents, which are argued to be among the co-causes of polarization/radicalization phenomena. Moreover, recommendation algorithms - intended to enhance platform usage - likely augment such phenomena, generating the so-called *Algorithmic Bias*. In this work, we study the effects of the combination of social influence and mass media influence on the dynamics of opinion evolution in a biased online environment, using a recent bounded confidence opinion dynamics model with algorithmic bias as a baseline and adding the possibility to interact with one or more media outlets, modeled as stubborn agents. We analyzed four different media landscapes and found that an open-minded population is more easily manipulated by external propaganda - moderate or extremist - while remaining undecided in a more balanced information environment. By reinforcing users’ biases, recommender systems appear to help avoid the complete manipulation of the population by external propaganda.

## Introduction

Opinions and beliefs shape individual behavior, which drives human actions, and a society’s collective behavior, influencing politics, public health, and the environment. Changes in public opinion - even the formation of committed minorities - may profoundly affect decision-making and politics: a recent example is the temporary suspension of the Oxford-AstraZeneca vaccine during March 2021^1^, which has cost a slowdown in the vaccination strategy and had direct consequences on public health. Social interactions^2^ are the main ingredient driving the opinion evolution process. According to social influence theory^3^, an interaction between social agents typically reduces the difference between their opinions or, at worst, leaves it unchanged. Besides social influence, opinion formation also depends on the information people collect from external sources (mainly in the form of mass media broadcasts), enhancing awareness of socio-political issues and events^4, 5^. For instance, traditional mass media have been argued to influence individual and public health^6, 7^ on issues ranging from eating disorders^8^, tobacco consumption^9^, and vaccinations^10^. Moreover, news articles, TV news, and political talk shows all play a central role in shaping opinions, especially when it comes to the communication of political information, which is a key process in the political system, arguably holding the power to manipulate how people think about internal and international politics.

However, media coverage often exhibits an internal bias, reflected in the news and commonly referred to as media bias^11^. Factors influencing this bias include ownership or a specific political or ideological stance of the outlet and its target audience^12^. Media choices can also be influenced by their profit-oriented nature, leading to content selection aligned with the audience’s interests that fuels this profit, disregarding issues and problems (and portions of the population, such as minorities) that would guarantee fewer earnings^13^.

As theoretical studies show, reading news or being the target of mass political propaganda^14, 15^ may affect our belief system. External agents (i.e. a government, a company, or a group of terrorists) may be interested in actively shifting the public’s opinion concerning a specific topic. Propaganda can be exploited to try and promote one opinion over the others^16^, to achieve a certain value for the consensus opinion^17^, or even to prevent people from reaching more extreme opinions^18^. However, when agents have different opinions, a single aggressive media may, in reality, produces an undesired result^19^: an antagonist cluster at the opposite extreme of the opinion spectrum.

Besides the information social agents can access, and how this information is presented to them, a series of internal mechanisms play an important role in shaping opinions and beliefs. The way people process information is, in fact, far from being perfectly rational and is highly influenced by psychological factors and cognitive biases^20^. Psychological studies^21, 22^ have observed that people, both online and offline, feel discomfort when encountering opinions and ideas that contradict their existing beliefs, i.e. experience cognitive dissonance^23^. Such cognitive biases have often been studied through models of bounded confidence^24^, i.e. the tendency to ignore beliefs that are too far from our current ones, or mimicking the backfire effects^25^, i.e. the tendency to reject countering evidence and to strengthen the support to the current belief. When considering cognitive biases, extremist propaganda may become efficient when the message is promoted with a certain frequency^26^. When the propaganda is made on more moderate stances or when the population is more open-minded, the population conforms to the propaganda if the message is delivered frequently enough. When the media landscape is heterogeneous^27^, media outlets can employ different strategies to maximize their audience. For instance, on some issues of general interest, each media outlet tries to imitate successful behaviors (e.g. promoting closer opinions to the most followed media). On other more ideologically charged issues, media outlets may compete (i.e. disagreeing with the other media), promoting thus opinion fragmentation in the population. The presence of repulsive behaviors^28^ suggests that propaganda can drive the population to form a consensus around an external message, regardless of whether the message is extreme or moderate: as a result of wanting to be apart, agents end up together sharing the same opinion.

While such a dynamic has always existed, how people retrieve information has profoundly changed in the last twenty years. Television remains the most common media source among Europeans^29^, but the use of the Internet and online social networks (OSNs) is steadily rising alongside the decline of the readership of newspapers. However, OSNs are also environments where individuals express their opinions, discuss, and share content from other sources. These environments are ruled by algorithms that filter and personalize each user’s experience accordingly to their and their friends’ past behavior. This is intended to maximize users’ engagement and enhance platform usage, however it is theorized that filtering algorithms and recommender systems are likely to create an algorithmic bias^30^. By showing people only narratives aligned with their own existing beliefs, a positive feedback loop is obtained, reducing the amount of diversity in the user experience, contributing to the creation and maintenance of echo chambers^31^ and filter bubbles^32–34^. Although personalization is essential in information-rich environments (to allow people to find what they are looking for and increase user engagement), there is great concern about the negative consequences of algorithmic filtering. Therefore, understanding how mass media information impacts public opinion and how cognitive and algorithmic biases play a role in social influence mechanisms is essential to enrich our understanding of human behavior and also to define mitigation strategies to avoid unintended consequences.

In this paper, we approach such a goal through the lens of opinion dynamics models^35–42^, a field of study born within the statistical physics area which is now mainly studied through the lens of computational social sciences. Indeed, the possible effects of mass media have been widely investigated through such models^14–18, 18, 26, 27, 43–47^. However, to the best of ourknowledge, none of these works combine the role of online platforms and algorithmic biases with different possible media landscapes. The present work aims to analyze the effects of different mass media landscapes - ranging from extremist propaganda to a more balanced opinion diet - in a synthetic environment, simulating a general OSN where agents can interact with each other, but interactions are always mediated by a recommender system, selecting content aligned with agent beliefs. To investigate the role of mass media in shaping public opinion, we extended the Algorithmic Bias model^30^ (which, in turn, extends the Deffuant-Weisbuch one^24^), adding the possibility to specify a number of external mass media agents, defining the opinions they promote, and the frequency of agent-media interactions. We conducted numerical simulations to examine this extended model and analyzed the outcomes within the context of mean-field scenarios. Furthermore, we present a case study on a real-world network, illustrating how this model effectively captures a behavior that the baseline model fails to capture.

## Results

The present work aims to extend the Algorithmic Bias model^30^ to understand how interacting with mass media in a biased environment (i.e. ruled by recommender systems and filtering algorithms) influence the outcome of the opinion evolution. In our simulations, we consider 100 agents with continuous opinions in the interval [0, 1], which can model opinions on any issue, with values 0 and 1 representing the most extreme opinions. The agents are allowed to interact with each other at discrete time intervals and with a fixed number of *M* stubborn agents, representing traditional media outlets that promote a fixed opinion over the whole time period. To represent this environment realistically, interactions (agent-to-agent and media-to-agent) are subject to cognitive and algorithmic biases. The stronger the algorithmic bias, $\gamma$, the higher the probability of interacting with similar agents and the lower the probability of interacting with different ones. Cognitive bias - specifically bounded confidence - limits interaction to an agent’s opinion neighborhood: two agents influence each other (according to social influence theory, adopting their mean opinion) if and only if their initial opinion distance is below a certain threshold $\epsilon$. This parameter is constant across the whole population and over time. In the reminder of the present work we often refer to it as the level of “open-mindedness” of the population because bounded confidence and open-mindedness both involve a willingness to consider different perspectives within certain limits. On the other hand, influenciability refers to being easily swayed by others, regardless of the strength of their arguments. Thus, we felt that open-mindedness was a more appropriate term for describing the bounded confidence threshold in our paper (for example as in^48^). However, it’s important to note that in opinion dynamics models, behavioral and psychological factors are often simplified and represented by model parameters. As a result, nuances can be lost and the bounded confidence threshold could also be interpreted as influenciability. To control the frequency of interactions with the media, we set a fixed probability $p_m$ - constant over time and across the whole population - which defines how likely it is to interact with a news piece (stubborn agent) after a user-to-user interaction. In our experiments, we assumed a mean-field context (e.g. all individuals can interact with all other agents without any social restrictions), which is a good starting point for analyzing the behavior of an opinion dynamics model. The model is detailed in Sect. "Model and methods".

The scenarios we analyzed in the present work are (i) a single moderate media ($x_m = 0.5$), to discover whether a “moderate message” would prevent the population from polarising in cases where it would happen without propaganda; (ii) extremist propaganda, where there is only one news source promoting a fixed extreme opinion (in this case, it was set to $x_m = 0.0$, but the same conclusions hold for 1.0); (iii) two polarised media sources, promoting two opinions at the opposite sides of the opinion spectrum ($x_{m1} = 0.05 \text { and } x_{m2} = 0.95$); (iv) finally, we also investigated a more balanced scenario where there are two polarised media sources (same as above) and a moderate one (promoting the central opinion of the spectrum, i.e. $x_{m3}=0.5$).

Without external effects, the population tends to: (i) polarise around moderately extreme positions (i.e. 0.2 and 0.8) when agents are “close-minded” ($\epsilon \le 0.32$); (ii) reach consensus around the mean opinion (i.e. 0.5) when agents are “open-minded” ($\epsilon > 0.32$), while the recommender system increases polarization/fragmentation, as shown in^30^.

In the remainder of this section, we analyzed these four different media landscapes and their effects on the opinion dynamics compared to the baseline model^30^.

### A moderate media in a biased environment favors the emergence of extremist minorities

**Figure 1:**
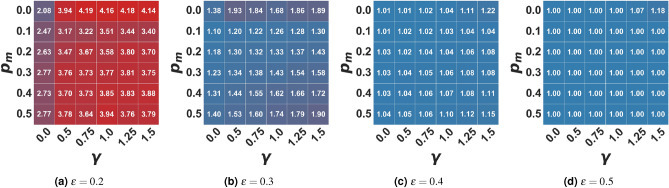
Average number of clusters in the moderate setting. In the figure, the average number of clusters of the final opinion distribution is represented as a function of the algorithmic bias $\gamma$ and the probability of user-media interaction $p_m$ for different bounded confidence values $\epsilon$. Values are averaged on 100 independent runs of each setting.

**Figure 2:**
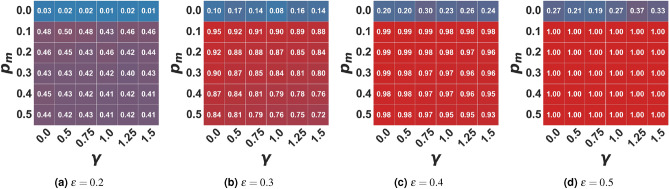
Average percentage of agents in the media cluster (0.5) in the moderate setting. In the figure, the average percentage of agents in the moderate cluster (0.5 +– 0.01) of the final opinion distribution is represented as a function of the algorithmic bias $\gamma$ and the probability of user-media interaction $p_m$ for different bounded confidence values $\epsilon$. Values are averaged on 100 independent runs of each setting.

In the first setting, we analyzed the effects of a “moderate message” on the opinion formation process, i.e. a single mass media promoting a central opinion ($x_m = 0.5$). We start from the hypothesis that such a media landscape may counteract the polarizing effects of a low bounded confidence $\epsilon$ or the fragmenting effects of a high algorithmic bias $\gamma$. Bounded confidence, as in the baseline model, can be so high that all agents are eventually drawn towards the same opinion (regardless of the strength of algorithmic bias), as in the case of $\epsilon =0.5$ (Fig. 1d). In general, in this setting, both cognitive and algorithmic biases maintain the effects they have in the baseline model: a higher confidence bound is more likely to push the population towards consensus, while a higher algorithmic bias increases the level of fragmentation in the final opinion distribution.

What emerged from our simulations is that, when interactions are not mediated by the recommender system ($\gamma =0$), **fragmentation** increases with the frequency of agent-to-media interactions: in fact, the average number of opinion clusters at equilibrium (see Fig. 1) increases with ($p_m$). Such tendency is due to the fact that, by increasing $p_m$, the portion of the population which initially has the media within their confidence bound moves towards such opinion faster than in the baseline model , thus reducing the probability of attracting agents at a distance greater than $\epsilon$ from the media that, in turn, will eventually stabilize around more extreme positions. When the social dynamic is, instead, mediated by a filtering algorithm, biasing the choice of the interacting partner towards like-minded individuals, the level of opinion fragmentation in the population is initially lower (for small $p_m$) with respect to the baseline model ($p_m=0.0$), but - likewise - it grows as agent-to-media interactions become more frequent. These results disprove our initial hypothesis that a “moderate” propaganda may straightforwardly counter polarization/fragmentation. Instead, promoting a single “moderate” opinion may not push the population to conform towards the desired point of view. Fragmentation is reduced only when the frequency of interaction with media is low. Otherwise, it also becomes a fragmenting factor.

Besides the number of clusters that coexist in the stable state, if we look at the whole opinion evolution process, we can see that there is always a portion of the population clustering around the media opinion (i.e. with opinion $x_i \in [0.5 +/- 0.01]$, while a small fraction assumes extremist positions. Figure 2 shows this cluster’s population percentage. The more open-minded the population and the higher the frequency of agent-to-media interactions, the larger the portion of agents that the media can rapidly attract towards the average opinion: thus, pushing the population towards consensus and countering the slowing down effect created by the algorithmic bias. Moreover, as we can see from Fig. 2, while in the baseline model, only a narrow portion of the population assumes the mean opinion, when a moderate media is promoting that opinion, we can see that the portion of the population ending in the moderate cluster in the steady state grows even with just a low probability to interact with the media and narrow open-mindedness threshold. Therefore, while consensus is not fully reached, a major cluster around the media is observed. Conversely, in the case of media absence ($p_m=0.0$), there is a higher variability in the final size of the moderate cluster. Even when a consensus forms, it is not necessarily around the mean opinion. Otherwise, the population polarizes around mildly extreme ones (around 0.2 and 0.8), avoiding the creation and maintenance of strongly extremist minorities, as it happens in the present model (see Supplementary Materials Figs. S8–S11).

However, when interactions are mediated by a filtering algorithm - $\gamma > 0$, the media can attract a smaller fraction of the population since agents holding more extreme opinions are much less likely to interact with those in the sphere of influence of the moderate media. Overall, our experiments showed that the algorithmic bias maintains its fragmenting power: specifically, as the bias grows, the extremist clusters that coexist with the moderate one increase in size, but also in dispersion, eventually splitting into multiple smaller clusters. At the same time, the fragmenting effect of the recommender system decreases the size of the moderates/neutrals cluster, especially in the case of moderately close-minded populations (Fig. 2), but not in a significant way (at least with the population size considered in the present work). We include in the Supplementary Materials a comprehensive analysis of results of simulations with different parameter settings.

### Extremist media shifts consensus in open-minded populations

**Figure 3:**
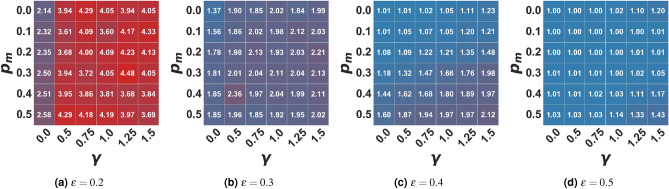
Average number of clusters in the extremist setting. In the figure, the average number of clusters of the final opinion distribution is represented as a function of the algorithmic bias $\gamma$ and the probability of user-media interaction $p_m$ for different values of $\epsilon$. Values are averaged on 100 independent runs of each setting.

**Figure 4:**
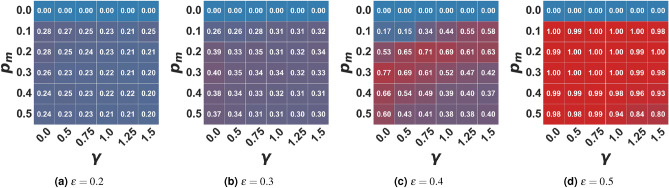
Average percentage of agents in the media cluster (0.0) in the extremist setting. In the figure, the average percentage of users in the extremist cluster ([0.0, 0.01]) is represented as a function of the algorithmic bias $\gamma$ and the probability of user-media interaction $p_m$ for different values of $\epsilon$. Values are averaged on 100 independent runs of each setting.

To investigate the effects of extremist propaganda and its effectiveness in shifting the consensus towards the desired opinion, we set the number of mass media outlets to $M=1$ and the promoted opinion to $x_m=0.0$.

Like in the moderate setting, the baseline model’s cognitive and algorithmic biases effects also remain in this setting. In the same way, an increase in the frequency of interaction with extremist propaganda (when $\gamma =0$) translates into an increase in the fragmentation of the final population. The number of clusters of the final opinion distributions, in fact, grows with $p_m$ (Fig. 3). For example, when the population is close-minded ($\epsilon =0.2$), in the absence of propaganda ($p_m=0$), in the final state, there are two main clusters (on average), while as $p_m$ increases, the number of clusters approaches 3. In the same way, as the population is more “open-minded” - so the number of clusters in the baseline model is lower - interacting with the propaganda still generates an increase in the number of clusters (moving the population from consensus around one opinion to clustering around two opinion values for $\epsilon =0.3$ and also $\epsilon =0.4$, even if in this case on average there is a consistent majority cluster). Despite the fact that an extreme opinion is promoted (while, without external effects, agents tend to conform to moderate positions), in this case, bounded confidence or, in other words, the level of “open-mindedness” of the population, can be so high that all agents are eventually drawn towards the same opinion, as in the case of $\epsilon =0.5$ (Fig. 3d). This fact still holds when the interactions are mediated by a recommender system ($\gamma > 0$), biasing the choice of the interacting partner towards like-minded individuals, but it is less evident due to the fragmenting power of the algorithmic bias. For example, when the population is close-minded, we tend to have an average of three or four clusters in a biased environment.

It is important to note that, compared to the moderate situation, the fragmenting effect of the external media is stronger for an extremist message. The number of clusters reported in Fig. 1 is generally smaller than that reported in Fig. 3.

In the present model, differently from the baseline^30^, i.e. $p_m=0.0$, the population splits into more than one cluster when $\gamma > 0$ and $\epsilon$ is sufficiently low. One of these clusters always forms around the extreme media opinion ($x_m=0.0$) while - as the bias grows - the rest of the population either clusters around a single value on the opposite side of the opinion spectrum or fragments into multiple small clusters (and their distance from the extremist propaganda increases with the open-mindedness of the population). This effect is stronger as the algorithmic bias increases and as the frequency of interaction with the media grows. In the case of extremist propaganda, as we can expect, a higher portion of the population in the stable state is an extremist, holding the same opinion promoted by the media (see Fig. 4). Additionally, the higher the open-mindedness of the population, i.e. the higher the confidence bound $\epsilon$, the higher the dimension of the extremist cluster - until ($\epsilon \ge 0.5$) the population is entirely attracted towards this extreme position (Fig. 4d). However, as the bias increases, the final number of opinion clusters increases, and the average number of agents in the extremist cluster decreases: the fact that algorithmic bias increases fragmentation in the population causes - in this case - the formation and maintenance of an “opposition” cluster (see also Figs. S18–S21 in the Supplementary Materials), countering the process of complete radicalization of the population. As the bias increases, of course, this cluster becomes more dispersed with respect to its average opinion, and for extreme biases, it fragments into a series of small opinion clusters. Therefore we can conclude that algorithmic bias acts as a partial protector against the message of one extremist media.

It is also worth noticing that, with $p_m > 0$, all other parameters being equal, the size of the extremist cluster does not increase with the probability of interaction with the media; on the contrary, the maximum size is reached for low or intermediate values of $p_m$ (see Fig. 4). Also, in this case, such behavior is tied to the fact that, even if the frequency of interaction with the media increases, those agents that initially are within the sphere of influence of the media will converge towards an extremist position more rapidly, thus losing the ability to attract those who are outside of it. When dealing with close-minded agents, less frequent propaganda can attract a higher fraction of the population with respect to more intense propaganda. If the population is open-minded, the frequency of interactions with the media loses most of its discriminant power: if at least half of the agents are already initially influenceable by the media, the whole population will converge towards the media opinion.

### Polarised media increase the divide

**Figure 5:**
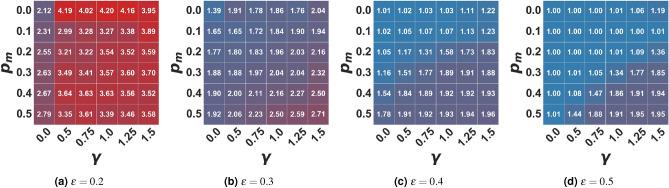
Average number of clusters in the polarised setting. In the figure, the average number of clusters of the final opinion distribution is represented as a function of the algorithmic bias $\gamma$ and the probability of user-media interaction $p_m$ for different values of the cognitive bias $\epsilon$. Values are averaged on 100 independent runs of each setting.

Public debates are often characterized by bi-polarity, a situation where two opposing views are proposed and debated. For example, media polarization in the U.S. has increased in the past half-decade, and both liberal and conservative partisan media are likely contributing to polarization in the Cable news networks^49^. While acknowledging that our synthetic setting represents a simplification of the complex dynamics at play, it nevertheless presents a scenario that merits further investigation. To recreate such a scenario - even if simplistically -, we simulated the presence of two extremist media outlets in the population, promoting opinions at the opposite sides of the opinion spectrum, - i.e. we set $x_{m1} = 0.05$ and $x_{m2}=0.95$. As expected, the presence of two polarised media increases the system’s polarization, which would already naturally arise due to the effects of the cognitive and algorithmic biases ($\epsilon \le 0.3$), but the presence of the media pushes the population towards the media opinions - which are more extreme than the ones that naturally form in the baseline model (see Fig. 5a,b and Fig. S24 in the Supplementary Materials). The presence of these two media, moreover, can bring the population towards polarization/fragmentation even in cases where the baseline model would predict full consensus ($\epsilon =0.4$), a fragmentation exacerbated by the recommender system effects (see Fig. 5c,d). On the other hand, in “close-minded” populations, the byproduct of agent-to-media interactions increasing the number of opinion clusters is that the rapid polarization of the extremes of the population results in the formation of a cluster of “moderate” agents, coexisting with polarized groups. On the one hand, this reduces the level of polarization in the population with respect to the baseline model. On the other hand, the polarized subpopulations are more extremist than in the baseline. As the filtering power of the recommender system increases, such a moderate cluster splits into multiple small ones, still concentrated around the center of the opinion spectrum (see Figs. S29–S32 in the Supplementary Materials for an example of the opinion evolution). Moreover, as the algorithmic bias grows, the two extremist clusters reduce their sizes, and more agents become neutral, even if they hold a wider range of opinions. This is because a reduced fraction of agents interacts with extremist media and/or peers that end up in the extremist cluster early in the process. Therefore, they cannot attract a more significant portion of the population with respect to the case where the filtering power of the recommender system is more robust. As the open-mindedness of the population grows, an increasingly stronger algorithmic bias is needed to maintain the moderate cluster, and, in most cases, the population tends to polarise, with the two sub-populations approaching the media opinions. The population is, in this scenario, ultimately radicalized around very extreme positions (0.05 or 0.95), similar to the case of a single extreme media. Finally, the recommender system makes the polarization process faster than what was observed in the baseline model, allowing fewer opinion clusters to coexist during the opinion dynamics.

### Open-minded populations are unstable in a balanced media landscape

**Figure 6:**
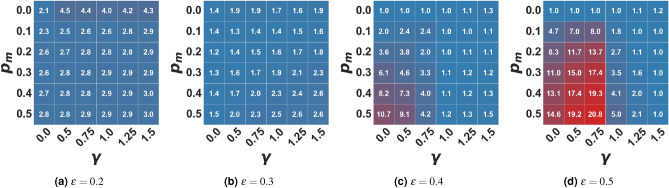
Average number of clusters in the balanced setting. In the figure, the average number of clusters of the final opinion distribution is represented as a function of the algorithmic bias $\gamma$ and the probability of user-media interaction $p_m$ for different $\epsilon$ values. Values are averaged on 100 independent runs of each setting.

In the last setting, we considered a more balanced information environment, with the presence of two extremist media in the population, promoting opinions at the opposite sides of the opinion spectrum, - i.e. we set $x_{m1} = 0.05$ and $x_{m2}=0.95$, alongside with a moderate media, with $x_{m3}=0.5$. In this setting, agents can retrieve from mass media both moderate and extremist points of view.

This more balanced news diet appears to still foster fragmentation. In fact, the higher the frequency of agent-to-media interactions, the more fragmented is the final population, as we can see from the average number of opinion clusters in the final population, which grows with $p_m$ (Fig. 6) and from the average pairwise distance, indicating how far are the peaks in the final opinion distribution (see Fig. S35 in the Supplementary Materials).

In this case, the algorithmic bias maintains its fragmenting power for a close-minded population (i.e. $\epsilon \le 0.3$). As the bias grows, the number of clusters increases, but it never exceeds three (Fig. 6a,b) since the population tends to rapidly converge towards the media opinions (see Figs. S42–S45 in the Supplementary Materials). The combination of a higher frequency of agent-to-media interactions, and the fact that interactions are biased towards similar opinions, allows each media to rapidly attract a portion of the population towards the promoted opinion.

On the other hand, in open-minded populations, $\epsilon \ge 0.4$, the relationship with the bias changes: from our experiments, it emerged that fragmentation is higher for low (Fig. 6c) or intermediate (Fig. 6d) values of the algorithmic bias $\gamma$, as the number of clusters in the final opinion distribution shows.

However, due to a stronger bias, the fragmentation that arises in the final state is not like the one reached in^30^. In that case, it was a stable state. In this case, the dynamic never reaches equilibrium, and agents keep changing their opinions influenced by the fixed opinions of the media. Nevertheless, in the cases where consensus can be reached, if open-mindedness is high, the dynamic is still unstable, and it takes a long time for the population to reach a consensus. Let us recall that the distance between two adjacent media is 0.45, so when $\epsilon =0.4$ agents holding an opinion between 0.10 and 0.45 or between 0.55 and 0.9 can be attracted by the moderate media and one extremist media that falls within their confidence bound and this generates an unstable stationary state preventing the system from reaching equilibrium. Obviously, the higher the open-mindedness, the higher the number of clusters (and the average entropy of the final distribution) since agents are distributed on a wider opinion spectrum, and real clusters do not form. This effect is counteracted by a high algorithmic bias, which practically impedes the interaction with the furthest media, even if in the range of the confidence bound.

### Algorithmic bias depolarizes discussion on EURO2020 “taking the knee” controversy

**Figure 7:**
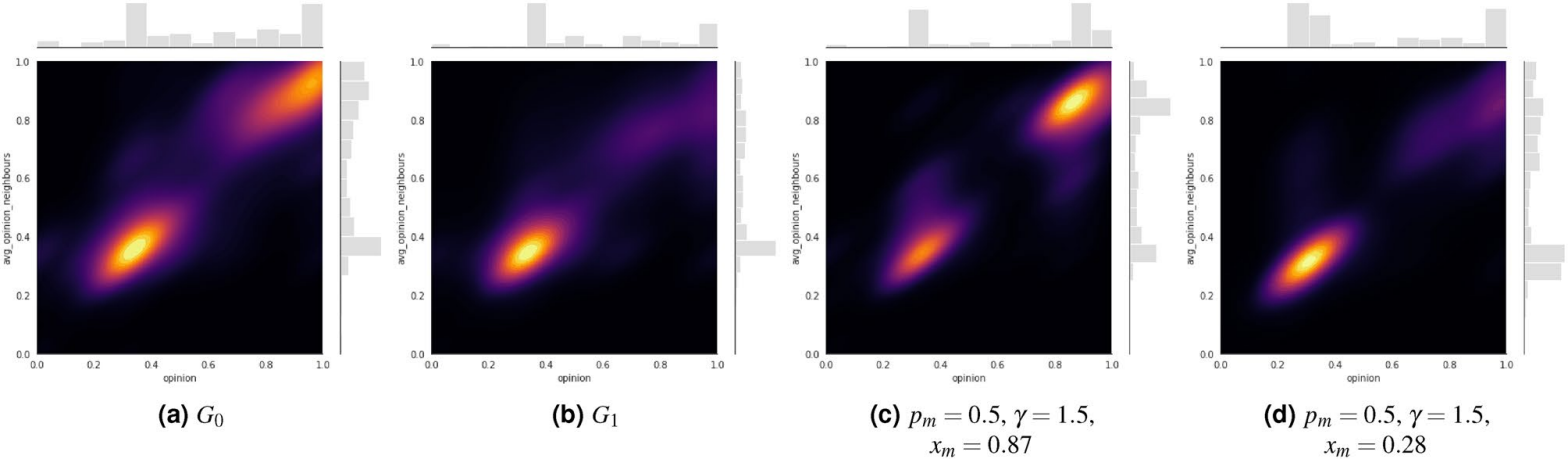
Joint distribution of the opinion of users and average leaning of their neighborhood. We display the first snapshot $G_0$ (initial matches)(**a**); the second snapshot $G_1$ (quarter-finals to final) (**b**); the final state of the simulation of the Algorithmic Bias Model with Mass Media and Heterogeneous Confidence Bounds with $p_m=0.5$, $\gamma =1.5$ and $x_m=0.87$(**c**); and the final state of the simulation of the Algorithmic Bias Model with Mass Media and Heterogeneous Confidence Bounds with $p_m=0.5$, $\gamma =1.5$ and $x_m=0.28$(**d**).

Despite trying to capture possible real dynamics with mathematical models of opinion formation, such synthetic settings may fail to capture peculiar characteristics of real networks, e.g. scale-free degree distributions and modular structures, but also polarized initial conditions, which may characterize discussions around controversial topics. Such diverse conditions may lead to different conclusions than the ones obtained in the mean-field case. For this reason, we exploited an empirical network collected from Twitter during EURO2020, where Italian users expressed their stances on the controversy around taking the knee in favor of the Black Lives Matter protests^50^. We simulated our model using this network as starting condition (both topology and initial opinion distribution) for different values of the model’s parameters. We include the results of simulations of the various settings in the Supplementary Materials, while here we discuss the most important ones. Our findings suggest that consensus may be reached in the final state when considering a homogeneous confidence threshold in scenarios with no media present or only a single media source. Even if such results are not averaged over multiple runs, these results may imply that scale-free degree distributions and modular topologies enhance consensus when the population has a homogeneous level of bounded confidence that is not lower than 0.2. However, an exception arises when there are no media sources, and a parameter value of $\gamma$=1.5 is applied. In this case, the final opinion distribution becomes fragmented, characterized by two main clusters centered around the average leaning of the “pro” faction and the average leaning of the “against” faction (see Supplementary Figs. 51–54). In this case, the bias may be too strong for users to converge toward a common opinion. When two polarized media sources are introduced (see Supplementary Fig. 55), opinions are concentrated around a moderate opinion in the final distributions. It exhibits a Gaussian shape, suggesting that the population tends to converge towards a common opinion in this case too. However, the presence of polarized media may keep users leaning toward more extreme positions. Adding a “moderate” media to this scenario, our observations reveal that the final opinion distribution remains symmetric and peaked around the center of the opinion spectrum. However, the distribution variance decreases compared to the previous scenario, i.e. people tend to homologate even more around a single opinion value, and variability is reduced. Furthermore, as the bias ($\gamma$) increases, the variance continues to decrease, and for $\gamma =1.0$, a single main opinion cluster emerges in the final state. Nevertheless, if the bias increases, e.g. $\gamma =1.5$, the final distribution splits into distinct opinion clusters centered around the media opinion. Moreover, since assumptions of homogeneous parameters are considered unrealistic, we exploited a methodology developed in^51^ to estimate user-level open-mindedness ($\epsilon _i$) and simulated a heterogeneous extension of our model. We include the results of simulations of this second set of experiments in the Supplementary Materials (see Supplementary Figs. 58–63), while here we discuss the most important ones. As displayed in Fig. 7a, users were embedded into echo chambers around pro and against stances on the discussion during the first two matches. However, when considering the period from the quarter-finals to the final (Fig. 7b), the same users are mainly clustered around positions in favor of kneeling, and polarization appears to be reduced. Simulations of our model, which exploits the first network as initial conditions of the simulations and accounts for heterogeneous levels of the confidence threshold estimated from the data according to the procedure in^51^, appear to confirm some of the insights offered by the mean-field analysis on the complete network with homogeneous parameters. The main conclusion that also holds in a real setting is that the algorithmic bias favors opinion fragmentation but, in doing so, helps to reduce the level of polarization of the network (see Fig. 7c and d) when there is an external source (or even a highly influential user) promoting one stance over the other. However, the setting that better captures the real opinion evolution can be seen in Fig. 7d, where a stubborn agent is promoting a fixed opinion aligned with the stance in favor of players “taking the knee”. However, in Fig. 7c, where the media is aligned with the opposite stance, the community that becomes less polarized is the other one, differently from the real situation.

## Discussion

A bounded confidence model of opinion dynamics with algorithmic bias and mass media agents was presented and studied in a mean-field setting. The model is an extension of the Algorithmic Bias model^30^ to include one or more mass media outlets. In the present work, media are modeled as stubborn agents, each promoting a fixed opinion and connected to every agent of the population. We analyzed four different settings, each representing a specific media landscape: in the first, a single moderate media is present; in the second, the single media supports extremist propaganda; in the third, two polarised media promote extreme and opposite opinions; and in the latter, a third media, promoting a moderate opinion, is added to the polarised setting. Our experiments reveal that mass media have an essential role in pushing people towards conformity and promoting the desired point(s) of view, but not in a straightforward manner, as adherence to the media message depends highly on cognitive and algorithmic bias and on the strength of the media itself. As we saw in the “moderate setting” (Sect. "Results"), an open-minded population tends to conform to moderate opinions, and only a few individuals will not. The main result of the “moderate message” is concentrating the central consensus cluster around the desired value. As expected, the size of the non-conforming clusters increases with algorithmic bias and decreases with open-mindedness. However, the size of the extremist nonconforming clusters also appears to increase in the strength of the moderate message. This is counterintuitive and indicates that, in general, not only the message has to be moderate, but also the frequency with which the message is presented has to be reduced. Moderation is necessary for all aspects to maximize adherence to the message.

Analyzing the results of the “extremist propaganda”, we saw that the power to push individuals towards the media opinion is not dependent on such opinion. In this case, the open-minded population tends to become extremist because agents are pushed toward the media opinion and conform to that value. Again, we observe that the maximum adherence to the media message is always obtained for moderate frequencies of interaction with media.

In a polarised media landscape, with two poles promoting extreme and opposite opinions, the more “open-minded” is the population - or, in other words, the easier it is to change peoples’ minds - the more likely the population will end up in one or two (oppositely) polarised extremist clusters. Also, in such a scenario, even when there would be a consensus around a moderate opinion, a higher frequency of interaction with the two extremist media is enough to push the population towards polarised stances, with two clusters forming around the media opinions.

In a balanced media landscape, when populations are close-minded, the more agents interact with mass media, the more they attract a portion of the population towards the promoted opinion. The effects of cognitive biases, i.e. bounded confidence, generally maintain the same role they have in the baseline model: the more “open-minded” is the population, the easier agents conform around the promoted opinion(s). However, when agents have access to multiple information sources (besides their peers’ opinions), “open-mindedness” leads to a population of indecisive individuals and unstable dynamics that prevent the system from reaching equilibrium.

Real network structures, characterized by scale-free degree distributions, modular structures, and polarized initial conditions, clearly impact the results of the dynamics of the present model. When open-mindedness is homogeneous across the population, users tend to converge towards a single opinion value, which depends on the initial average opinion and the opinion promoted by a single media. When the media landscape is more heterogeneous, i.e. media supporting two opposite stances, the population still tends to conform to a moderate stance. However, the final distribution has a higher variability, with some users maintaining more extreme leanings. Such variability is reduced when the media landscape actively promotes more moderate stances. In the case study, cognitive biases do not play a role in the result of the dynamics, while the role of the algorithmic bias remains the same as in the baseline model. However, when inferring open-mindedness levels from empirical data and using the real distribution of the parameter to simulate the model, results show final polarization distribution closer to the real ones, and the depolarizing role of the algorithmic bias emerges. Specifically, the real final state is well approximated by the setting where there is a recommender system biasing interactions and a mass media promoting an opinion aligned with the “pro-taking-the-knee” faction.

We typically give a positive value to a highly open-minded population, i.e. a population where agents have a high confidence bound. However, a higher open-mindedness in the presence of mass media may mean that the whole population is attracted to an extremist position, as we saw in the case of extremist propaganda or two polarised media. Even if the media is not extremist - it still means that the population conforms towards a single point of view, converging faster and perfectly towards a single opinion value, making agents subject to external control by those who can manipulate the information delivered by the media. Similarly, we usually give a positive value to the final consensus setting. However, as we already said, consensus also means conformity, homologation to a standard, which may be imposed from the outside and manipulated through media control to achieve the goals of those in power and hardly the optimal situation for our societies and democracies.

The large amount of research that has focused on detecting the strength and the effects of recommender systems and algorithmic biases moves from the idea that the presence of such biases traps users into echo chambers and/or filter bubbles, preventing them from getting confronted with a balanced information diet and thus polarising/fragmenting the population into a series of opinion clusters that do not communicate. Even though this is still far from being proven, even if we assume that this effect is true, it is worth asking ourselves whether this always has a negative effect. For example, from our work, it emerged that the presence of a recommender system alongside a moderate message facilitates the emergence and maintenance of extremist minorities, which coexist with a group of moderates. However, both a lower confidence bound, $\epsilon$, and a higher algorithmic bias, $\gamma$, when acting in a context where there is extremist propaganda or two polarised extremist media, avoid the complete radicalization/extremization of the whole population and counter the complete polarization by favoring the presence of a moderate cluster in both cases. We also observed that the recommender system facilitates convergence in a balanced setting where the population is open-minded. Indeed, it prevents the dynamic from being completely unstable - i.e. avoiding agents continuously changing their opinion and never reaching a stable state due to the presence of conflicting sources.

It is important to acknowledge that the identified effect of a recommender system is one of the potential outcomes, as also demonstrated through experiments conducted on a real network where two echo chambers were present. However, comprehending the full range of effects resulting from the actions of a recommender system involves considering multiple factors. Notably, this paper did not delve into the discussion of how incorporating the backfire effect^52^ (that can be seen as a kind of confirmation bias), in addition to bounded confidence, could potentially lead to increased polarization and contradict the original intentions of the approach, which aim to depolarize. Theoretical studies that assess the impact of recommender systems and design them with various objective functions to reduce polarization^53, 54^ often overlook the consequences arising from the interplay of different cognitive biases. Consequently, while we have numerous theoretical findings, their validity hinges on understanding how users interact with information and modify their opinions. Hence, insights into user behavior and opinion changes are vital. This, for example, motivated our investigation in^51^ to uncover the levels of cognitive biases exhibited by users within this discourse.

The present work is a preliminary step toward analyzing the interplay of social and media influence in digital environments and presents several limitations. We focused on mean-field scenarios, which prevents us from considering possible network effects on the results of the opinion evolution process. While this is a sound starting point, the obtained insights must be tested against different network structures or real networks to employ the proposed model to analyze and understand reality fruitfully. Moreover, social connections change in real settings, influencing subsequent interactions and opinion exchanges. As we did in^55, 56^ for the Algorithmic Bias model, network effects should be taken into account: greater sparsity in the underlying network structures appears to promote polarization and fragmentation in the Algorithmic Bias model, and it is possible that a similar effect may be observed in the model presented in this study.

We also saw in^55^ that mesoscale structure may promote different outcomes on the dynamic based on the different initial conditions. Here, we studied this model on a real network that exhibits two polarized communities. Experiments suggest that this may favor consensus even for lower confidence threshold levels. In order to verify this hypothesis, more convergence analysis needs to be performed on different modular networks and with different initial conditions. The present model could then be studied on adaptive network topologies to understand the interplay of the dynamics on/of the network. Moreover, in our work, bias has a role in the choice of the media only when in the presence of two or more sources. Even in the presence of a single externally promoted opinion, some agents who are too far away from that position may still have a small probability of interacting with it. To account for such a pattern, the probability of interacting with the media - which is now homogeneous across the whole population - could be made heterogeneous and dependent on the distance between the agent’s opinion and the promoted opinion and heterogeneous levels of agents engagement with mass media can be integrated within the model. Although all the different models demonstrate that an open-minded population can reach a consensus on all issues, it is an unrealistic assumption. Regardless of how open-minded they may be, each user will still have an inherent preference towards one side of the opinion spectrum. To address this, we propose extending the current model to incorporate a baseline opinion that consistently influences the user in that direction. Finally, as we saw in^51^, real populations may have heterogeneous (opinion-dependent) levels of “open-mindedness”, which could be taken into account to specify agents’ peculiarities better (as we did within the case study on the Twitter EURO2020 network), as well as heterogeneous activity levels as in^57^. Similarly to “open-mindedness” and activity levels, we plan to augment the current model with data-driven insights on media bias and user interactions with mass media and authoritative voices via online social networks. This will enable us to understand better the long-term impact of such interactions and how their influence differs from that of peers. One missing aspect in this context is undoubtedly a “dynamic” behavior from users, including the creation/destruction of links and the evolution of $\epsilon$ and $p_m$ with increasing/decreasing polarization. Additionally, there needs to be more evolution in the media’s behavior or a more realistic user-media relationship. The media should be aware of the cognitive biases of their users, and not all media outlets have the entire population as their audience. The more polarized the media are, the more likely they are followed by only a portion of the already aligned population, thereby promoting ideas aligned with that population segment. Another aspect not considered is that in a real setting, the “media” or stubborn agents may not be mainstream media with which everyone can interact but specific influential users within the network. This model would need to be adapted in such a scenario, considering that these stubborn agents are no longer connected to the entire population but only to certain nodes. Furthermore, the nodes they are connected to might depend on the opinions of those nodes and the opinions they promote. While our model has some drawbacks, as discussed above, it also has some advantages: it is simple, it can be tested on various topologies, it considers psychological, technological, and external factors, and it allows for flexibility in the number and opinions of the media.

## Model and methods

To introduce in the study of opinion dynamics the idea of a recommender system generating an algorithmic bias, the classical Deffuant-Weisbuch model^24^ was extended previously, implementing the Algorithmic Bias model (or AB model, hereafter)^58^. Our work is an extension of the AB model to include external information. In this section, we will first describe the AB model briefly before detailing our extension.

### The algorithmic bias model

In the AB model, we have a population of *N* agents, where each agent *i* has a continuous opinion $x_{i} \in [0,1]$. At every discrete time step, the model randomly selects a user pair (*i*, *j*), and if their opinion distance is lower than a predefined threshold $\epsilon$, $|x_{i} - x_{j}| \le \epsilon$, then the two agents change their opinion according to the following rule:

1 $$\begin{aligned} \begin{aligned} x_{i}(t+1)&= x_{i}(t) + \mu (x_{j}(t)-x_{i}(t)) \\ x_{j}(t+1)&= x_{j}(t) + \mu (x_{i}(t)-x_{j}(t)). \end{aligned} \end{aligned}$$

The parameter $\epsilon \in [0,1]$ represents the confidence bound of the population, which is assumed to be constant and equal for all agents. Individuals can only be influenced by those with similar opinions; a population with a low $\epsilon$ is said to be closed-minded; a high $\epsilon$, on the other hand, describes an open-minded population since it allows agents to influence each other even if their initial opinions are far away. The parameter $\mu \in (0, 0.5]$ is a convergence parameter, modeling the strength of the influence the two individuals have on each other or, in other words, how much they change their opinion after the interaction. Even if there is no reason to assume that $\epsilon$ and $\mu$ should be constant across the population or at least symmetrical in the binary encounters, these parameters are always considered equal for every agent.

The dynamics described above are those of the Deffuant-Weisbuch model, well known and studied by the opinion dynamics community. The numerical simulations of this model show that the qualitative dynamic is dependent on $\epsilon$: as $\epsilon$ grows, the number of final opinion clusters decreases. As for $\mu$ and *N*, these parameters influence only the time to convergence and the final opinion distribution width.

The AB model is different in how the interacting pair is randomly selected. It introduces another parameter to model the algorithmic bias: $\gamma \ge 0$. This parameter represents the filtering power of a generic recommendation algorithm: if it is close to 0, the agent has the same probability of interacting with all its peers. As $\gamma$ grows, so does the probability of interacting with agents holding similar opinions, while the likelihood of interacting with those who have distant opinions decreases. Therefore, this extended model modifies the rule to choose the interacting pair (*i*, *j*) to simulate a filtering algorithm’s presence. An agent *i* is randomly picked from the population, while *j* is chosen from *i*’s peers according to the following rule:

2 $$\begin{aligned} p_{i}(j)=\frac{d_{ij}^{-\gamma }}{\sum _{k \ne i}d_{ik}^{-\gamma }} \end{aligned}$$

where $d_{ij} = |x_{i}-x_{j}|$ is the opinion distance between agents *i* and *j*. For $\gamma = 0$ the model is equivalent to the DW-model.

**Figure 8:**
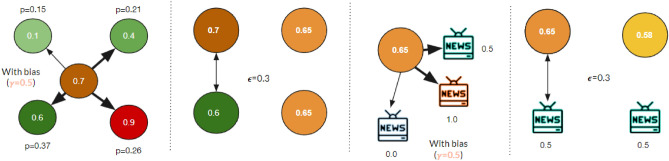
Example of agent-to agent and agent-to-media interaction with $\gamma =0.5$ **and** $\epsilon =0.3$. In the example, an agent with opinion 0.7 has a different probability to choose one of the four neighbors, represented by the thickness of the arrows in the figure. After changing opinions, due to the peer-to-peer interaction, the target agent chooses to interact with one of the three media, with a probability $p_m$. The choice of which media to interact with is determined according to $\gamma$, in the same way as in the social interaction: the higher the bias $\gamma$, the higher the probability to interact with a media promoting a closer opinion to the current one of the agent. If the media falls within the agent’s confidence bound $\epsilon$, the agent averages his opinion with the one of the media; otherwise, nothing happens. The media opinion, instead, remains unchanged.

### The algorithmic bias model with mass media agents

We now present our extension of the AB model, tailored to analyze the effects of mass media propaganda. We chose to model mass media as stubborn agents connected to everyone in the population, i.e. agents whose opinions remain fixed during the dynamic process and can interact with the whole population. This choice simplifies real-world media outlets that may instead change the promoted point of view, being influenced by public opinion or politics. However, we assume that our analysis is temporally constrained and that such changes are unlikely. A completely mixed population model that every individual can use any media - offline and online - as an information source. The fact that individuals often have a limited set of sources among which they choose is due mainly to cognitive and technological biases, which effects we are trying to capture with this model. Finally, we allow an arbitrary number of media sources *M* instantiated with custom opinion distribution $X_M$ to explore different scenarios in the present model.

To regulate the interactions with media outlets, we added another parameter, namely $p_m \in [0, 1]$, which indicates the probability that during each iteration of the model simulation - in addition to interacting with a peer - each agent interacts with a media $j \in M$ - always selected according to Eq. (2). So at each step, *t*, a peer-to-peer interaction takes place - as in the AB model - and with probability $p_m$, the selected agent interacts with a news source.

When two agents interact, their opinions change if and only if the distance between their opinions is less than the parameter $\epsilon$, i.e. $|x_{i}-x_{j}| \le \epsilon$, according to Eq. (1). However, when agent *j* is a mass media, only the opinion $x_i$ changes. Figure 8 illustrates an example of an interaction (both agent-to-agent and agent-to-media) and its effects on the node’s opinion in the presented model.

To conduct our experiments, we implemented the AB model with mass media within the NDlib^59^ Python library. This library has many opinion dynamics and epidemic models and a large user base. By adding our model to the library we increase the availability of our model to the scientific community.

### Analyses and measures

We simulate our model on a fully connected population of 100 agents, where the initial opinions are uniformly distributed, and we averaged the results over 100 runs. Like in^58^, to avoid undefined operations in Eq. (2), when $d_{ik} = 0$ we use a lower bound $d_{\epsilon } = 10^{-4}$. We imposed the simulations to stop when the population reaches an equilibrium, i.e. the cluster configuration will not change anymore, even if the agents keep exchanging opinions. We also set an overall maximum number of iterations at $10^6$ to account for situations where an equilibrium may never be reached. To better understand the differences in the final state, we studied the model for various combinations of the model parameters. We are interested in whether the different numbers and positioning of mass media and the growing interaction probability influence the final configuration, enhancing or reducing fragmentation and radicalizing individuals towards more extreme opinions, all other parameters being equal.

We replicated the work of^58^ by setting a null probability to interact with the media to define a reliable baseline for comparison.

In the simulations, we evaluated the model on every combination of the parameters over the following values:

- $p_{m}$ takes values in [0.0, 0.5], with steps of 0.1 - where for $p_{m}=0$ the model becomes the AB model.
- $\epsilon$ takes value in [0.1, 0.5], with steps of 0.1.
- $\gamma$ takes value in [0.5, 1.5], with steps of 0.25, and 0.0 - where for $\gamma = 0$ and $p_m=0$ the model becomes the DW-model.
- $\mu = 0.5$, so whenever two agents interact, if their opinions are close enough, they update to the average opinion of the pair.

We analyzed different scenarios to understand the effects of (i) one media, either extreme with a fixed opinion of $x_{m1}=0.0$ or moderate with an opinion of $x_{m1}=0.5$, (ii) two extremist media with $x_{m1} = 0.05, x_{m2}=0.95$ and (iii) two extremist media and a moderate one with opinions $x_{m1}=0.05, x_{m2}=0.5, x_{m3}=0.95$.

#### Measures

We used different measures to interpret the results, each equally necessary to understand the final state of the population. The first and most intuitive measure to understand fragmentation is the number of clusters present on average at the end of the dynamic. We used a naive clustering technique to partition the final opinion distribution into clusters: we sorted the final opinions in each run and set a threshold. Starting from one extreme, the corresponding nodes belong to two clusters every time two consecutive opinions exceed the threshold. Optimal results were obtained using a threshold of 0.01. Once we divided the population into opinion clusters we compute the cluster participation ratio, as in^58^:

3 $$\begin{aligned} C = \frac{(\sum _{i}{c_{i}})^{2}}{\sum _{i}{c_{i}^{2}}} \end{aligned}$$

where $c_i$ is the dimension of the *i*th cluster, i.e. the fraction of the population we can find in that cluster. In general, for *n* clusters, the maximum value of the participation ratio is *n* and is achieved when all clusters have the same size. At the same time, the minimum can be close to one if one cluster includes most of the population and a tiny fraction is distributed among the other $n \min 1$.

To grasp the attractive power of the media in each setting, we also computed the number of nodes present in the clusters centered on the media opinion. Specifically, we consider the percentage of agents that hold opinions in the range $[x_{m} - \lambda , x_m +\lambda ]$ with $x_m$ being the media opinion and $\lambda = 0.01$.

### Case study

The dataset used in this study spans approximately one month, from June 10th to July 13th, during which the EURO2020 matches were played. To focus our analysis on relevant conversations, we applied hashtag-based filtering, targeting discussions related to Italy’s matches, the tournament itself, and the topic of taking the knee. This filtering process yielded a collection of 38,908 tweets authored by 16,235 unique users.

We adopted a hashtag-based approach to infer Twitter users’ opinions regarding taking the knee during EURO 2020. A manual annotation process was employed to classify 2304 hashtags from the dataset. Each hashtag was assigned a numerical value based on its alignment with the pro or against stance, with $\pm 3$ indicating a clear position, $\pm 1$ indicating a close association, and 0 assigned to neutral or irrelevant hashtags. We calculated the non-neutral hashtag values within each tweet by averaging its classification value ($C_t$). Similarly, for each user (*u*), we computed their overall classification value ($C_u$) by averaging the classification values of their tweets. To facilitate integration with our opinion dynamics model, the initial pro/against scores, ranging from $-3$ to 3, were normalized to a range of [0, 1]. Additionally, we discretized the leanings into three categories: “Pro” (if $C_u \le 0.4$), “Against” (if $C_u \ge 0.6$), and “Neutral” otherwise, encompassing users with highly polarized viewpoints.

From the collected data, we constructed an undirected attributed temporal network, where nodes represent users and edges capture their interactions, including retweets, mentions, quotes, and replies. The resulting network comprises 15,378 nodes and 36,496 edges. To serve as initial and final states for validating our model, we divided the network into two snapshots: the first corresponding to the group stage and round-of-16, and the second representing the period from the quarterfinals to the final. This division was chosen based on specific reasons that will be further specified. As our model does not consider the temporal evolution of links, we retained only the nodes present in both snapshots. The temporal element was disregarded, resulting in two undirected snapshot networks: $G_0$, with nodes labeled according to their leaning in the first period, and $G_1$, with nodes labeled according to their leaning in the second period. This simplification aligns with our model’s assumption of a static network. The two snapshot graphs consist of 2925 users (approximately 20% of the total) and 9081 edges. Notably, the giant connected component comprises 2894 nodes and 9054 edges. For further details on the description and characteristics of the network, please refer to the Supplementary Materials.

#### Experiments on real data

The experiments were carried out with the following parameters:

- The underlying network structure is *G*: each node *u* in the interaction network is an agent *i* and each leaning $C_u$ in $G_0$ is an opinion $x_i$ with $x_i \in [0, 1]$.
- We tested both homogeneous and heterogeneous bounded confidence levels. For homogeneous values we considered$\epsilon \in \{0.2, 0.3, 0.4\}$; for heterogeneous values, each agent *i* is assigned with a level of bounded confidence $\epsilon _i$ obtained applying the procedure in^51^ (see Algorithm 1 in Supplementary Materials) to $G_0, G_1$.
- The parameter $p_m$ takes values of either 0.0 (absence of mass media, the model becomes the Algorithmic Bias Model with heterogeneous $\epsilon$) or 0.5.
- The parameter $\gamma$ varies in the range of [0.0, 1.5] with increments of 0.5; for $\gamma =0.0$, we obtain the Deffuant-Weisbuch model with heterogeneous $\epsilon$.
- The parameter $\mu$ is set to 0.5, i.e. when two agents interact, they adopt their average opinion.
- The maximum number of iterations is set at $10^5$.
- Simulations terminate when the maximum opinion change remains below a threshold of 0.01 for at least 500 consecutive iterations.

We performed a comprehensive analysis to examine the influence of different scenarios the opinion evolution. Our investigation encompassed five distinct media landscapes:

- One mass media with opinion $x_m = avg(pro) = 0.28$
- One mass media with opinion $x_m = avg(neutral) = 0.49$
- One mass media with opinion $x_m = avg(against) = 0.87$
- Two mass media with opinions $x_{m1} = avg(pro) = 0.28$ and $x_{m2} = avg(against) = 0.87$
- Three mass media with opinions $x_{m1} = avg(pro) = 0.28$, $x_{m2} = avg(against) = 0.87$ and $x_{m3} = avg(neutral) = 0.49$

Since in these experiments every agent *i* has a different level of bounded confidence $\epsilon _i$, to account for parameter heterogeneity, we applied the opinion change rule of the Algorithmic Bias Model with Mass Media in the following way:

- if $$d_{ij} < \epsilon _i$$ $$x_i(t+1) = (x_i(t) + x_j(t))/2$$
- if $$d_{ij} < \epsilon _j$$ $$x_j(t+1) = (x_i(t) + x_j(t))/2$$
- if if $d_{ij}> \epsilon _i \,\, \& \,\,d_{ij} > \epsilon _j$ nothing happens

i.e. a heterogeneous version of the baseline model.

Since we performed only one run per scenario, it is not feasible to compute the same metrics used in the mean-field analysis. Therefore, we choose to compare the simulation outcomes under various conditions on the real network with the actual opinion values in $G_1$. This allows for a direct assessment of the simulation results against the empirical opinion data at the specified time point. Specifically, we conducted one simulation for each scenario and compared the results with $G_1$ by examining the final states. To assess polarization and the presence of echo chambers in both real data and simulation outcomes, we adopted the approach presented in^60^. Supplementary Figs. 58–63 display plots showing the joint distribution of users’ opinions relative to the average leaning of their neighborhood, as obtained in our experiments. These plots provide insights into the formation of echo chambers within an interaction network by analyzing the behavior of individual nodes in relation to their neighbors’ behavior. As in^60^, we measure polarization in our simulation results based on the correlation between a user’s leaning and the average leaning of their nearest neighbors (ego network).

### Supplementary Information

Supplementary Information.

## Data Availability

The datasets generated during this study for simulations of the Algorithmic Bias Model with Mass Media in the mean-field case are within this article. The EURO2020 datasets analysed during the current study are available in the AlgBiasMediaModel repository, https://github.com/ValentinaPansanella/AlgBiasMediaModel.git.

## Code availability

The AB model with mass media implementation used to conduct our experiments is the one provided by the NDlib^59^ Python library: http://ndlib.rtfd.io. Simulations can be reproduced using the code in: https://github.com/ValentinaPansanella/AlgBiasMediaModel.git.
