# Supplementary material for: Mass media impact on opinion evolution in biased digital environments: a bounded confidence model
Source: Sci Rep. 2023 Sep 5;13:14600. doi: 10.1038/s41598-023-39725-y (PMC10480185; doi:10.1038/s41598-023-39725-y)
Supplement: Supplementary file 1 — Supplementary Information. [file 41598_2023_39725_MOESM1_ESM.pdf]

Supplementary Materials for ”Mass Media Impact on Opinion Evolution in Biased Digital Environments: a Bounded Confidence Model”

Valentina Pansanella[1,4,*], Alina Sîrbu[2], Janos Kertesz[3], and Giulio Rossetti[4]

[1]Faculty of Science, Scuola Normale Superiore, Pisa, Italy
[2]Department of Computer Science, University of Pisa, Largo Bruno Pontecorvo 3, Pisa, Italy
[3]Department of Network and Data Science, Central European University, Vienna, Austria
[4]Institute of Information Science and Technologies “A. Faedo” (ISTI), National Research Council (CNR), G. Moruzzi 1, Pisa, Italy
[*]valentina.pansanella@sns.it

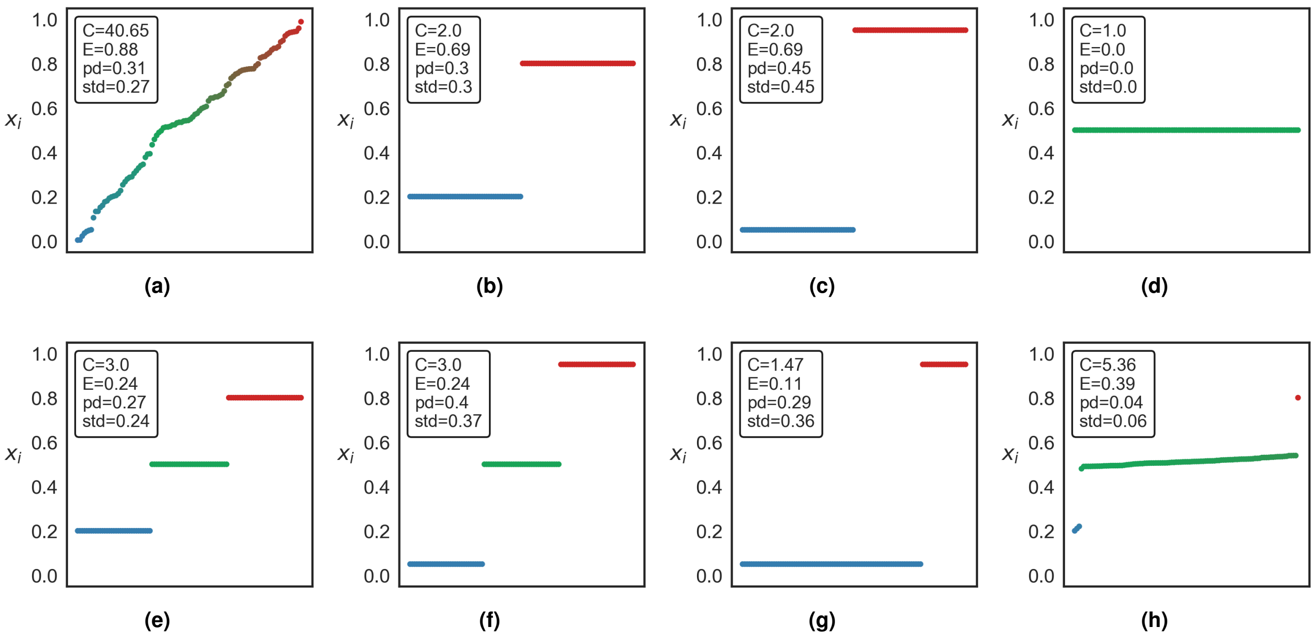

Supplementary Figure 1. Final opinion distributions examples with associated measures. In the figure, we compared different final states to highlight the changes in the measures used in the present work. We can note that: 1) the average pairwise distance and the standard deviation give insights about intra-clusters distance, while the number of clusters and the normalized entropy remain constant ((b)-(c) and (f)-(g)), and 2) the number of clusters does not well capture situation where opinions are distributed within a broader range of values, thus why we also compute the normalized entropy.

To better understand why we need to consider multiple measures simultaneously to correctly characterize the final state of the population and understand the dynamics as the parameters of the model vary, in Supplementary Figure 1 we plotted different possible final opinion distributions along with the metrics we previously described. Starting from the upper-left panel (a): in this case, the final opinions are uniformly distributed, which means that the level of normalized entropy close to the maximum possible, i.e., 1.0, (in fact, it is 0.88) and the computed number of clusters is $C \approx 40$ (however, we can see that there are not 40 clusters, since we can not correctly identify clusters in such a final state). Instead, we need to interpret this situation as agents *unable* to cluster around few opinion values. Comparing panel (a) and (b), we can see that the values of the average pairwise

distance and the standard deviation of the final opinion distribution are very similar. However, the distributions are significantly different. Such peculiarity emerges when considering the value of the normalized entropy (0.69) and the number of clusters (2, which indicates two clusters of similar size). Comparing (b) with (c), we can see that in both cases, polarization arises; however - in the second - polarization is “stronger”, i.e., the two opinion cluster are more distant than in the previous case. For this reason, while the normalized entropy and the number of clusters have similar values, the average pairwise distance and the standard deviation are higher. Finally, in panel (d), we can see that in the case of a perfect consensus entropy, pairwise distance and standard deviation are equal to 0 and that the number of clusters is, as expected, 1. In the second row, we can observe that, although in panel (e) and (f) we have three clusters (the normalized entropy is 0.27 and the number of clusters is 3.0 in both cases), the fact that the average pairwise distance and the standard deviation are higher in the latter panel allows us to understand that these clusters are more apart than in the former one. In panel (g), however, a consensus around a single opinion does not emerge; however, the effective number of clusters (1.47) allows us to account for the fact that one of them is bigger than the other - the same goes for the fact that the normalized entropy is lower with respect to panel (b) or (c) for example, where the two clusters had the same size. Even if the two clusters are almost as distant as in panel (c), the average pairwise distance and the standard deviation are lower because the majority of agents belong to one of them. Finally, in the panel (h), we can see that, at first glance, the population has reached consensus (except for a few outliers); However, the number of clusters is 5.36. This latter result describes how agents could not agree around a single opinion; instead, they clustered in a range of values, which our model identifies as multiple clusters (even though they are not properly so). However, this situation can be correctly sorted out by focusing on the number of clusters and entropy (both pretty high) along with pairwise distance and standard deviation, which are lower than in the case where there were two clusters.

Moderate media landscape with algorithmic bias

In the following section, we present additional figures and metrics referred to the first setting. In the first setting we simulated the presence of one moderate media in the population promoting $x_{m1} = 0.5$. We can see from the heatmaps in Supplementary Figure 2 that: a higher confidence bound reduces the average normalised entropy of the final opinion distribution, while a higher level of the algorithmic bias increases it. More specifically, we can see that when $\varepsilon = 0.2$ (Supplementary Figure 2 (a)) in the Deffuant model the average normalised entropy is 0.2 (polarisation), which increases as p_m grows (up to 0.27 for $p_m = 0.5$). While in the Algorithmic Bias model the average entropy moves from 0.2 to 0.5 when adding the filtering algorithm ($\gamma > 0$), in the case of a moderate media the increase is lower for $p_m = 0.1$ (from 0.23 to 0.41). Moreover, as p_m increases, the effect of the bias in this case is less strong, and we have similar values of normalised entropy in any case. When $\varepsilon = 0.3$ (Supplementary Figure 2(b)) we can see that the average normalised entropy in the Deffuant model is 0.09 (consensus), but it is lower (0.04) when $p_m = 0.0$ and then grows as p_m grows. Adding bias to the picture, we can see that the average normalised entropy grows both as a function of p_m and as a function of γ. The same can be said for $\varepsilon = 0.4$ and $\varepsilon = 0.5$, but starting from lower values of average normalised entropy. Moreover, we can also see that the increase due to the bias is less strong when there is a small/intermediate p_m.

In Supplementary Figure 3 we can see the average number of clusters, computed as we explained in section Methods. We can see that for $\varepsilon = 0.2$ (Supplementary Figure 3(a)) in the baseline model we go from 2 to 4 clusters when considering a positive algorithmic bias. We can see that the number of clusters gradually increases as p_m grows, and that the bias has less power for low/intermediate p_m. The average number of clusters is always around 3 or 4 in this case. When $\varepsilon = 0.3$ (Supplementary Figure 3(b)) we can see the same pattern, however starting from a situation of consensus in the baseline model and at most 2 cluster form for high p_m and γ. For $\varepsilon > 0.3$ consensus is always reached.

As we can see from Supplementary Figure 5 the average standard deviation of the final opinion distribution decreases with ε and increases with γ, both in the baseline model and in the extension presented in this work, with $p_m > 0$. The same conclusions on the levels of polarisation of the system can be drawn looking at the average pairwise distance in Supplementary Figure 4.

As we can see from Supplementary Figure 6 convergence is on average faster when the population interacts with a moderate media and the algorithmic bias loses its slowing down effect on the dynamic .

From Supplementary Figure 7 we can see that in the baseline model only a small fraction of agents clusters around the mean opinion (and this fraction increases with ε). However, if we add a stubborn agent with fixed opinion $x_m = 0.5$, this fraction increases significantly. However, we can see that it is not directly proportional to p_m, on the contrary, it decreases both with p_m and with γ.

As we can see from Supplementary Figure 8 when $\varepsilon = 0.2$ in the baseline model either 2 or 3 final clusters arise in the population when there is no bias; as the bias grows fragmentation in the final distribution rises and the process of convergence slows down. The effect of placing a moderate media interacting with the population is to attract a portion of the population towards the mean opinion, i.e. 0.5. However, this portion is smaller and smaller as the bias grows, due to the fact that a smaller portion of agents will likely interact with those agents who are influenced by the media. Also, while with a low bias other two polarised clusters form, as the bias grows still two clusters form, but instead of having all agents holding a single opinion value these cluster are more spread and eventually they split into multiple opinion clusters.

From Supplementary Figure 9 we can see that the presence of a moderate media contrasts the slowing-down effect of the algorithmic bias and reduces the levels of fragmentation with respect to the baseline model with no media. A central cluster always forms, and normally two polarised small clusters appear at the extremes of the opinion spectrum. Due to this fact fragmentation with low level of biases, where in the baseline model there would be consensus or polarisation, is now higher, because two polarised cluster and a moderate one now form. Moreover, the polarised clusters are now pushed to the extremes more than in the baseline model. As the bias grows in the baseline model convergence slows down and fragmentation arises, while with media interaction the population is still split in somehow three cluster that are more spread within themselves as the bias grows.

From Supplementary Figure 10 we can see that the behaviour is very similar to the one described for Supplementary Figure 9 but fragmentation is overall reduced due to the increase in the level of the confidence bound.

Finally, when $\varepsilon = 0.5$ (i.e. Supplementary Figure 11 we can see how media interaction make convergence to consensus faster and reduce fragmentation that would arise in the baseline model.

Extremist media landscape with algorithmic bias

In the following section, we present additional figures and metrics referred to the second setting. In the second setting we simulated the presence of one extremist media in the population promoting $x_{m1} = 0.0$, to simulate a situation where there is an extremist propaganda. As we can see from Supplementary Figure 12, the average normalised entropy in the final opinion distribution decreases as ε increases, but there is no ε in the considered range for which consensus is always perfect. We can also see that - in this setting - the main driver of fragmentation, i.e. increase in average entropy, is the algorithmic bias, especially for $\varepsilon \leq 0.3$: for $\varepsilon = 0.2$ the average normalised entropy is always around 0.5, with no significant increase/decrease as p_m and γ vary; in the case of $\varepsilon = 0.3$ entropy values are lower (around $0.2/0.3$) but still there no significant dependence between entropy and p_m and γ. In the case of a higher ε we can see instead that the entropy level increases gradually as both γ and p_m increase. For $\varepsilon = 0.5$ consensus is always reached (i.e. entropy = 0.0) except for very high levels of p_m and γ.

As we can see from the average number of clusters (Supplementary Figure 13), if we disregard the Deffuant case (i.e., $\gamma = 0.0$), the average number of clusters is in general higher when there are no agent-to-media interactions. As in the baseline model, the number of clusters decreases as the bounded confidence ε increases, while is instead directly proportional to the algorithmic bias γ. For $\varepsilon \leq 0.4$ there is always a level of bias for which fragmentation arises in the population, while for $\varepsilon \geq 0.5$ fragmentation only arises when there are no media interactions, while the presence of the mass media either favours consensus (for low bias) or polarisation (for higher bias).

From the average pairwise distance (Supplementary Figure 14) and the average standard deviation (Supplementary Figure 15) we obtain the same information: both measures grow with p_m and γ, i.e. in this case the higher the number of clusters (or average normalised entropy) the higher the average pairwise distance (or average standard deviation).

The average number of iterations at convergence, instead, increases with the algorithmic bias (as in the baseline model). However, we can see from Supplementary Figure 16 that the dynamic is slower for low values of p_m (e.g. $p_m = 0.1$) and - in this case - the higher ε, the slower the convergence.

As the number of clusters increases with p_m and γ, this does not happen with the average size of the extremist cluster. The size of this cluster - as we can see from Supplementary Figure 17 - depends mainly on ε: for $\varepsilon = 0.2$, 20-30% of agents are extremist, this fraction increases to 30-40% when $\varepsilon = 0.3$, while for $\varepsilon = 0.4$ we can have from 10 to 80% of extremist agents in the final state. Lastly, for $\varepsilon = 0.5$ we have around 80-100% of extremist agents. Additionally, we can see that there are cases where the size of the extremist cluster decreases with γ, while in other cases it grows with γ. This latter behaviour, in particular, can be seen for low p_m (i.e. $p_m = 0.1$ or $p_m = 0.2$) and $\varepsilon = 0.3$ or $\varepsilon = 0.4$.

Polarised media landscape with algorithmic bias

In the following section, we present additional figures and metrics referred to the third setting. In the third setting we simulated the presence of two extremist media in the population promoting opinions at the opposite sides of the opinion spectrum, i.e. we set $x_{m1} = 0.05$ and $x_{m2} = 0.95$, to simulate a situation where there is a polarised media landscape. As predictable, the presence of two polarised media in a bounded confidence model increases the level of polarisation in the system which would already naturally arise due to the effects of the cognitive biases, pushing a population that would already polarise in the baseline model ($\varepsilon \leq 0.3$) towards the media opinion which are more extreme than the ones that would form in the baseline model (normally around 0.2 and 0.8 and not further) as proven by the fact that the average pairwise distance is higher when $p_m > 0$. When the population is close minded ($\varepsilon = 0.2$, see Supplementary Figure 22(a) and 23(a)) the population in the final state splits into more than two opinion clusters. As we can see from the heatmaps, in the baseline model with no media and no algorithmic bias, there is an average normalised entropy of 0.2 and an average number of cluster of 2, meaning that population mainly separates into two clusters of similar sizes, creating a polarised final population (as we can see from the fact that the average standard deviation of the final opinion distribution is 0.24 (see Supplementary Figure 25) and the average pairwise distance is 0.12 (see Supplementary Figure 24). We can see that, when $\gamma = 0.0$ the average normalised entropy increases with p_m from 0.21 to 0.26, which corresponds to an increase in the average number of clusters from 2.31 to 2.79 (which means that on average there are three clusters of different sizes with one bigger than the others). We can see that the average pairwise distance and the average standard deviation do not increase with p_m significantly, due to the fact that when the population splits from two to three clusters the average distance between the two more extremist clusters and the moderate one is less than the distance between two polarised clusters (so the average pairwise distance ranges from 0.18 to 0.19 and the average standard deviation between 0.35 and 0.36). We can see that, given a certain p_m, the fragmenting power of the algorithmic bias brings rapidly the population to split into three clusters, which become four in some cases, however there is not a clear pattern for which the average number of clusters increases as a function of p_m or γ when $\gamma > 0$: we can see that the number of clusters increases with γ when $p_m \leq 0.3$ and then it somehow decreases with γ. However, looking at the aerage normalised entropy we can see that all the values for $\gamma > 0$ we can see that it increases with γ for $p_m = 0.1$ from 0.32 to 0.42, but for any other value of p_m and γ we have similar entropy values ranging from 0.35 to 0.39 indicating similar levels of fragmentation. If we look at the average pairwise distance and the average standard deviation we can see that: the average pairwise distance decreases with p_m and increases with γ for $p_m = 0.2$ then it decreases with γ. Same happens with standard deviation which decreases both with p_m and γ in this case. In this case (remember we are talking about a close minded population with $\varepsilon = 0.2$ we can see from Supplementary Figure 26 that the convergence slows down for $p_m = 0.1$, with respect to the baseline case, and then it speeds up both as the probability of media interaction increase and as the bias increases. We can also see from Supplementary Figure 27 that, with respect to the baseline case with no media, there is always a positive fraction of the population holding an opinion in the cluster of one of the extremist media. As the filtering power of the recommender system increases, i.e. as the probability of interacting with like minded individuals is higher, this moderate cluster splits into multiple small opinion clusters, still around the center of the opinion spectrum. Moreover, as the algorithmic bias grows, the two extremist clusters become smaller and smaller and more agents become moderate/neutral. This is due to the fact that less people interact with extremist media and/or agents that ended up in the extremist cluster in the early stages of the process and so they are not able to attract a larger portion of the population with respect to the case where the filtering power of the recommender system is less strong. As the open mindedness of the population grows, a stronger and stronger algorithmic bias is needed to maintain the moderate cluster and in most cases the population is polarised and the two sub-populations hold the media opinions, until the level of open mindedness is high enough that the population is able to reach consensus which, however, now forms around the opinion of one of the two media: the population is completely radicalised around very extreme positions (0.05 or 0.95) like it happened in the case of one extreme media. Moreover, the recommender system in this case makes the process of polarisation faster than in the baseline model and there is less coexistence of multiple opinion clusters during the process (Supplementary Figures 29-32).

Balanced media landscape with algorithmic bias measures

In the following section, we present additional figures and metrics referred to the fourth setting. In the fourth setting we simulated the presence of three media and we set $x_{m1} = 0.05$, $x_{m2} = 0.95$ and $x_{m3} = 0.5$, to simulate a situation where there is more balanced media landscape.

We can see from Supplementary Figure 33 that, without media interactions, the average normalised entropy grows with γ and decreases with ε. More in detail we can see that for $\varepsilon = 0.2$ and $\gamma = 0.3$ even low values of γ are enough to go from polarisation to fragmentation with a strong increase in entropy, which then remains more or less stable as the bias grows. Instead, for higher ε the increase in entropy is more gradual as the bias grows. When considering media interactions ($p_m \geq 0.1$) we can see that the increase in entropy is more gradual as the bias and p_m grow, in close-minded populations ($\varepsilon \leq 0.3$), while for open-minded populations ($\varepsilon \geq 0.4$) the dynamic is different. In particular, the average entropy still increases as the probability of media interactions increases, however: we can see that for $\varepsilon = 0.4$ the average normalised entropy decreases as the bias grows, while for $\varepsilon = 0.5$ it increases up to a certain value of γ and then it decreases until reaching 0.0, i.e. perfect consensus, for $\gamma \geq 1.5$.

We can see from the average number of clusters (Supplementary Figure 34) that the same insights we obtained by looking at the average normalised entropy can be seen from the average number of clusters. In particular, we can see that without media interactions, the number of clusters increases with γ and decreases with ε. In particular, we can see from heatmap (c) and (d) that for $\varepsilon \geq 0.4$ we always obtain consensus, despite the strength of the bias. In any case the number of cluster grows with p_m. However, for $\varepsilon \leq 0.3$ we generally obtain at most three clusters in the final state, while for $\varepsilon \geq 0.4$ we obtain a high number of clusters (however from this metric we cannot understand if these are proper opinion clusters or if few "cluster" have just a higher opinion range). Moreover, we can see that, for $\varepsilon \geq 0.4$, as the bias grows, the number of clusters decreases (the higher the confidence bound, the higher the bias needed to bring the population back to a situation of polarisation or consensus).

From the average pairwise distance (see Supplementary Figure 35 we can see that it increases with p_m and γ, when $\varepsilon \leq 0.3$, otherwise it follows the same pattern than the number of clusters (or average entropy). However, we can see that, in some cases, despite the number of clusters is lower, the average entropy is higher: this is because fewer clusters form that are more far apart from each other in the opinion space. We can also notice that, when we have a high number of clusters, the average pairwise distance is relatively low, since opinions are not well separated into clusters and - on average - agents are closer than they would be if they were polarised.

As we can see from the standard deviation of the final opinion distribution (Supplementary Figure 36, for low open-mindedness it increases both with γ and p_m, while for $\varepsilon = 0.4$ it increases with γ and for $\varepsilon = 0.5$ it increases with γ until $\gamma = 0.75$ and then it decreases with γ.

From Supplementary Figure 37 we can see that the speed of he dynamic does not change much for close-minded populations, we can see just a slight slowdown for $p_m = 0.1$ and $p_m = 0.2$ when $\varepsilon = 0.3$. For open-minded populations, instead, we can see that the dynamic more than slow is unstable in the sense that - besides having a strong bias - agents keep changing their opinions and never reach equilibrium. As we can see from the heatmaps, in most cases the simulations stop because we reach the maximum number of iterations set in our experiments, and not because the population reaches equilibrium.

As we can see from the heatmaps in Supplementary Figure 37 for low bounded confidence the process slows down for small values of p_m. For high bounded confidence we can see that the cases where we have fragmentation or the highest number of cluster in the final state have the slowest time to convergence and we can see from the values that in most cases the process does not reach equilibrium but it is stopped due to reaching the maximum number of iterations.

We can see from the heatmaps (Supplementary Figure 38 that the average opinion is generally always around the mean of the opinion spectrum. There is just one case for which the population seems to concentrate more around a lower opinion range (0.3).

We can see from the heatmaps (Supplementary Figure 39) that in the case of $\varepsilon = 0.2$ the percentage of agents in the moderate cluster is always around 40% when the population can interact with the media, the same cluster is bigger when $\varepsilon = 0.3$, but its dimension decreases with p_m and γ, because both parameters tend to increase the probability that one agent interacts more often with one media and so that some agent cluster around different opinions. When $\varepsilon = 0.4$ this cluster becomes smaller with p_m buut instead grows until $\gamma = 1.0$, then slightly decreases with γ. Finally, when $\varepsilon = 0.5$ the size of this cluster decreases with p_m and grows with γ until the population always reaches consensus around the moderate opinion for $\gamma = 1.5$.

In Supplementary Figure 40 we can see that in the case of $\varepsilon = 0.2$ the percentage of agents in the 0.05 cluster is always around 30%, this percentage grows with p_m and γ when $\varepsilon = 0.3$ and $\varepsilon = 0.4$, but in this latter case is always close to 0.0, while for $\varepsilon = 0.5$ it is always 0.0.

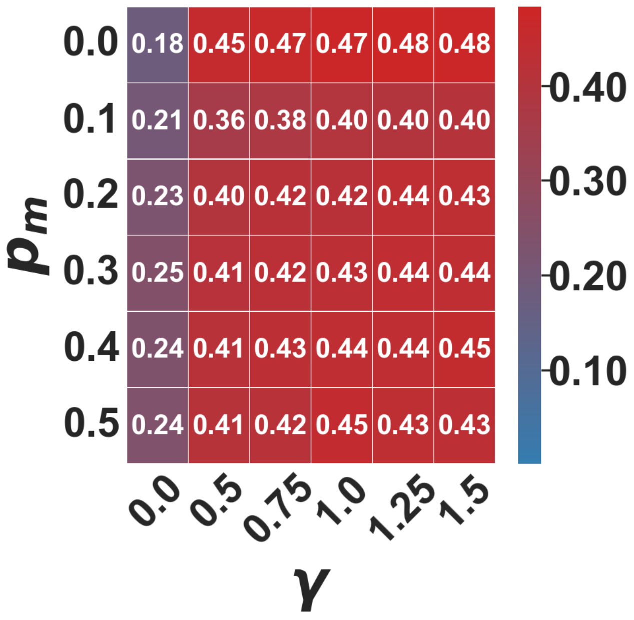

(a) $\varepsilon = 0.2$

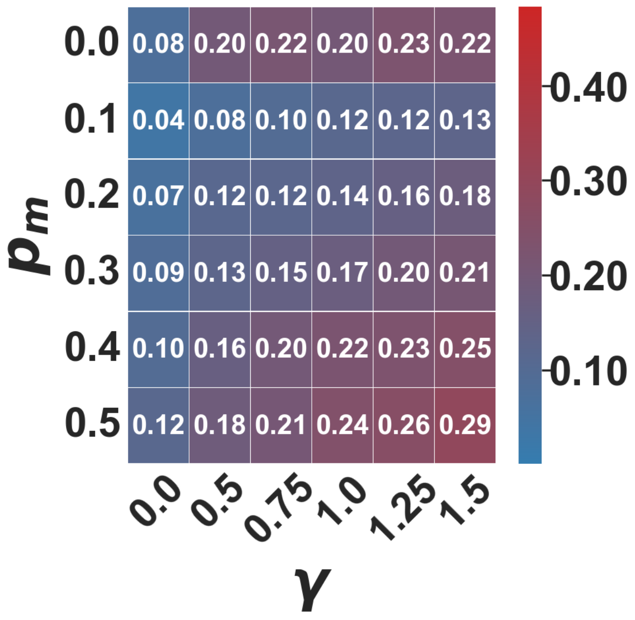

(b) $\varepsilon = 0.3$

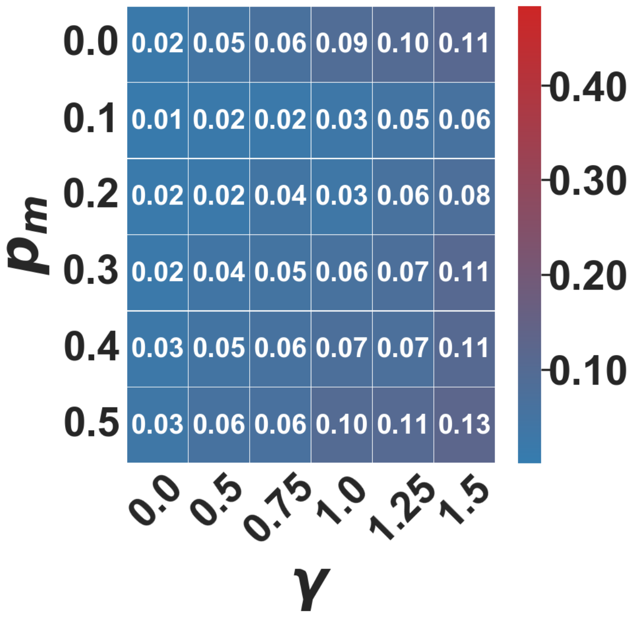

(c) $\varepsilon = 0.4$

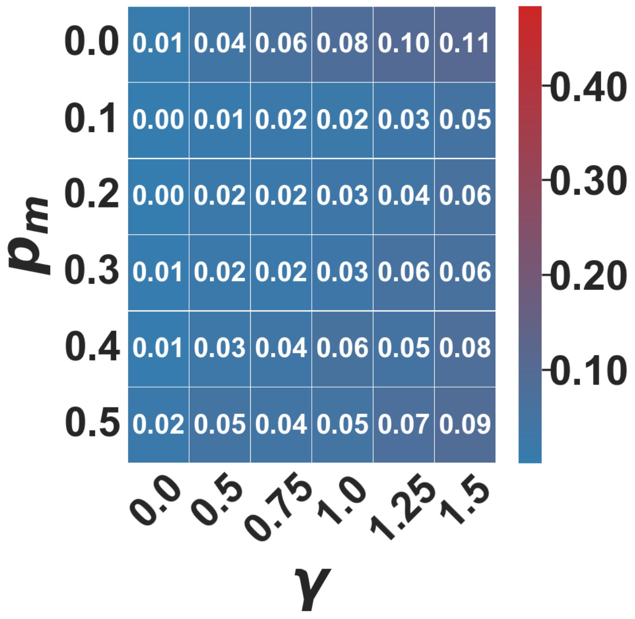

(d) $\varepsilon = 0.5$

Supplementary Figure 2. Average normalised entropy in the Algorithmic Bias model with media interactions for different values of ε as a function of γ and p_m in the moderate setting. In the figure the average values of the entropy of the final opinion distribution are represented as a function of the algorithmic bias γ and the probability of user-media interaction p_m and are normalised in the range $[0,1]$ where 0 means that every agent holds the same opinion and 1 means that final opinions are uniformly distributed. Values are averaged on 100 independent runs of each setting.

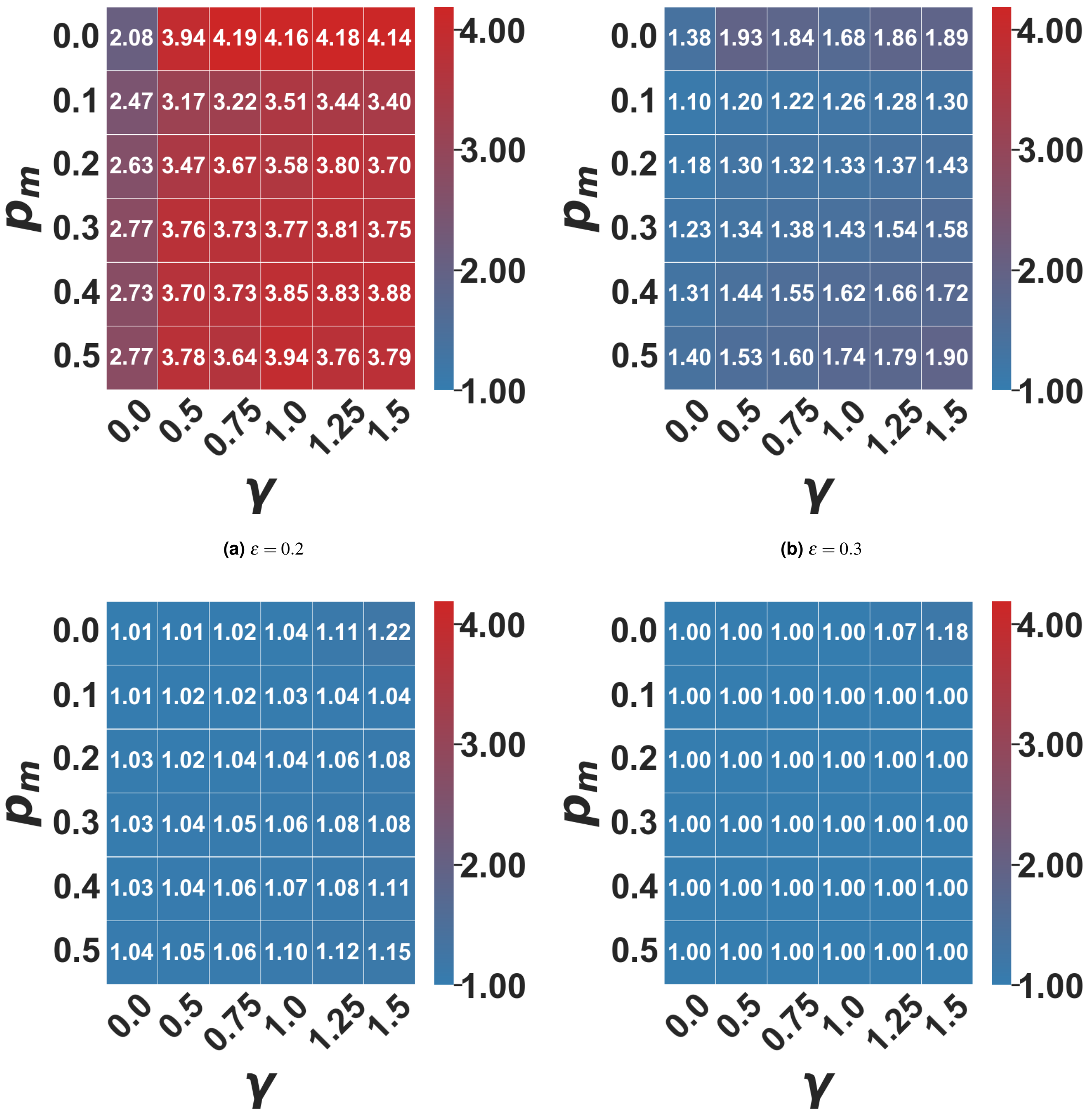

(a) $\varepsilon = 0.2$ **(b)** $\varepsilon = 0.3$

(c) $\varepsilon = 0.4$ **(d)** $\varepsilon = 0.5$

Supplementary Figure 3. Average number of clusters in the Algorithmic Bias model with media interactions for different values of ε as a function of γ and p_m in the moderate setting. In the figure the average number of clusters of the final opinion distribution are represented as a function of the algorithmic bias γ and the probability of user-media interaction p_m. Values are averaged on 100 independent runs of each setting.

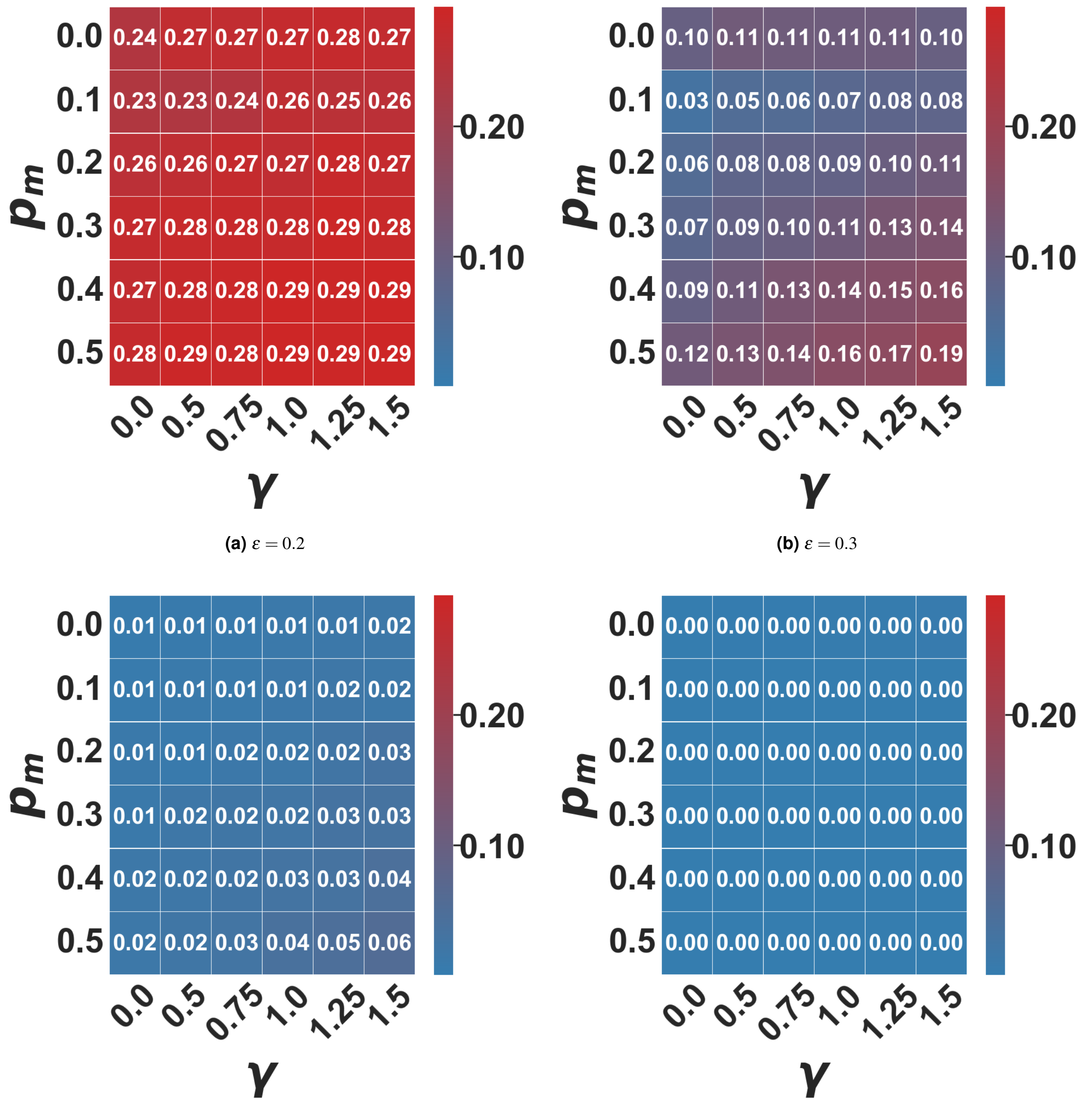

(a) $\varepsilon = 0.2$

(b) $\varepsilon = 0.3$

(c) $\varepsilon = 0.4$

(d) $\varepsilon = 0.5$

Supplementary Figure 4. Average pairwise distance of the final opinion distribution in the Algorithmic Bias model with media interactions for different values of ε as a function of γ and p_m in the moderate setting. In the figure the average pairwise distance of the final opinion distribution is represented as a function of the algorithmic bias γ and the probability of user-media interaction p_m. Values are averaged on 100 independent runs of each setting.

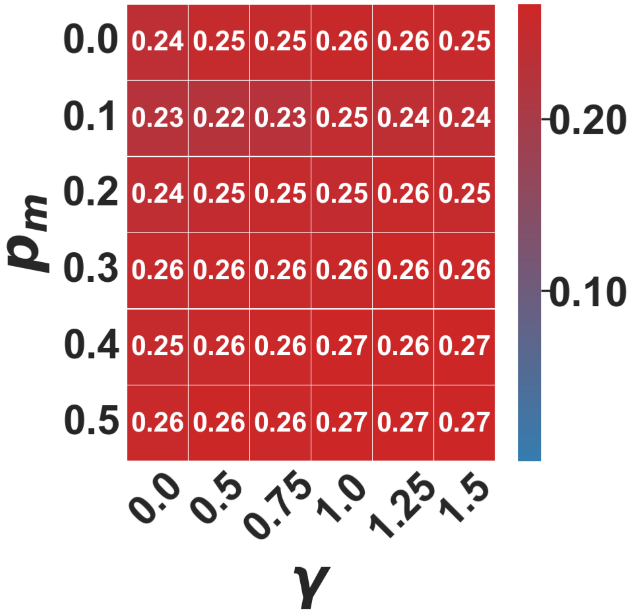

(a) $\varepsilon = 0.2$

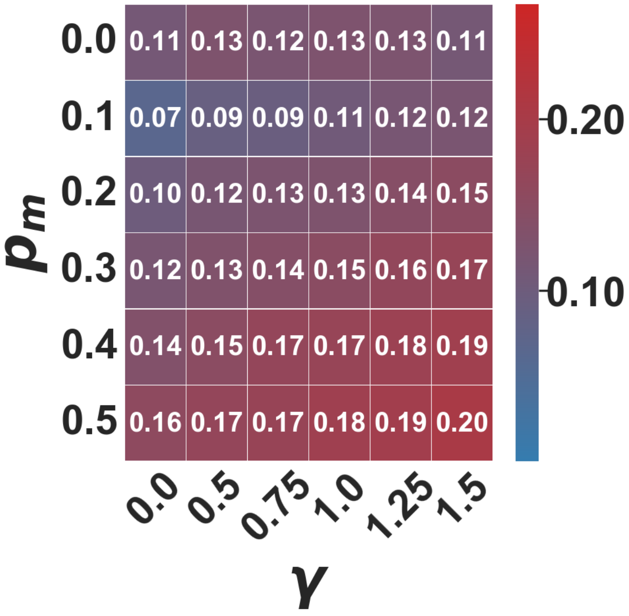

(b) $\varepsilon = 0.3$

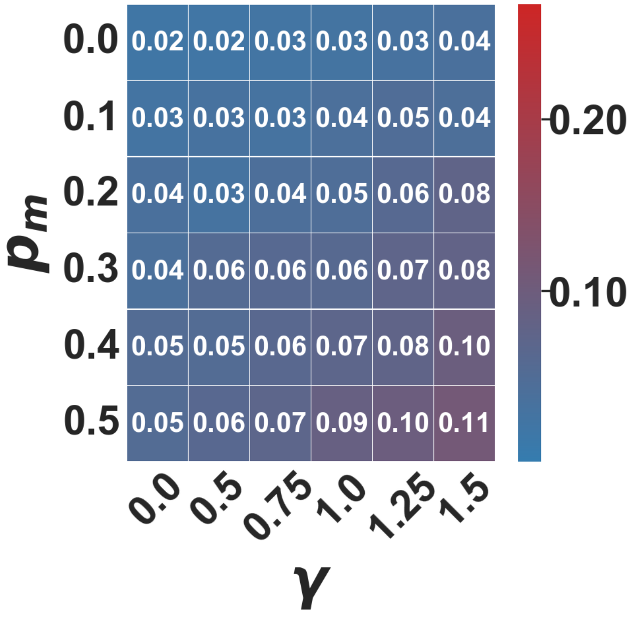

(c) $\varepsilon = 0.4$

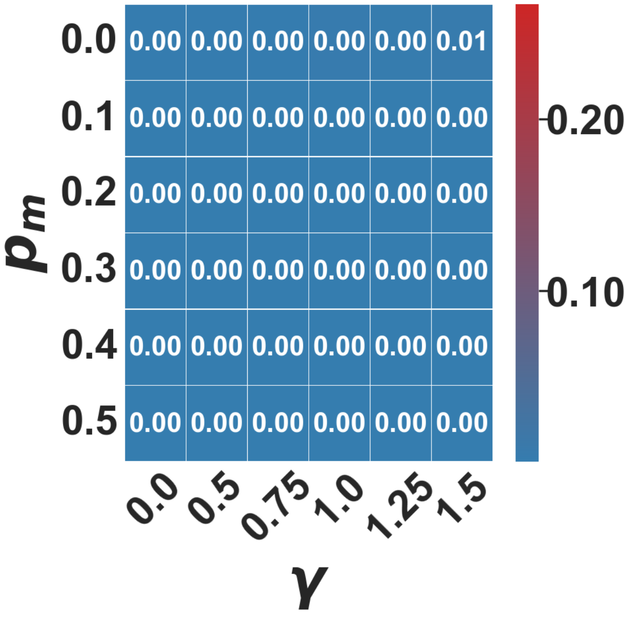

(d) $\varepsilon = 0.5$

Supplementary Figure 5. Average standard deviation of the final opinion distribution in the Algorithmic Bias model with media interactions for different values of ε as a function of γ and p_m in the moderate setting. In the figure the standard deviation of the final opinion distribution is represented as a function of the algorithmic bias γ and the probability of user-media interaction p_m. Values are averaged on 100 independent runs of each setting.

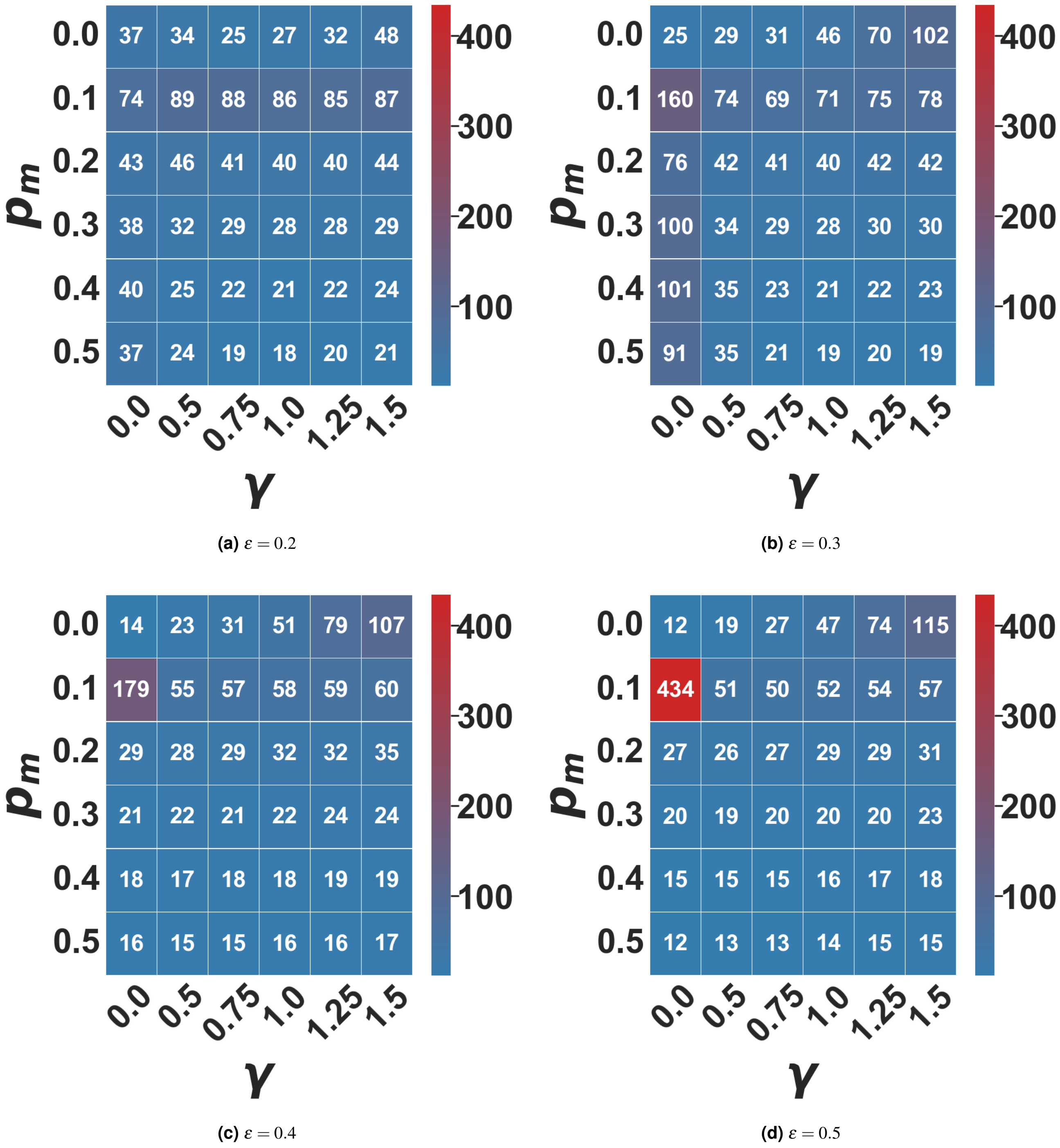

Supplementary Figure 6. Average number of iterations at convergence of the final opinion distribution in the Algorithmic Bias model with media interactions for different values of ε as a function of γ and p_m in the moderate setting. In the figure the number of iterations at convergence is represented as a function of the algorithmic bias γ and the probability of user-media interaction p_m. Values are averaged on 100 independent runs of each setting.

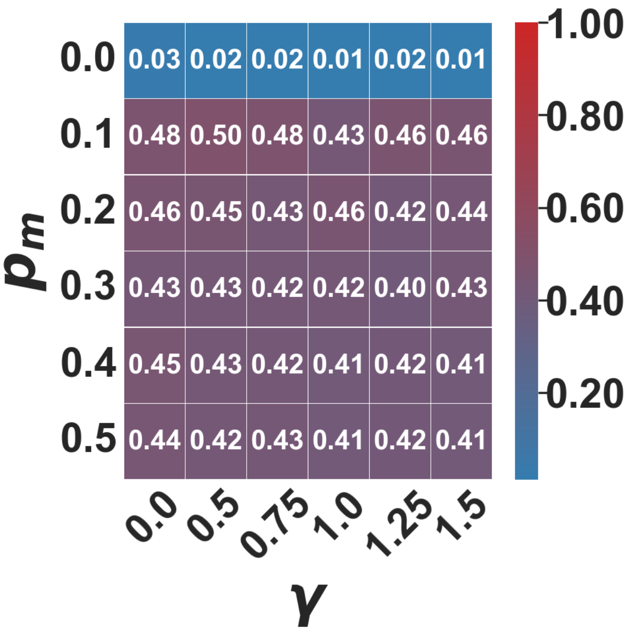

(a) $\varepsilon = 0.2$

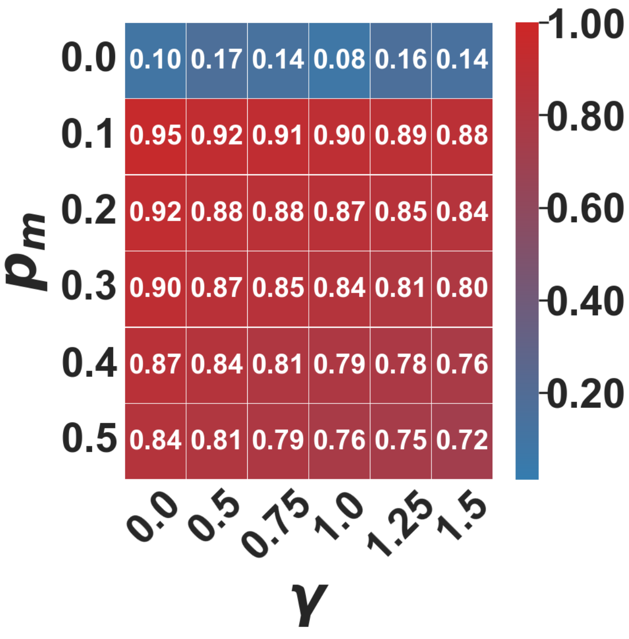

(b) $\varepsilon = 0.3$

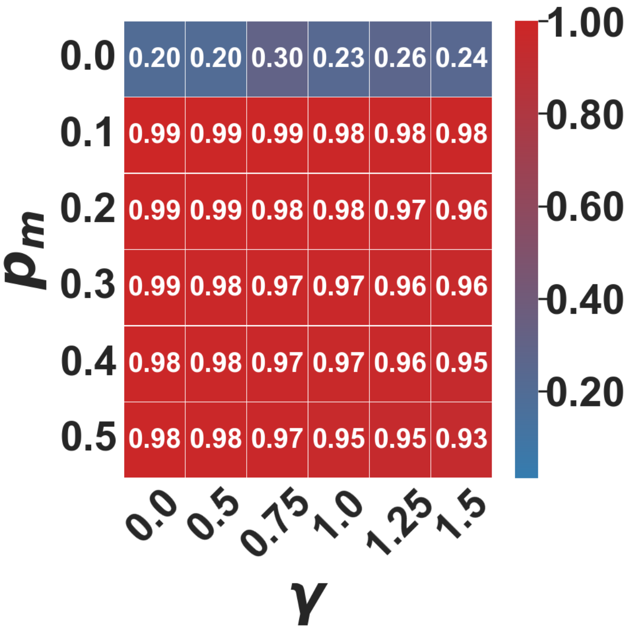

(c) $\varepsilon = 0.4$

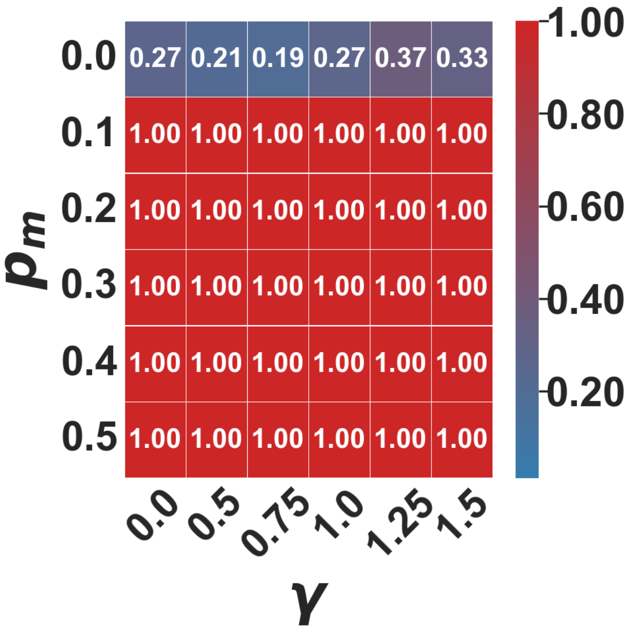

(d) $\varepsilon = 0.5$

Supplementary Figure 7. Average fraction of agents in the moderate cluster in the Algorithmic Bias model with media interactions for different values of ε as a function of γ and p_m in the moderate setting. In the figure the average fraction of agents in the moderate cluster is represented as a function of the algorithmic bias γ and the probability of user-media interaction p_m. Values are averaged on 100 independent runs of each setting.

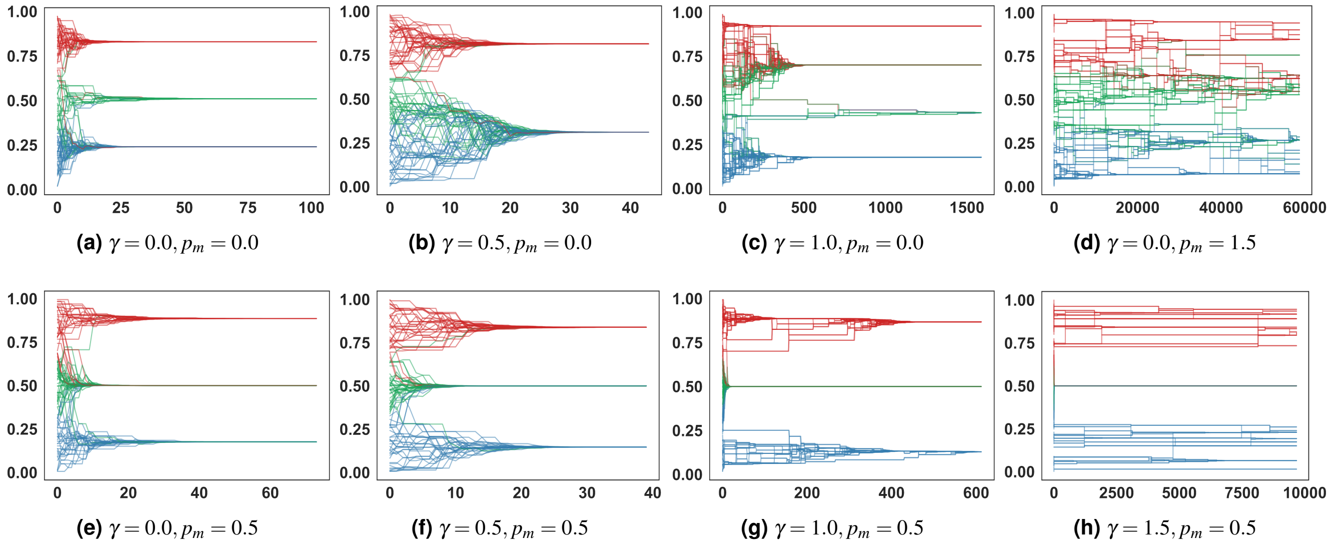

(a) $\gamma = 0.0, p_m = 0.0$ **(b)** $\gamma = 0.5, p_m = 0.0$ **(c)** $\gamma = 1.0, p_m = 0.0$ **(d)** $\gamma = 0.0, p_m = 1.5$

(e) $\gamma = 0.0, p_m = 0.5$ **(f)** $\gamma = 0.5, p_m = 0.5$ **(g)** $\gamma = 1.0, p_m = 0.5$ **(h)** $\gamma = 1.5, p_m = 0.5$

Supplementary Figure 8. Opinion evolution in the Algorithmic Bias model with media interactions in the moderate setting, i.e. with one media with opinion 0.5. Opinion evolution for $\varepsilon = 0.2$.

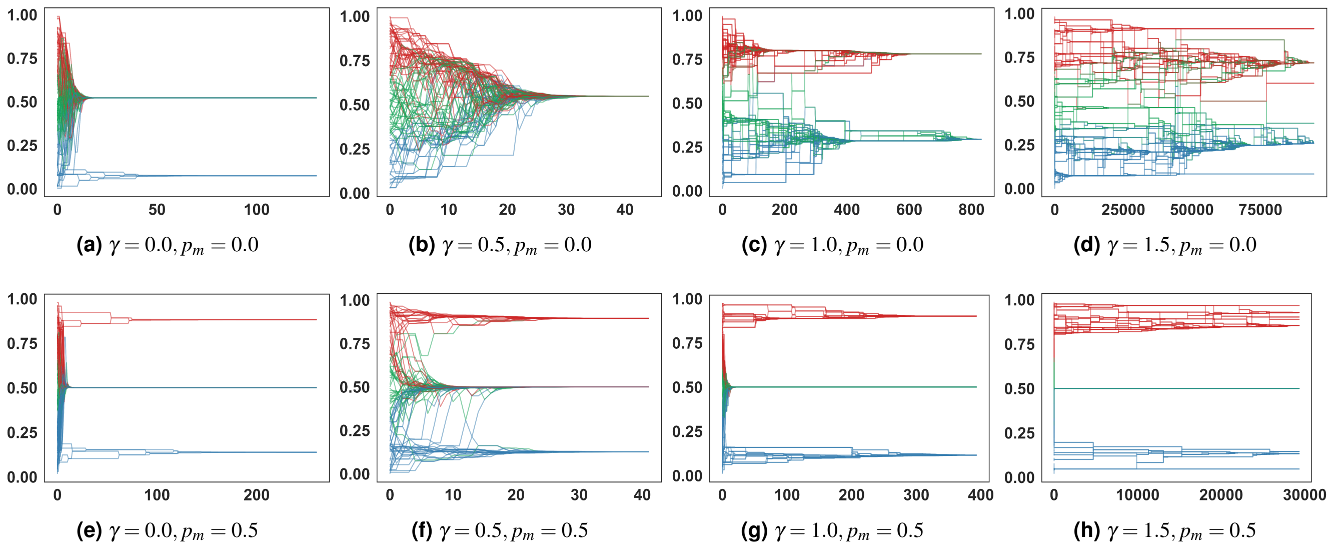

(a) $\gamma = 0.0, p_m = 0.0$ **(b)** $\gamma = 0.5, p_m = 0.0$ **(c)** $\gamma = 1.0, p_m = 0.0$ **(d)** $\gamma = 1.5, p_m = 0.0$

(e) $\gamma = 0.0, p_m = 0.5$ **(f)** $\gamma = 0.5, p_m = 0.5$ **(g)** $\gamma = 1.0, p_m = 0.5$ **(h)** $\gamma = 1.5, p_m = 0.5$

Supplementary Figure 9. Opinion evolution in the Algorithmic Bias model with media interactions in the moderate setting, i.e. with one media with opinion 0.5. Opinion evolution for $\varepsilon = 0.3$.

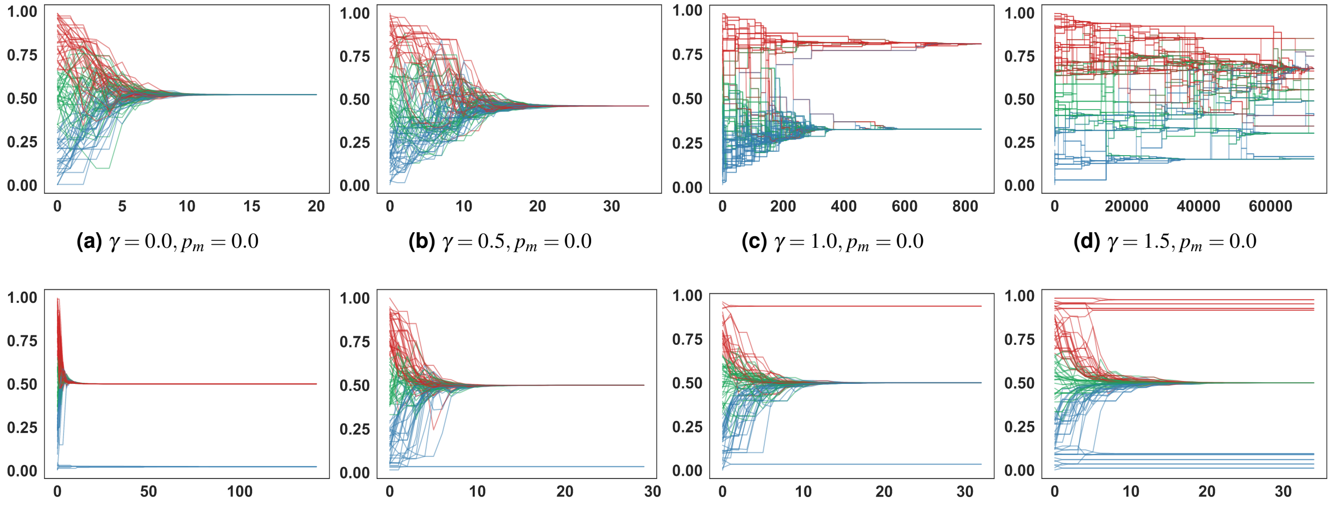

(a) $\gamma = 0.0, p_m = 0.0$ (b) $\gamma = 0.5, p_m = 0.0$ (c) $\gamma = 1.0, p_m = 0.0$ (d) $\gamma = 1.5, p_m = 0.0$

(e) $\gamma = 0.0, p_m = 0.5$ (f) $\gamma = 0.5, p_m = 0.5$ (g) $\gamma = 1.0, p_m = 0.5$ (h) $\gamma = 1.5, p_m = 0.5$

Supplementary Figure 10. Opinion evolution in the Algorithmic Bias model with media interactions in the moderate setting, i.e. with one media with opinion 0.5. Opinion evolution for $\varepsilon = 0.4$.

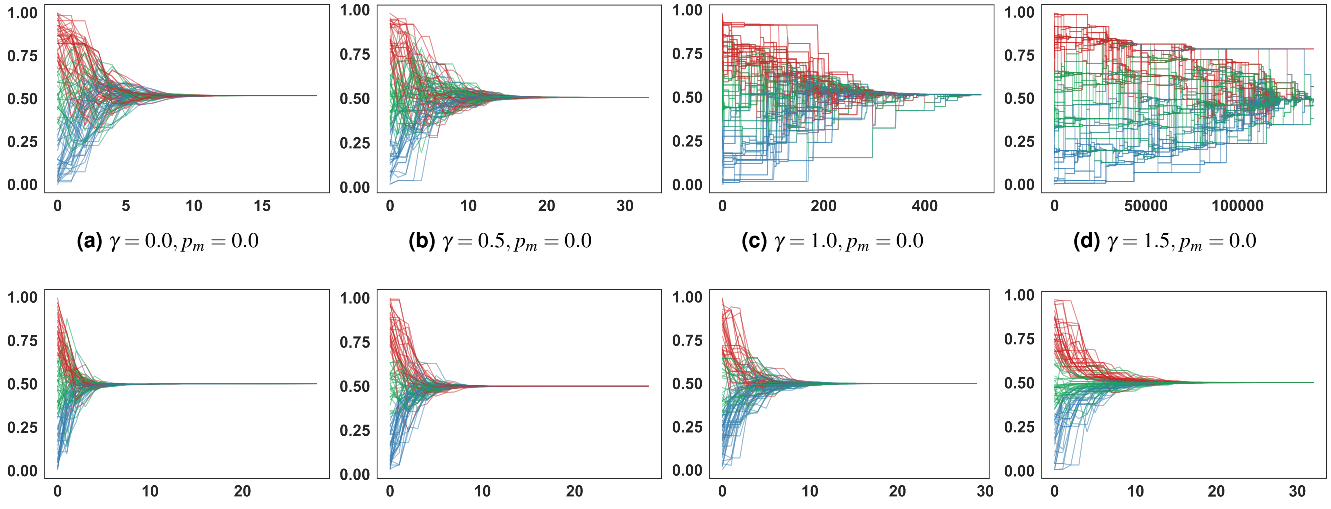

(a) $\gamma = 0.0, p_m = 0.0$ (b) $\gamma = 0.5, p_m = 0.0$ (c) $\gamma = 1.0, p_m = 0.0$ (d) $\gamma = 1.5, p_m = 0.0$

(e) $\gamma = 0.0, p_m = 0.5$ (f) $\gamma = 0.5, p_m = 0.5$ (g) $\gamma = 1.0, p_m = 0.5$ (h) $\gamma = 1.5, p_m = 0.5$

Supplementary Figure 11. Opinion evolution in the Algorithmic Bias model with media interactions in the moderate setting, i.e. with one media with opinion 0.5. Opinion evolution for $\varepsilon = 0.5$.

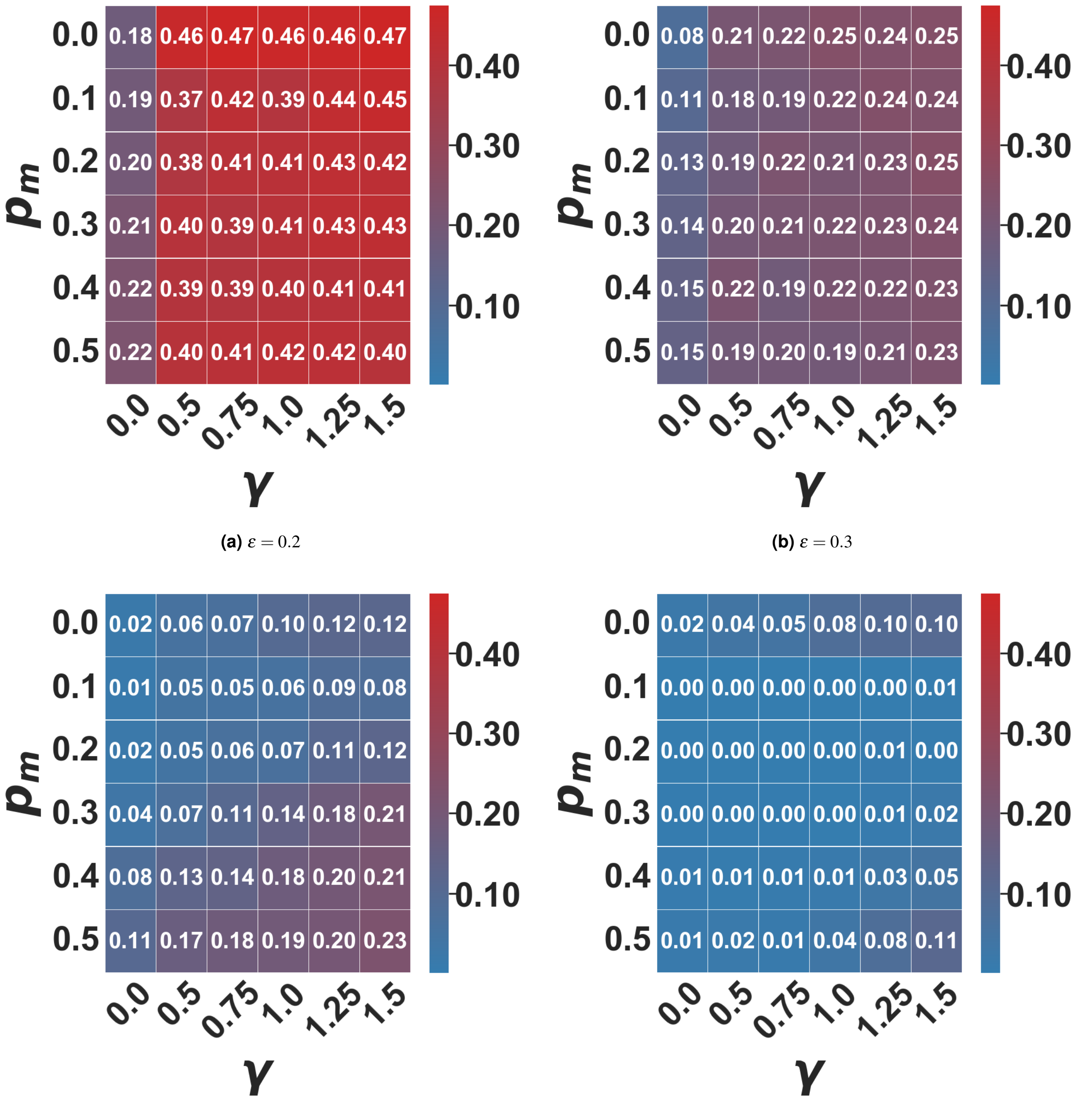

(a) $\varepsilon = 0.2$

(b) $\varepsilon = 0.3$

(c) $\varepsilon = 0.4$

(d) $\varepsilon = 0.5$

Supplementary Figure 12. Average normalised entropy in the Algorithmic Bias model with media interactions for different values of ε as a function of γ and p_m in the extremist setting. In the figure the average values of the entropy of the final opinion distribution are represented as a function of the algorithmic bias γ and the probability of user-media interaction p_m and are normalised in the range $[0,1]$ where 0 means that every agent holds the same opinion and 1 means that final opinions are uniformly distributed. Values are averaged on 100 independent runs of each setting.

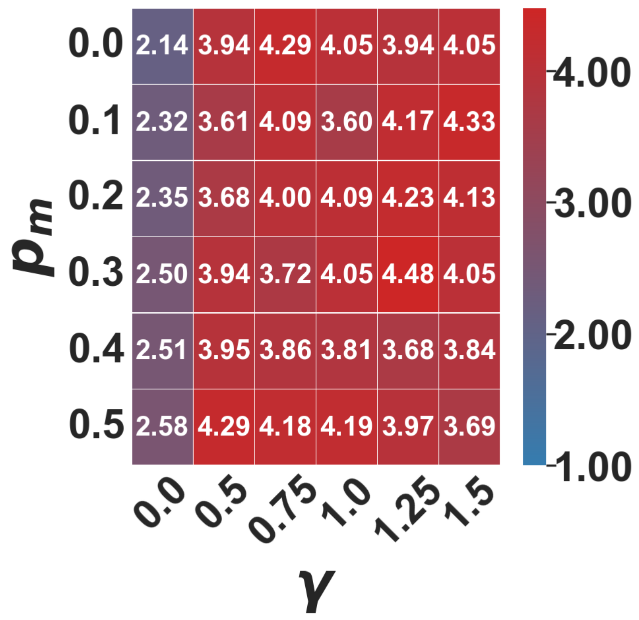

(a) $\varepsilon = 0.2$

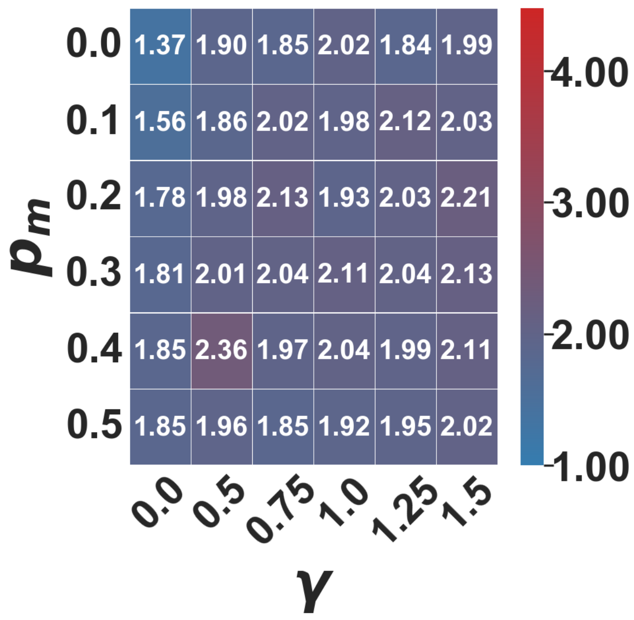

(b) $\varepsilon = 0.3$

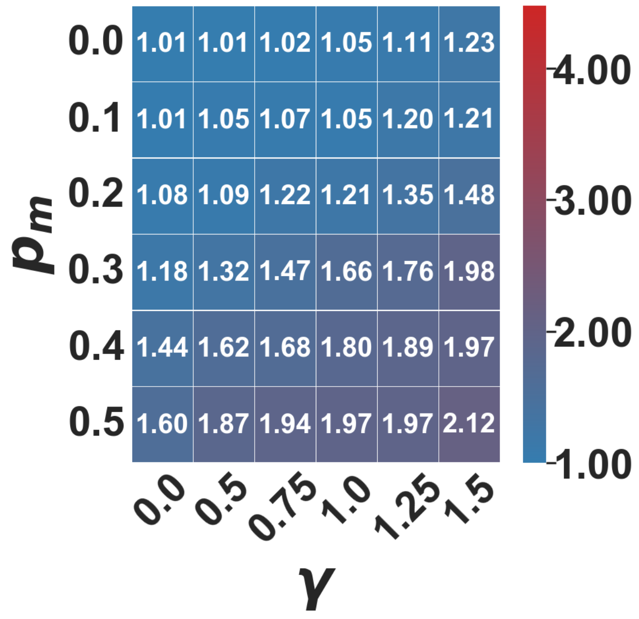

(c) $\varepsilon = 0.4$

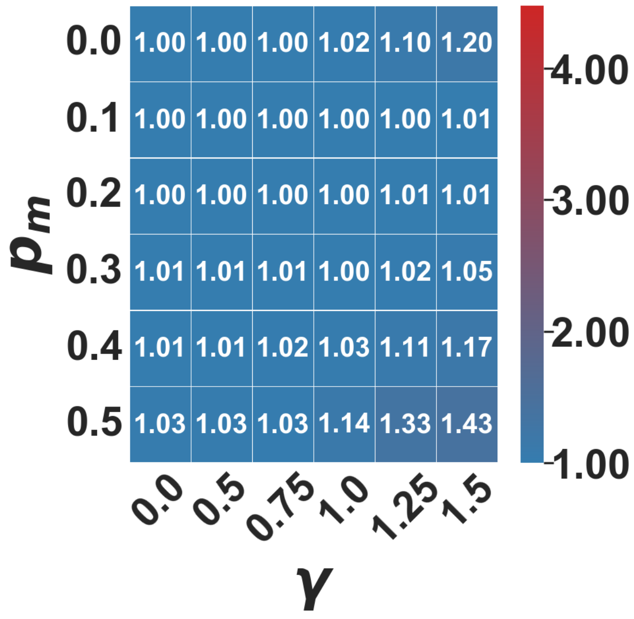

(d) $\varepsilon = 0.5$

Supplementary Figure 13. Average number of clusters in the Algorithmic Bias model with media interactions for different values of ε as a function of γ and p_m in the extremist setting. In the figure the average number of clusters of the final opinion distribution are represented as a function of the algorithmic bias γ and the probability of user-media interaction p_m. Values are averaged on 100 independent runs of each setting.

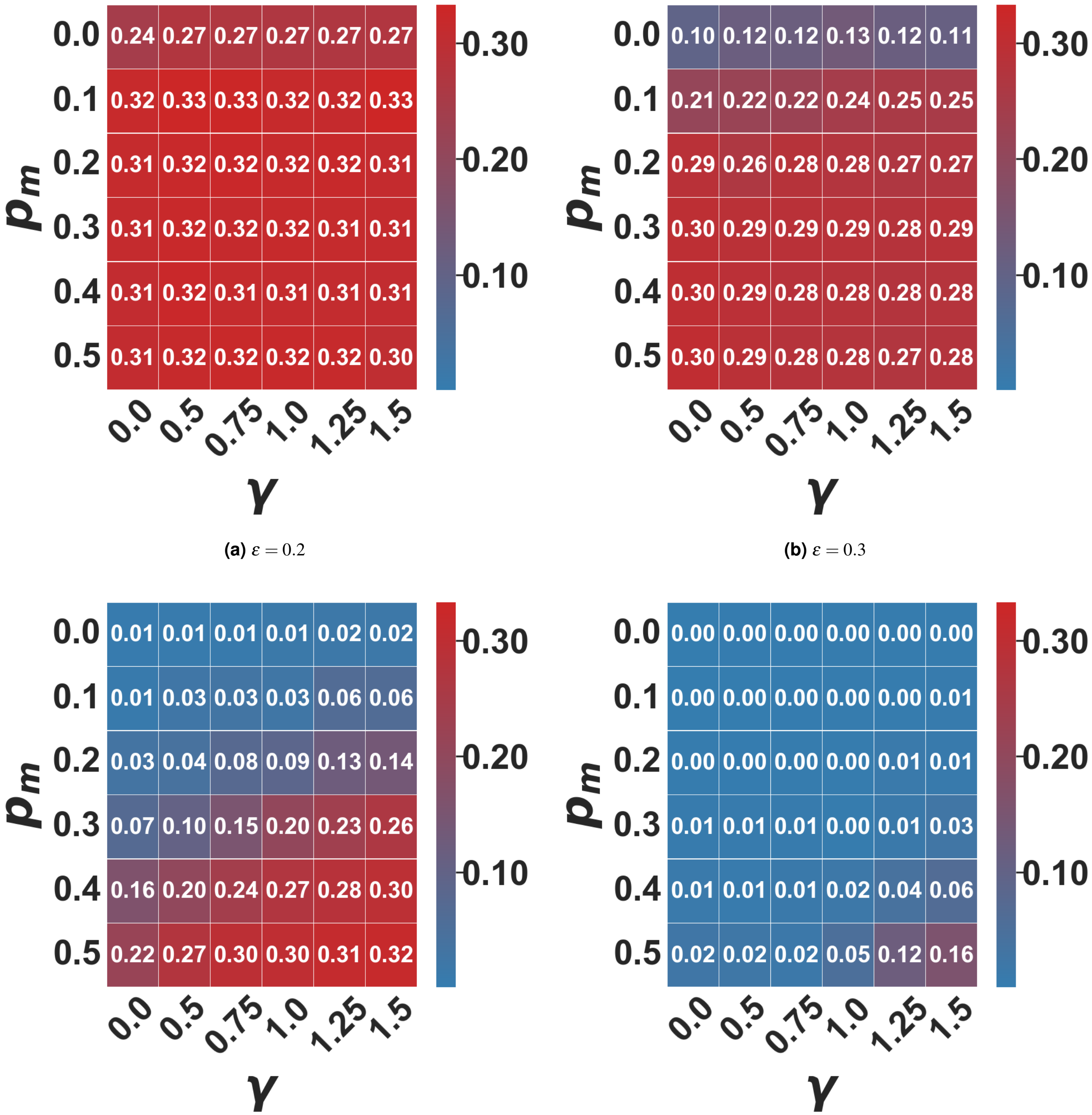

(a) $\varepsilon = 0.2$

(b) $\varepsilon = 0.3$

(c) $\varepsilon = 0.4$

(d) $\varepsilon = 0.5$

Supplementary Figure 14. **Average pairwise opinion distance of the final opinion distribution in the Algorithmic Bias model with media interactions for different values of ε as a function of γ and p_m in the extremist setting.**

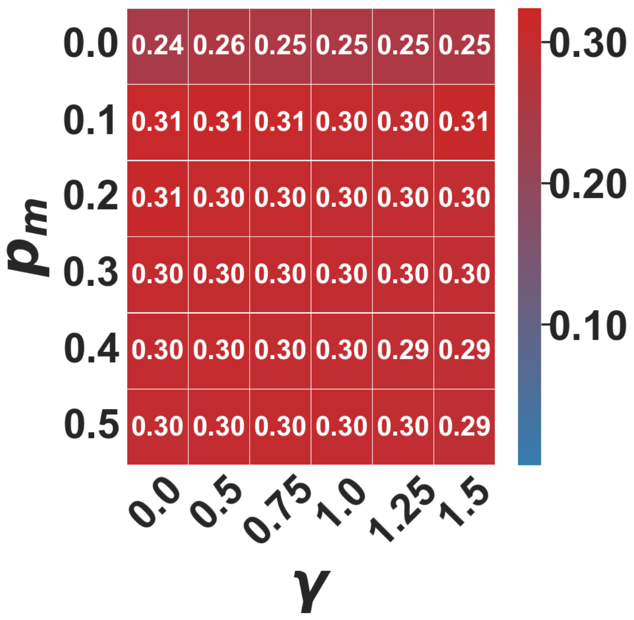

(a) $\varepsilon = 0.2$

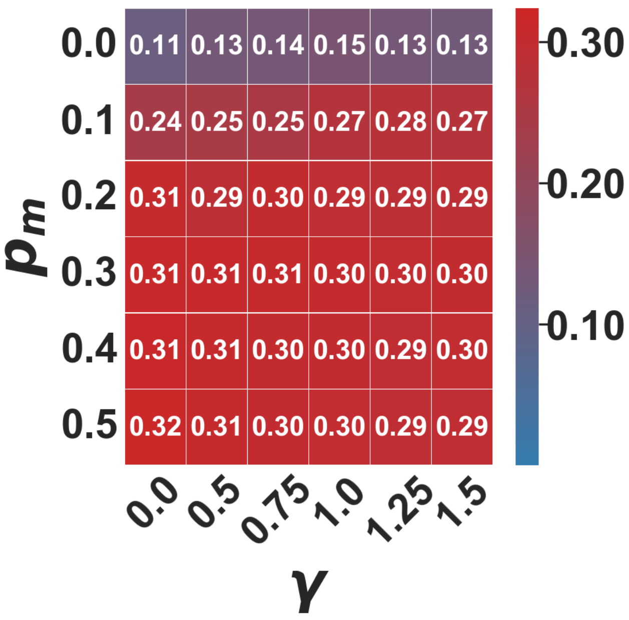

(b) $\varepsilon = 0.3$

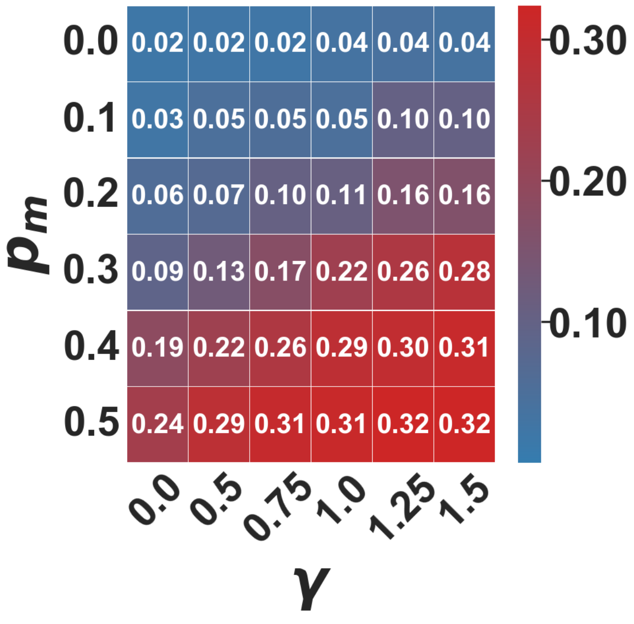

(c) $\varepsilon = 0.4$

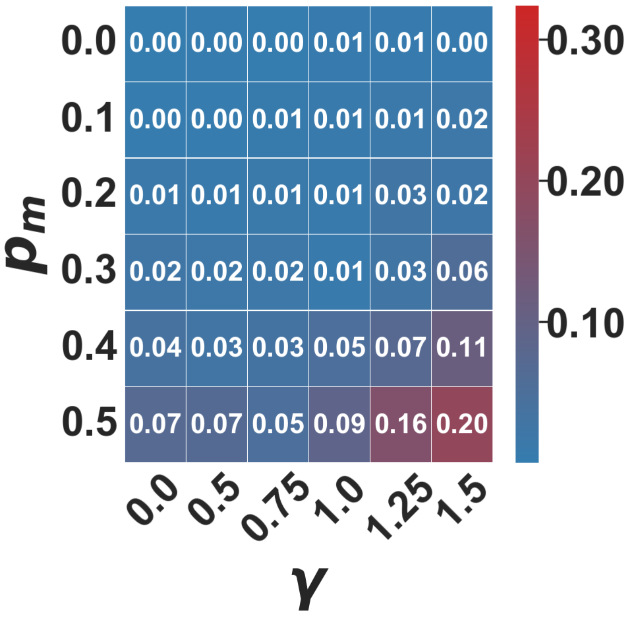

(d) $\varepsilon = 0.5$

Supplementary Figure 15. Average standard deviation of the final opinion distribution in the Algorithmic Bias model with media interactions for different values of ε as a function of γ and p_m in the extremist setting. In the figure the standard deviation of the final opinion distribution is represented as a function of the algorithmic bias γ and the probability of user-media interaction p_m. Values are averaged on 100 independent runs of each setting.

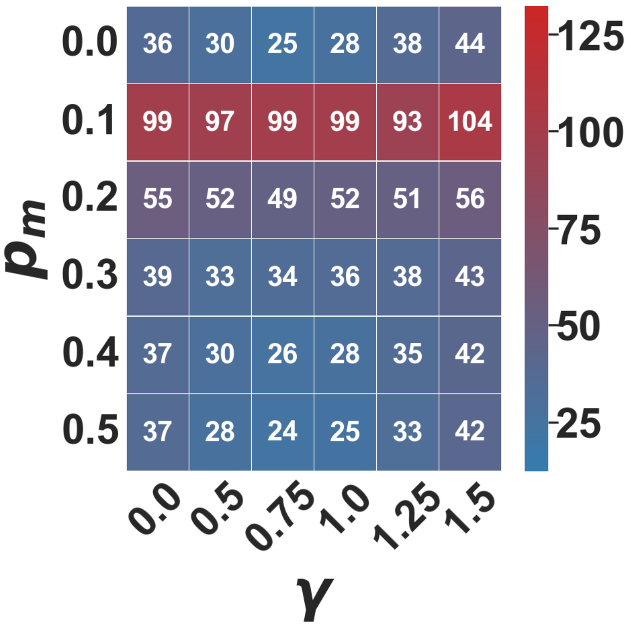

(a) $\varepsilon = 0.2$

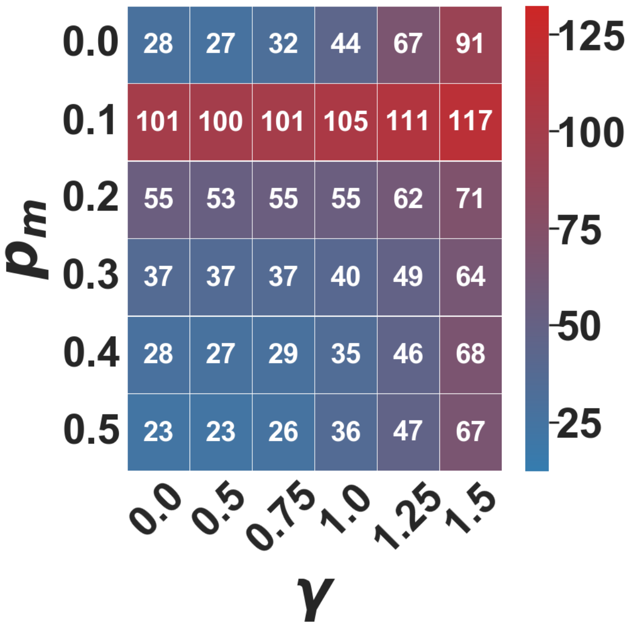

(b) $\varepsilon = 0.3$

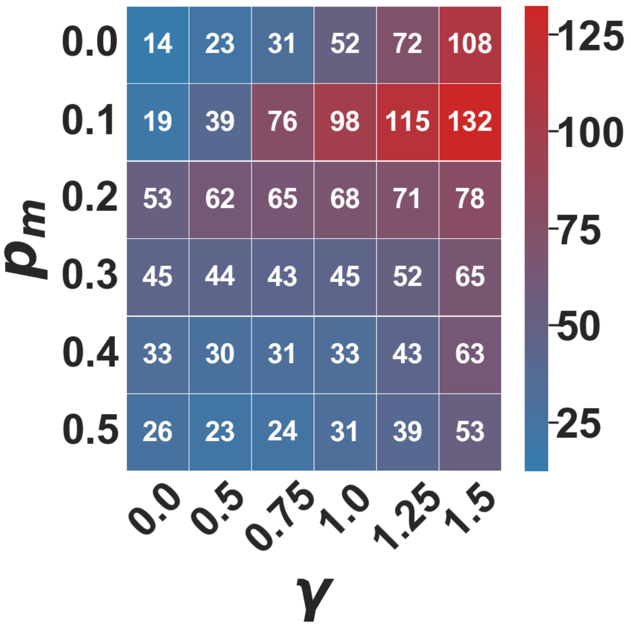

(c) $\varepsilon = 0.4$

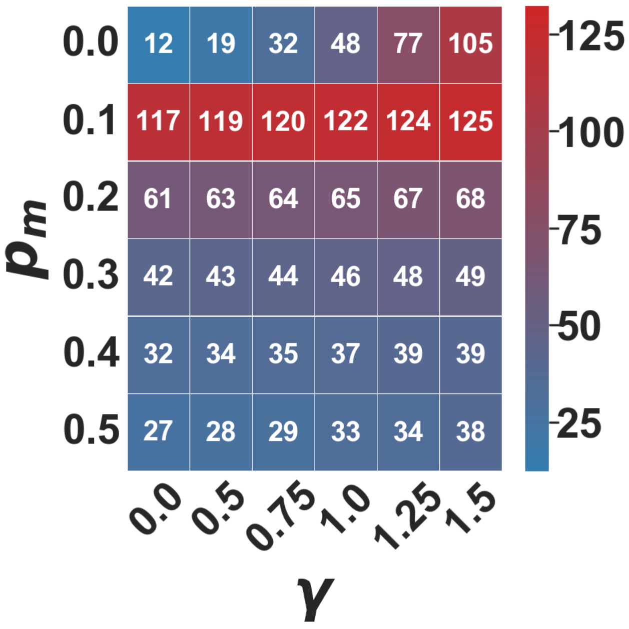

(d) $\varepsilon = 0.5$

Supplementary Figure 16. Average number of iterations at convergence of the final opinion distribution in the Algorithmic Bias model with media interactions for different values of ε as a function of γ and p_m in the extremist setting. In the figure the number of iterations at convergence is represented as a function of the algorithmic bias γ and the probability of user-media interaction p_m. Values are averaged on 100 independent runs of each setting.

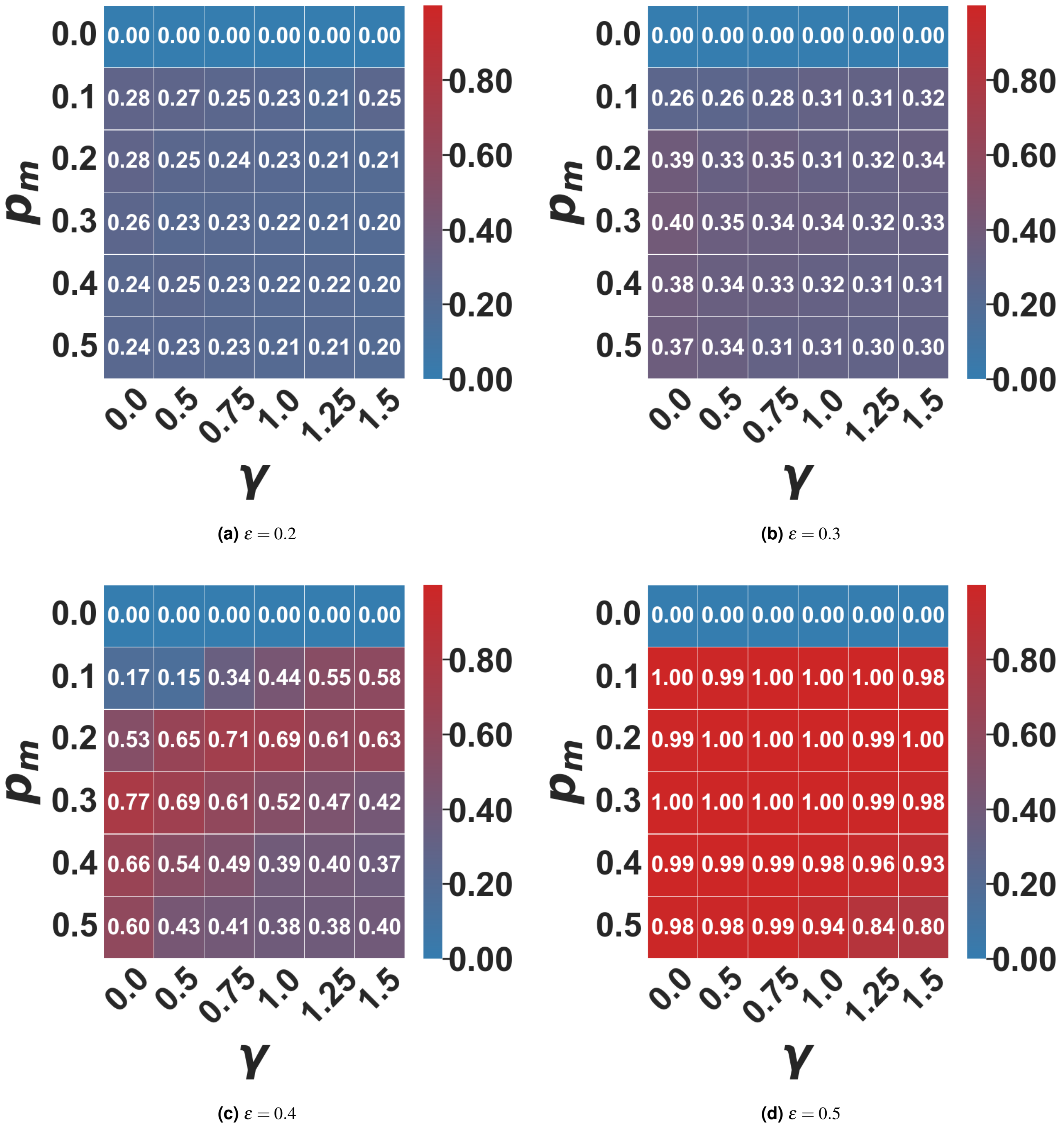

(a) $\varepsilon = 0.2$ (b) $\varepsilon = 0.3$ (c) $\varepsilon = 0.4$ (d) $\varepsilon = 0.5$

Supplementary Figure 17. Average percentage of nodes holding opinion 0.0 at convergence of the final opinion distribution in the Algorithmic Bias model with media interactions for different values of ε as a function of γ and p_m in the extremist setting. In the figure the average percentage of nodes holding opinion 0.0 is represented as a function of the algorithmic bias γ and the probability of user-media interaction p_m. Values are averaged on 100 independent runs of each setting.

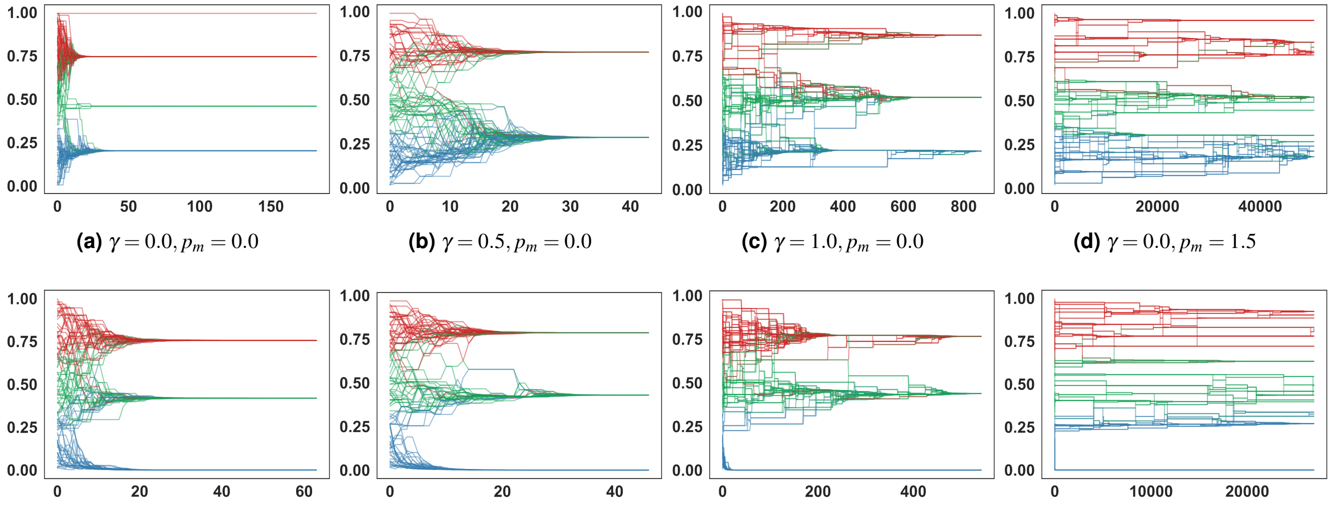

(a) $\gamma = 0.0, p_m = 0.0$ (b) $\gamma = 0.5, p_m = 0.0$ (c) $\gamma = 1.0, p_m = 0.0$ (d) $\gamma = 0.0, p_m = 1.5$

(e) $\gamma = 0.0, p_m = 0.5$ (f) $\gamma = 0.5, p_m = 0.5$ (g) $\gamma = 1.0, p_m = 0.5$ (h) $\gamma = 1.5, p_m = 0.5$

Supplementary Figure 18. Opinion evolution in the Algorithmic Bias model with media interactions in the extremist setting, i.e. with one media with opinion 0.0. Opinion evolution for $\varepsilon = 0.2$.

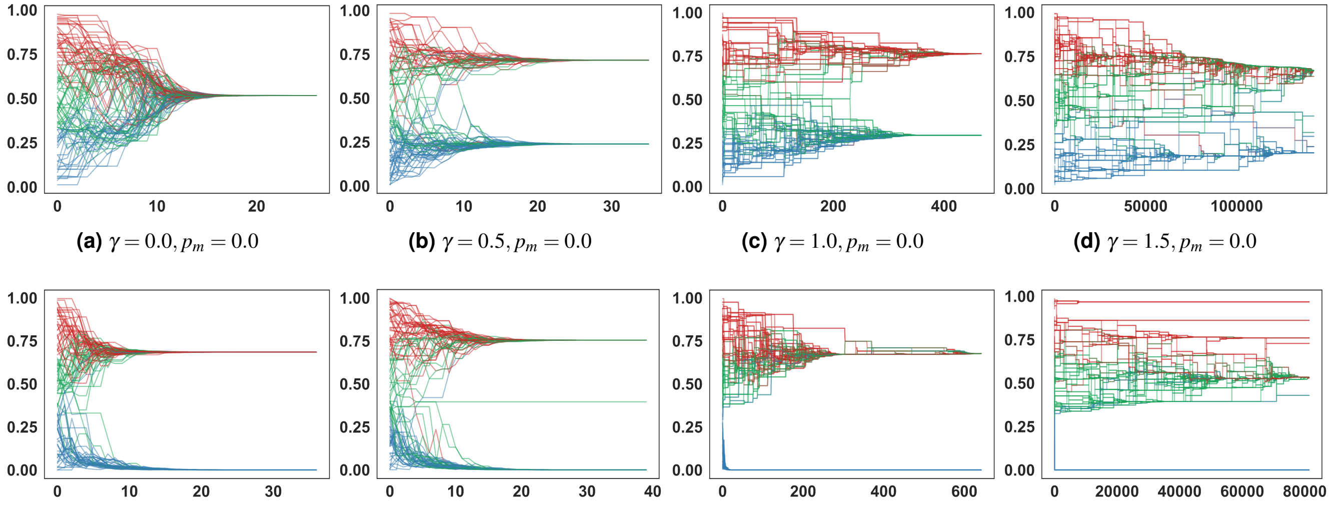

(a) $\gamma = 0.0, p_m = 0.0$ (b) $\gamma = 0.5, p_m = 0.0$ (c) $\gamma = 1.0, p_m = 0.0$ (d) $\gamma = 1.5, p_m = 0.0$

(e) $\gamma = 0.0, p_m = 0.5$ (f) $\gamma = 0.5, p_m = 0.5$ (g) $\gamma = 1.0, p_m = 0.5$ (h) $\gamma = 1.5, p_m = 0.5$

Supplementary Figure 19. Opinion evolution in the Algorithmic Bias model with media interactions in the extremist setting, i.e. with one media with opinion 0.0. Opinion evolution for $\varepsilon = 0.3$.

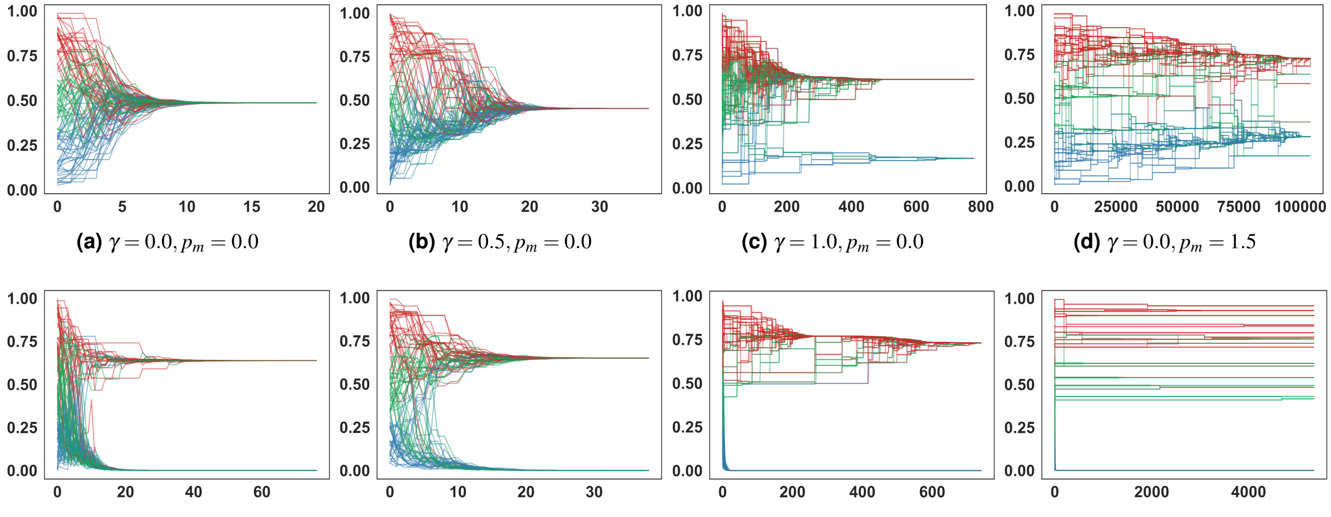

(a) $\gamma = 0.0, p_m = 0.0$ **(b)** $\gamma = 0.5, p_m = 0.0$ **(c)** $\gamma = 1.0, p_m = 0.0$ **(d)** $\gamma = 0.0, p_m = 1.5$

(e) $\gamma = 0.0, p_m = 0.5$ **(f)** $\gamma = 0.5, p_m = 0.5$ **(g)** $\gamma = 1.0, p_m = 0.5$ **(h)** $\gamma = 1.5, p_m = 0.5$

Supplementary Figure 20. Opinion evolution in the Algorithmic Bias model with media interactions in the extremist setting, i.e. with one media with opinion 0.0. Opinion evolution for $\varepsilon = 0.4$.

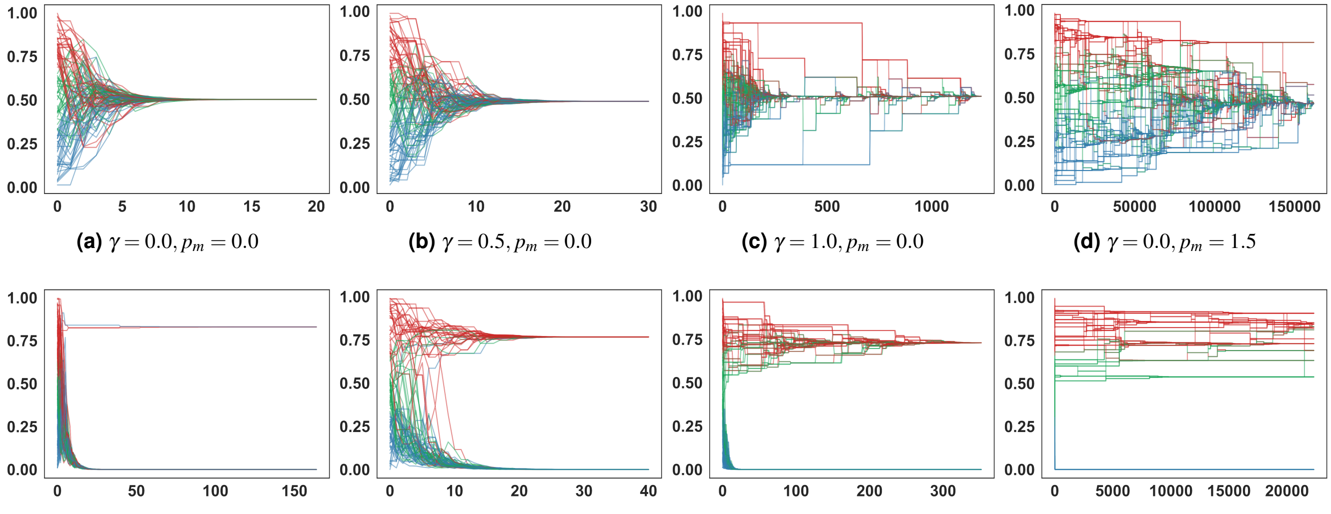

(a) $\gamma = 0.0, p_m = 0.0$ **(b)** $\gamma = 0.5, p_m = 0.0$ **(c)** $\gamma = 1.0, p_m = 0.0$ **(d)** $\gamma = 0.0, p_m = 1.5$

(e) $\gamma = 0.0, p_m = 0.5$ **(f)** $\gamma = 0.5, p_m = 0.5$ **(g)** $\gamma = 1.0, p_m = 0.5$ **(h)** $\gamma = 1.5, p_m = 0.5$

Supplementary Figure 21. Opinion evolution in the Algorithmic Bias model with media interactions in the extremist setting, i.e. with one media with opinion 0.0. Opinion evolution for $\varepsilon = 0.5$.

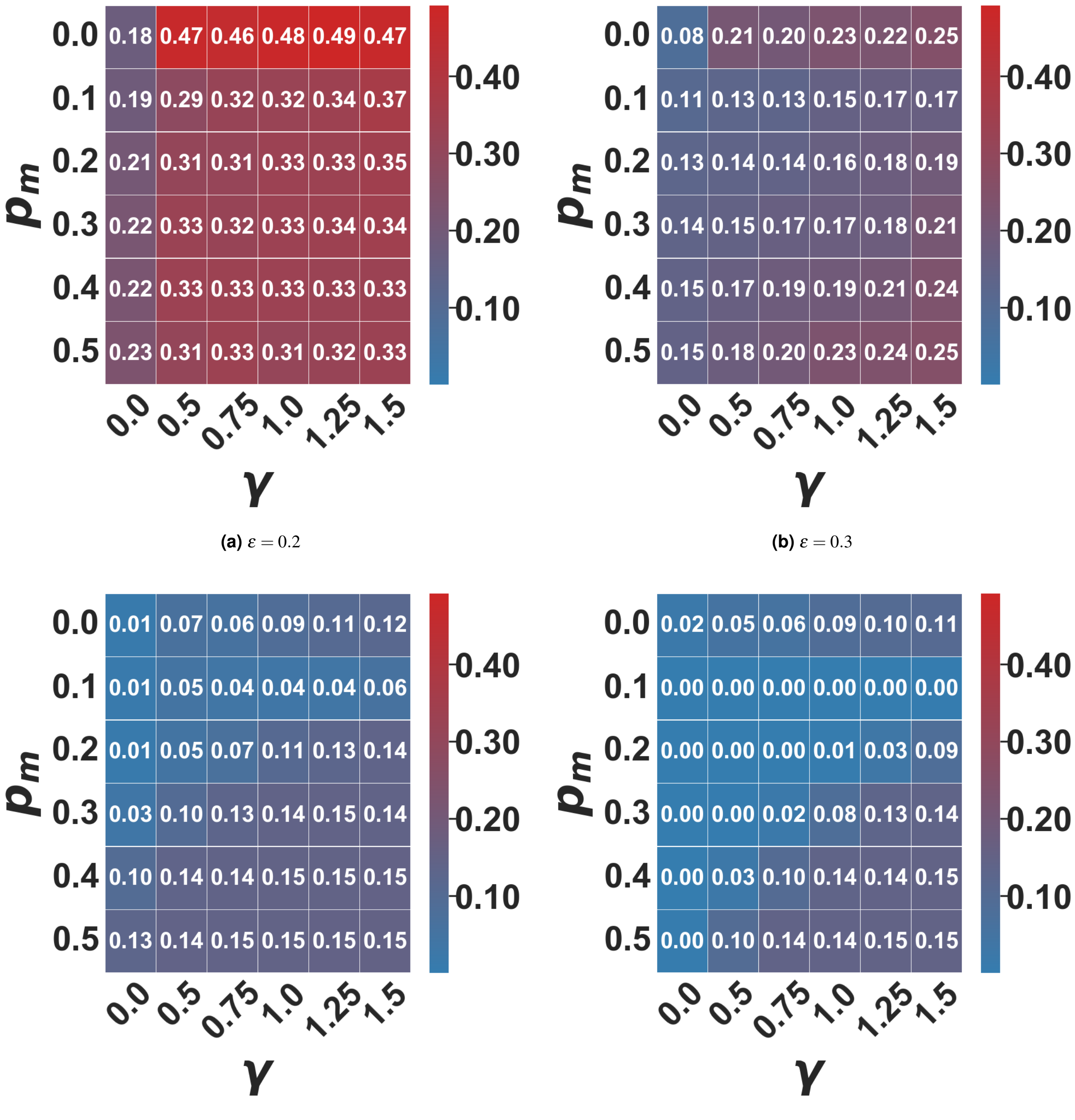

(a) $\varepsilon = 0.2$

(b) $\varepsilon = 0.3$

(c) $\varepsilon = 0.4$

(d) $\varepsilon = 0.5$

Supplementary Figure 22. Average normalised entropy in the Algorithmic Bias model with media interactions for different values of ε as a function of γ and p_m in the polarised setting. In the figure the average values of the entropy of the final opinion distribution are represented as a function of the algorithmic bias γ and the probability of user-media interaction p_m and are normalised in the range $[0,1]$ where 0 means that every agent holds the same opinion and 1 means that final opinions are uniformly distributed. Values are averaged on 100 independent runs of each setting.

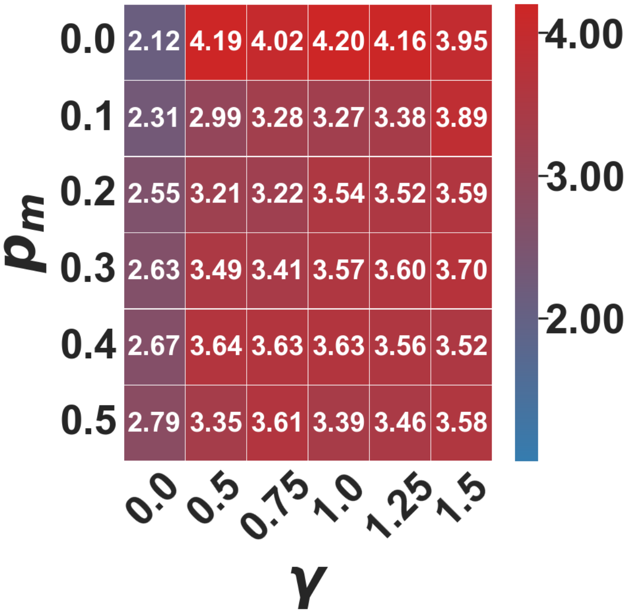

(a) $\varepsilon = 0.2$

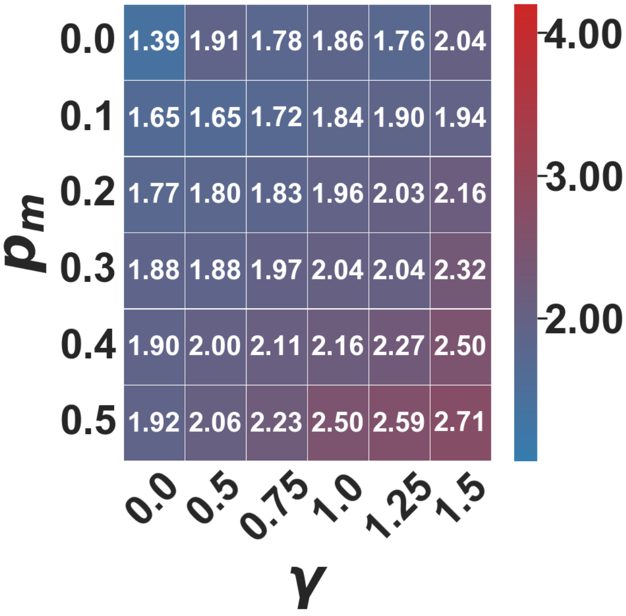

(b) $\varepsilon = 0.3$

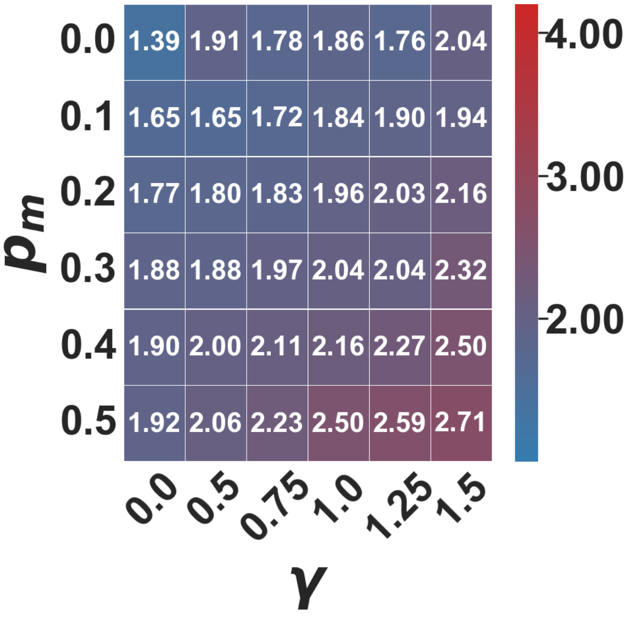

(c) $\varepsilon = 0.4$

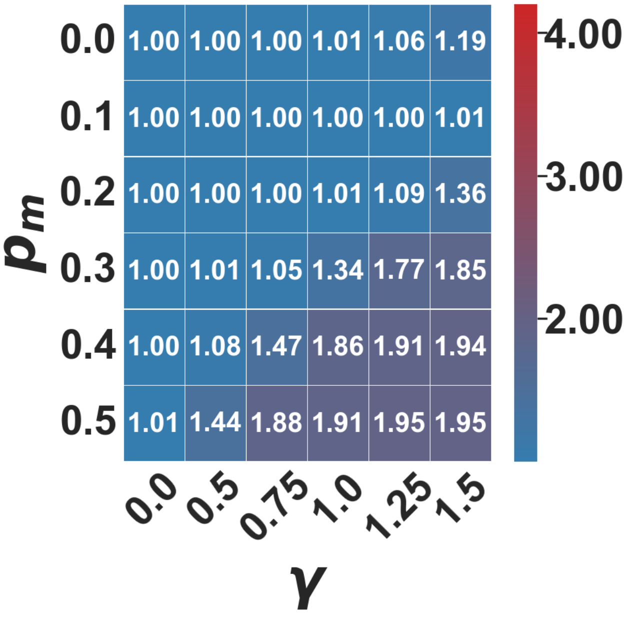

(d) $\varepsilon = 0.5$

Supplementary Figure 23. Average number of clusters in the Algorithmic Bias model with media interactions for different values of ε as a function of γ and p_m in the polarised setting. In the figure the average number of clusters of the final opinion distribution are represented as a function of the algorithmic bias γ and the probability of user-media interaction p_m. Values are averaged on 100 independent runs of each setting.

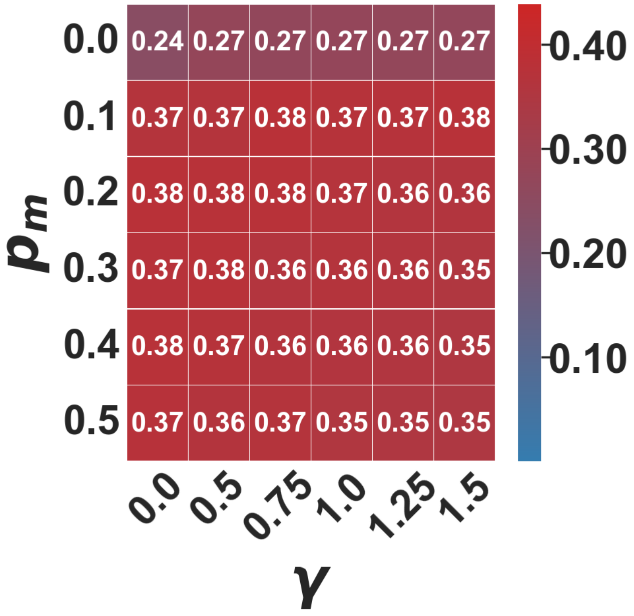

(a) $\varepsilon = 0.2$

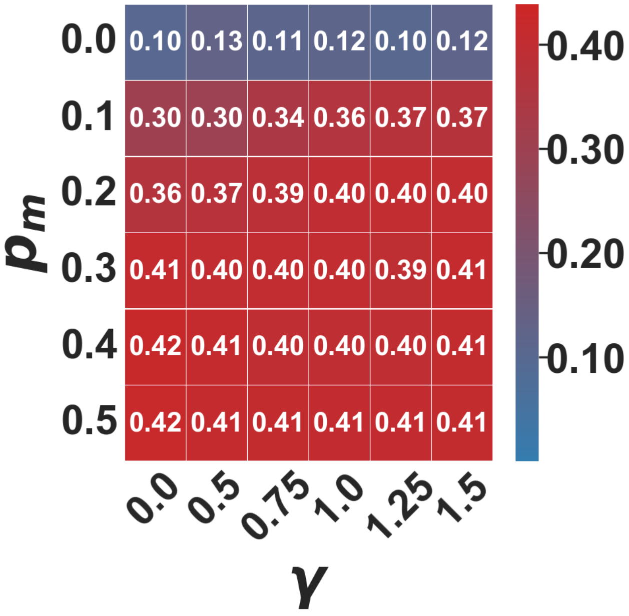

(b) $\varepsilon = 0.3$

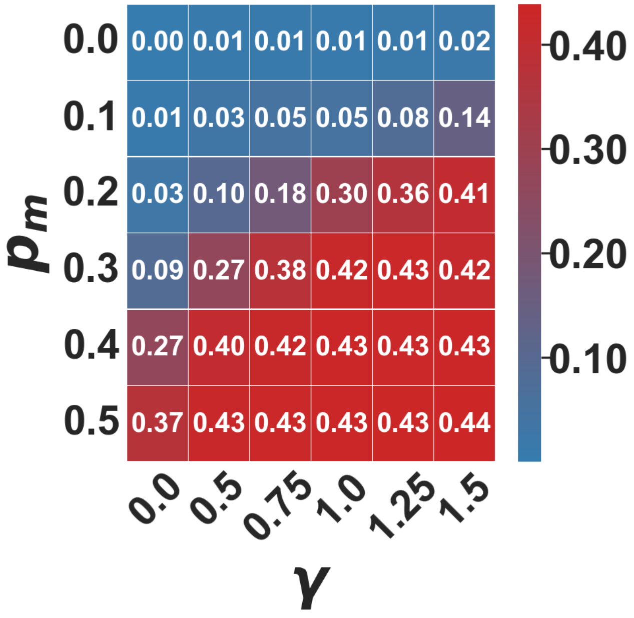

(c) $\varepsilon = 0.4$

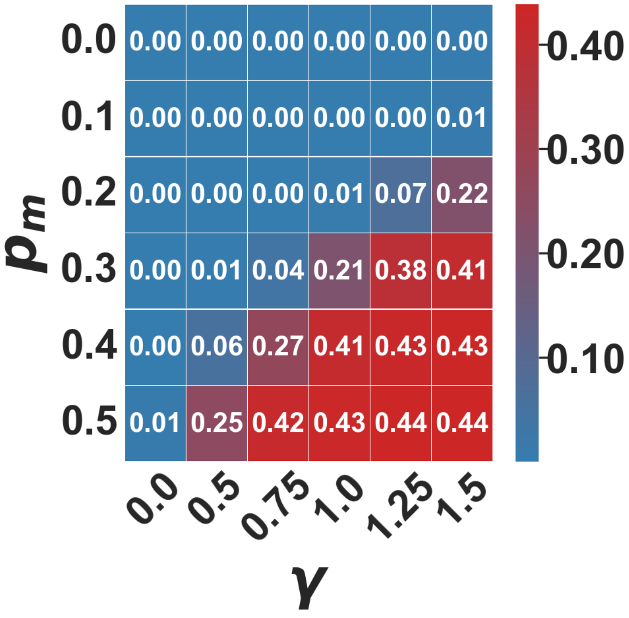

(d) $\varepsilon = 0.5$

Supplementary Figure 24. Average pairwise opinion distance of the final opinion distribution in the Algorithmic Bias model with media interactions for different values of ε as a function of γ and p_m in the polarised setting. In the figure the average pairwise opinion distance of the final opinion distribution is represented as a function of the algorithmic bias γ and the probability of user-media interaction p_m. Values are averaged on 100 independent runs of each setting.

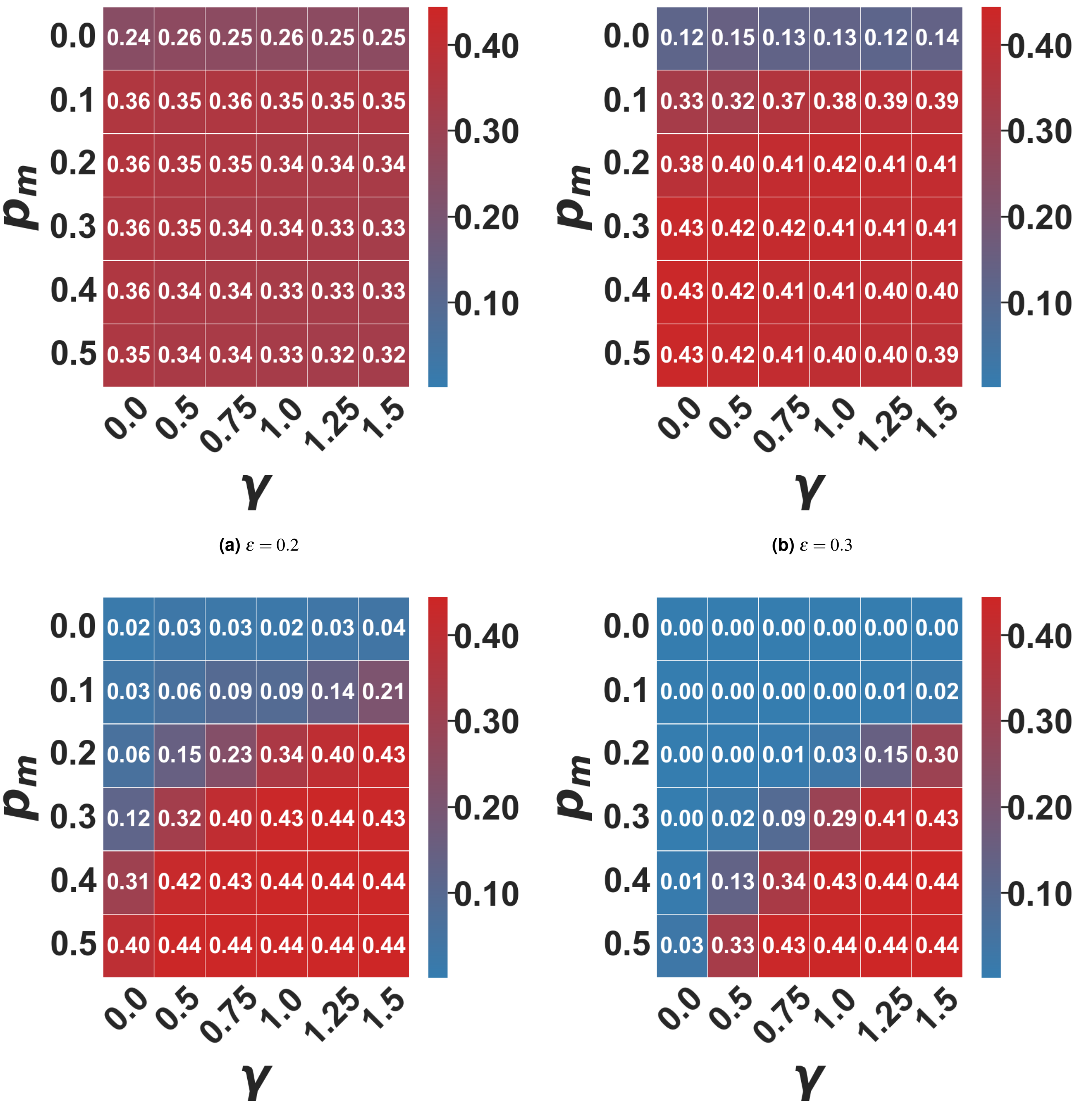

(a) $\varepsilon = 0.2$ **(b)** $\varepsilon = 0.3$ **(c)** $\varepsilon = 0.4$ **(d)** $\varepsilon = 0.5$

Supplementary Figure 25. Average standard deviation of the final opinion distribution in the Algorithmic Bias model with media interactions for different values of ε as a function of γ and p_m in the polarised setting. In the figure the standard deviation of the final opinion distribution is represented as a function of the algorithmic bias γ and the probability of user-media interaction p_m. Values are averaged on 100 independent runs of each setting.

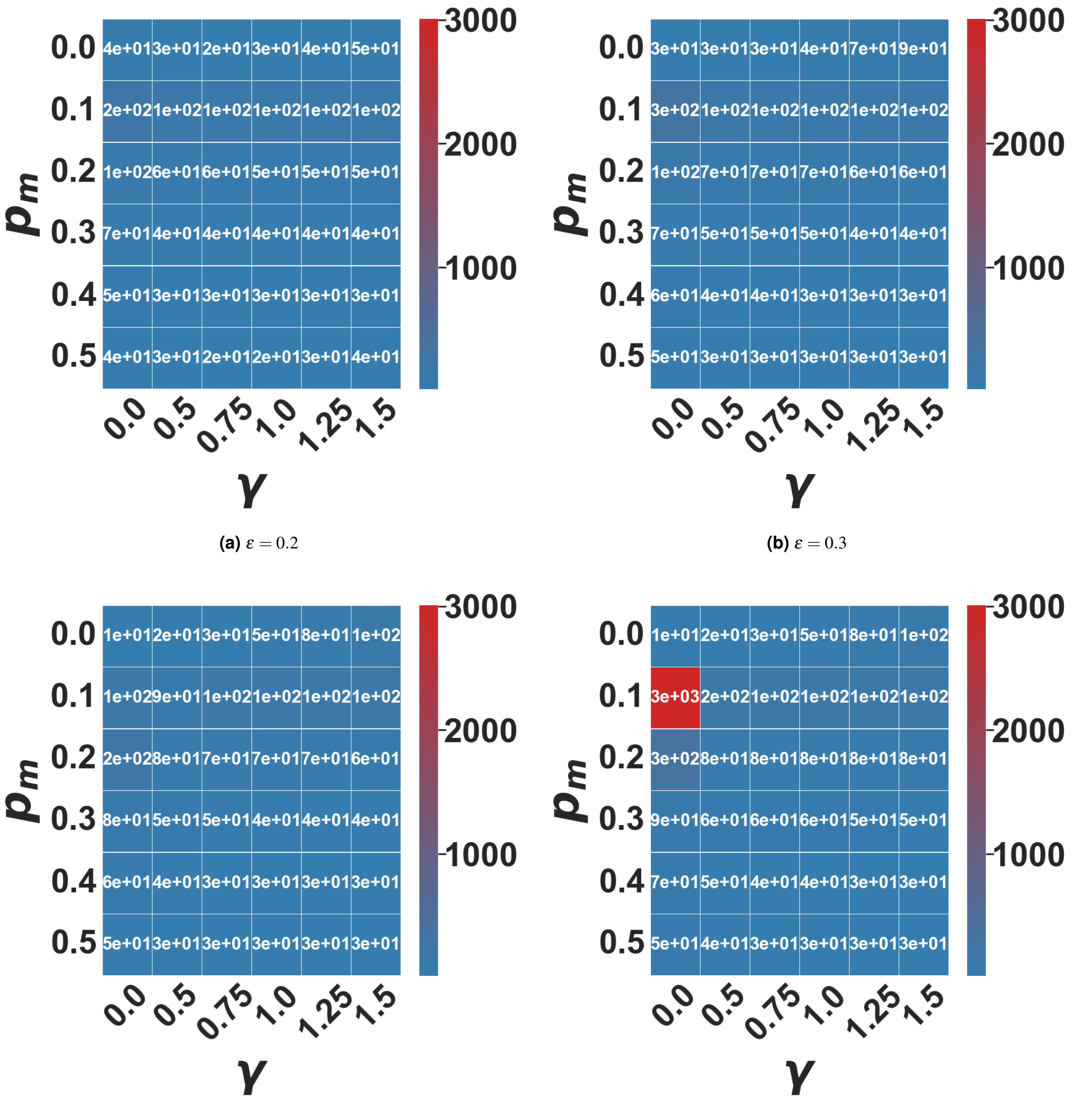

(a) $\varepsilon = 0.2$ **(b)** $\varepsilon = 0.3$

(c) $\varepsilon = 0.4$ **(d)** $\varepsilon = 0.5$

Supplementary Figure 26. Average number of iterations at convergence of the final opinion distribution in the Algorithmic Bias model with media interactions for different values of ε as a function of γ and p_m in the polarised setting. In the figure the number of iterations at convergence is represented as a function of the algorithmic bias γ and the probability of user-media interaction p_m. Values are averaged on 100 independent runs of each setting.

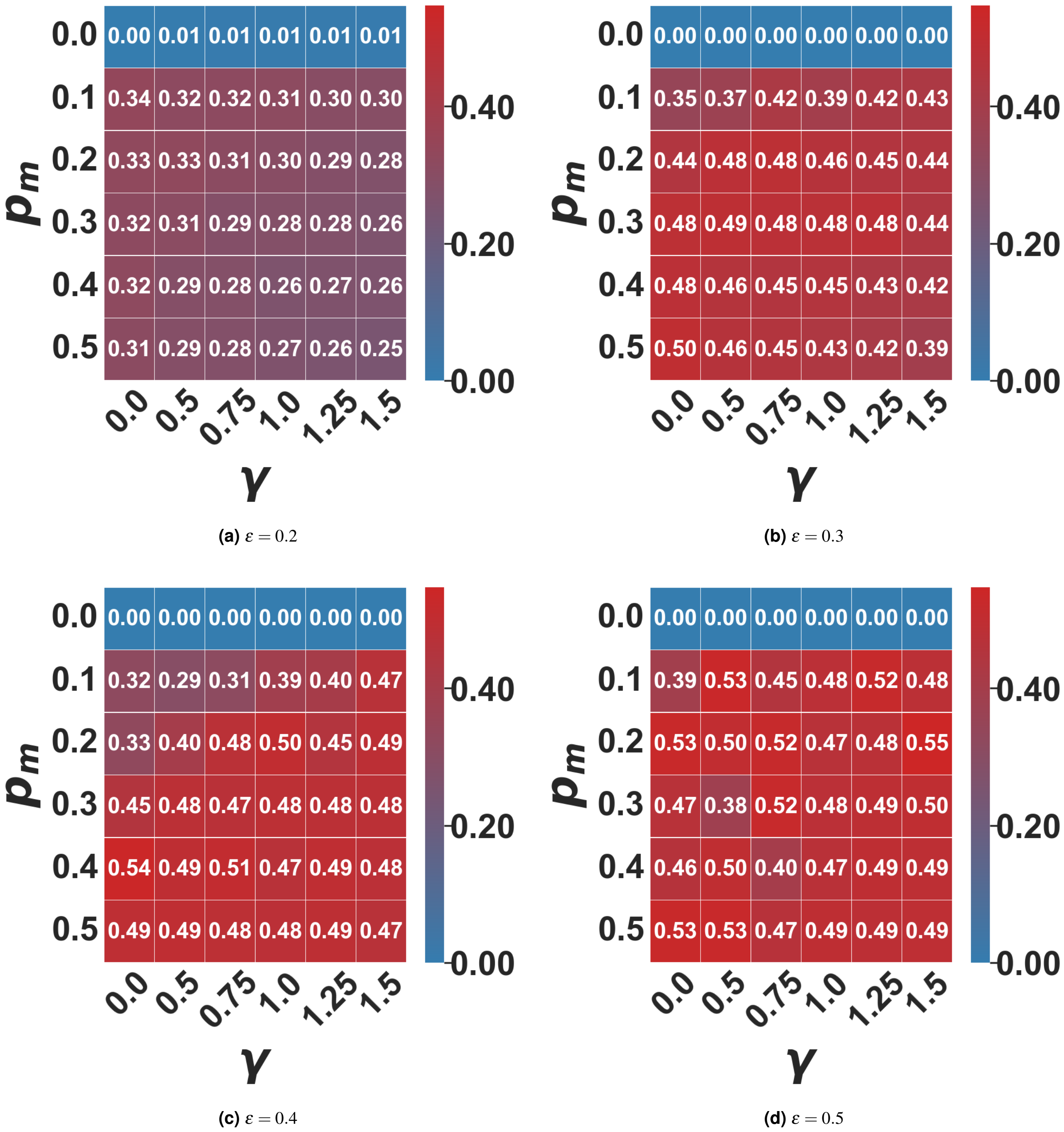

Supplementary Figure 27. Average percentage of nodes holding opinion 0.05 at convergence of the final opinion distribution in the Algorithmic Bias model with media interactions for different values of ε as a function of γ and p_m in the polarised setting. In the figure the average percentage of nodes holding opinion 0.05 is represented as a function of the algorithmic bias γ and the probability of user-media interaction p_m. Values are averaged on 100 independent runs of each setting.

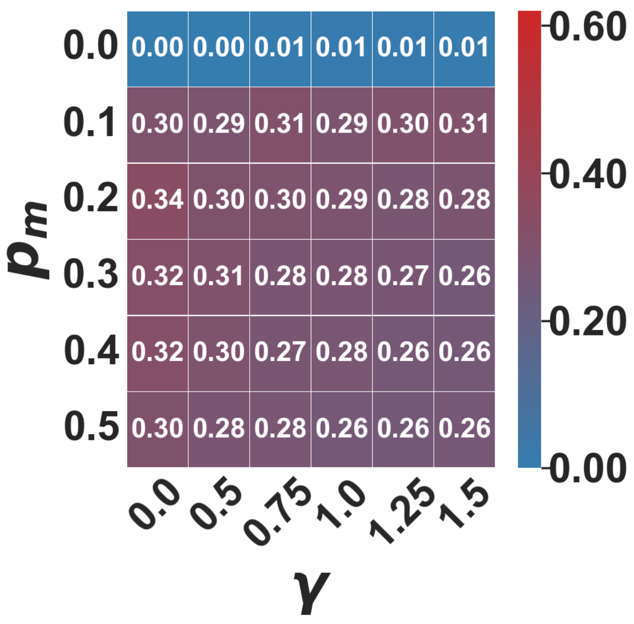

(a) $\varepsilon = 0.2$

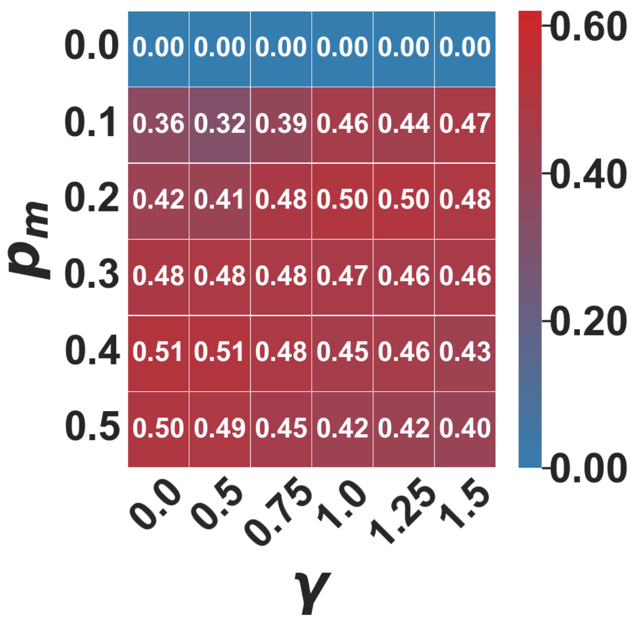

(b) $\varepsilon = 0.3$

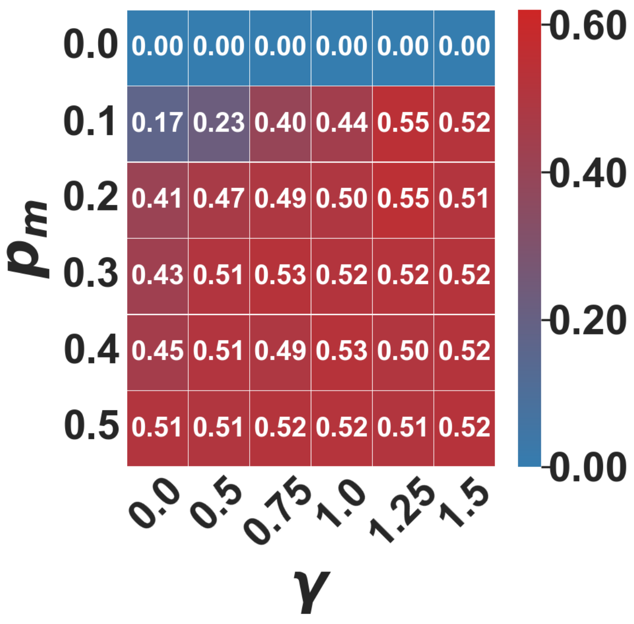

(c) $\varepsilon = 0.4$

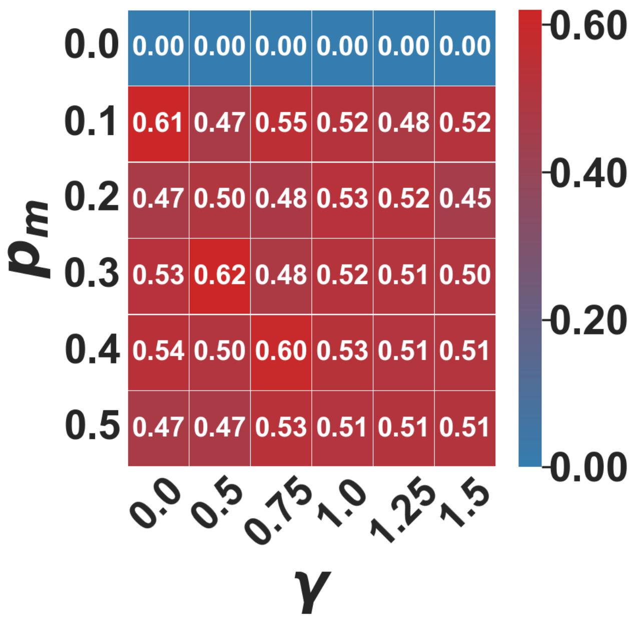

(d) $\varepsilon = 0.5$

Supplementary Figure 28. Average percentage of nodes holding opinion 0.95 at convergence of the final opinion distribution in the Algorithmic Bias model with media interactions for different values of ε as a function of γ and p_m in the polarised setting. In the figure the average percentage of nodes holding opinion 0.95 is represented as a function of the algorithmic bias γ and the probability of user-media interaction p_m. Values are averaged on 100 independent runs of each setting.

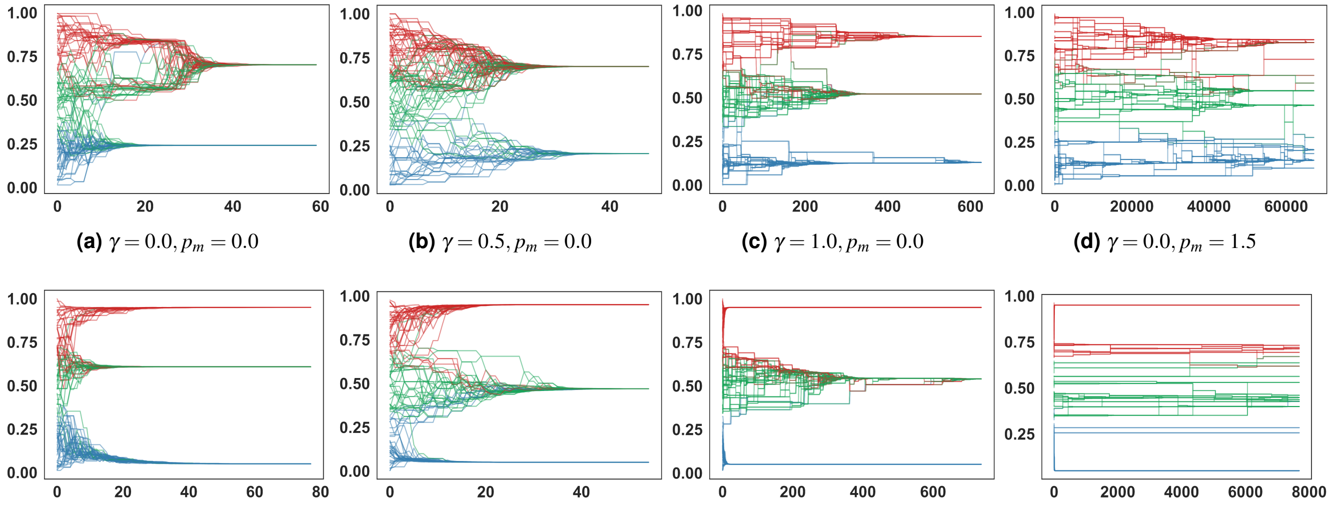

(a) $\gamma = 0.0, p_m = 0.0$ **(b)** $\gamma = 0.5, p_m = 0.0$ **(c)** $\gamma = 1.0, p_m = 0.0$ **(d)** $\gamma = 0.0, p_m = 1.5$

(e) $\gamma = 0.0, p_m = 0.5$ **(f)** $\gamma = 0.5, p_m = 0.5$ **(g)** $\gamma = 1.0, p_m = 0.5$ **(h)** $\gamma = 1.5, p_m = 0.5$

Supplementary Figure 29. Opinion evolution in the Algorithmic Bias model with media interactions in the polarised setting, i.e. with two media with opinion 0.05 and 0.95 respectively. Opinion evolution for $\varepsilon = 0.2$.

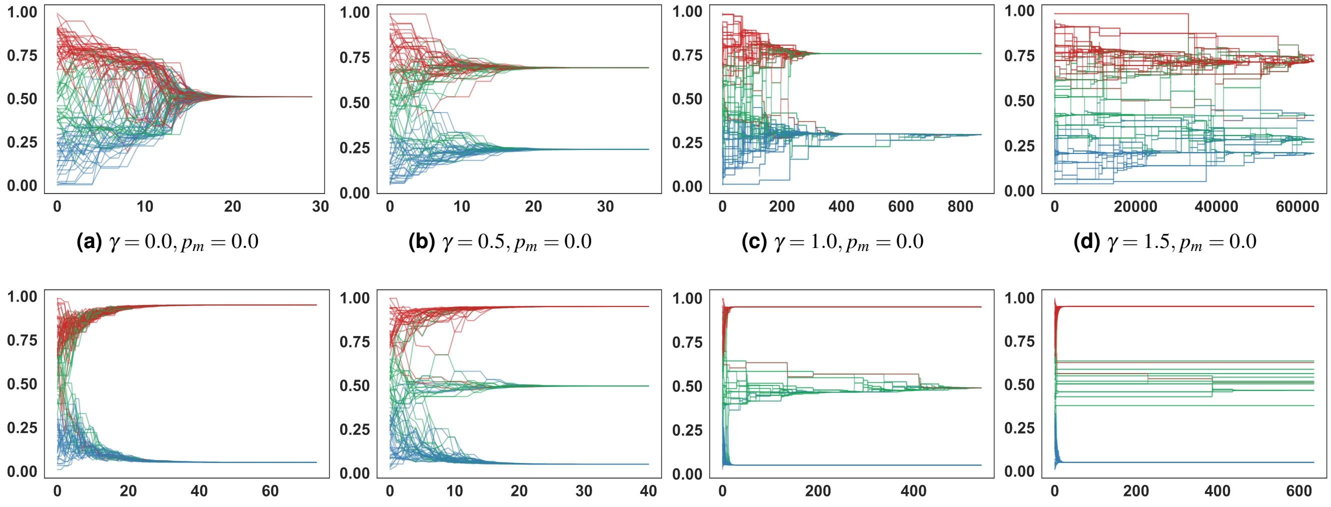

(a) $\gamma = 0.0, p_m = 0.0$ **(b)** $\gamma = 0.5, p_m = 0.0$ **(c)** $\gamma = 1.0, p_m = 0.0$ **(d)** $\gamma = 1.5, p_m = 0.0$

(e) $\gamma = 0.0, p_m = 0.5$ **(f)** $\gamma = 0.5, p_m = 0.5$ **(g)** $\gamma = 1.0, p_m = 0.5$ **(h)** $\gamma = 1.5, p_m = 0.5$

Supplementary Figure 30. Opinion evolution in the Algorithmic Bias model with media interactions in the polarised setting, i.e. with two media with opinion 0.05 and 0.95 respectively. Opinion evolution for $\varepsilon = 0.3$.

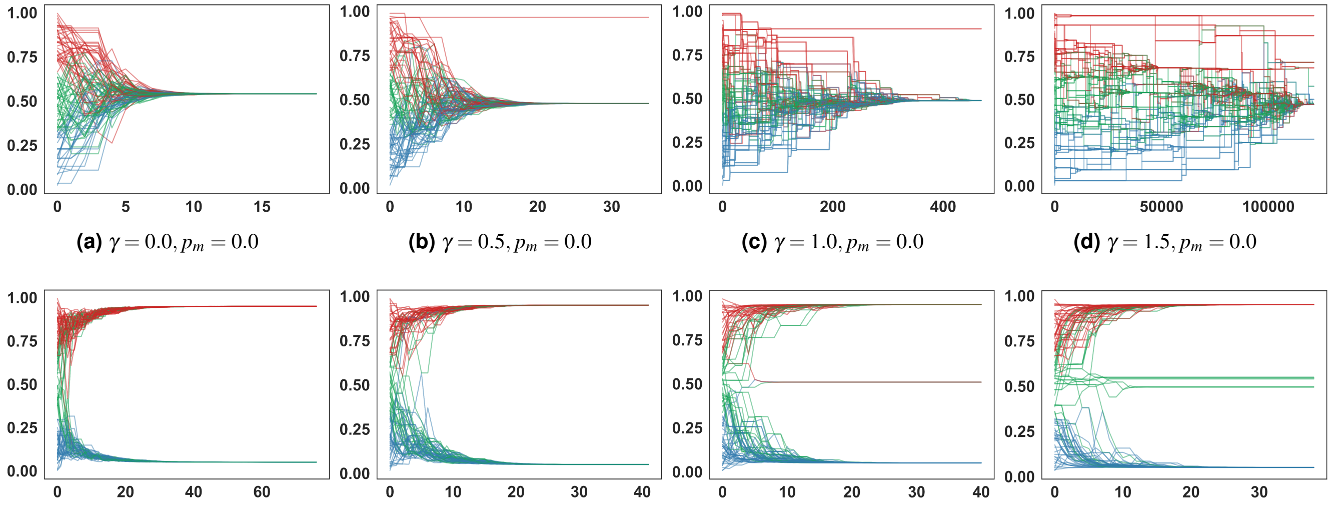

(a) $\gamma = 0.0, p_m = 0.0$ **(b)** $\gamma = 0.5, p_m = 0.0$ **(c)** $\gamma = 1.0, p_m = 0.0$ **(d)** $\gamma = 1.5, p_m = 0.0$

(e) $\gamma = 0.0, p_m = 0.5$ **(f)** $\gamma = 0.5, p_m = 0.5$ **(g)** $\gamma = 1.0, p_m = 0.5$ **(h)** $\gamma = 1.5, p_m = 0.5$

Supplementary Figure 31. Opinion evolution in the Algorithmic Bias model with media interactions in the polarised setting, i.e. with two media with opinion 0.05 and 0.95 respectively. Opinion evolution for $\varepsilon = 0.4$.

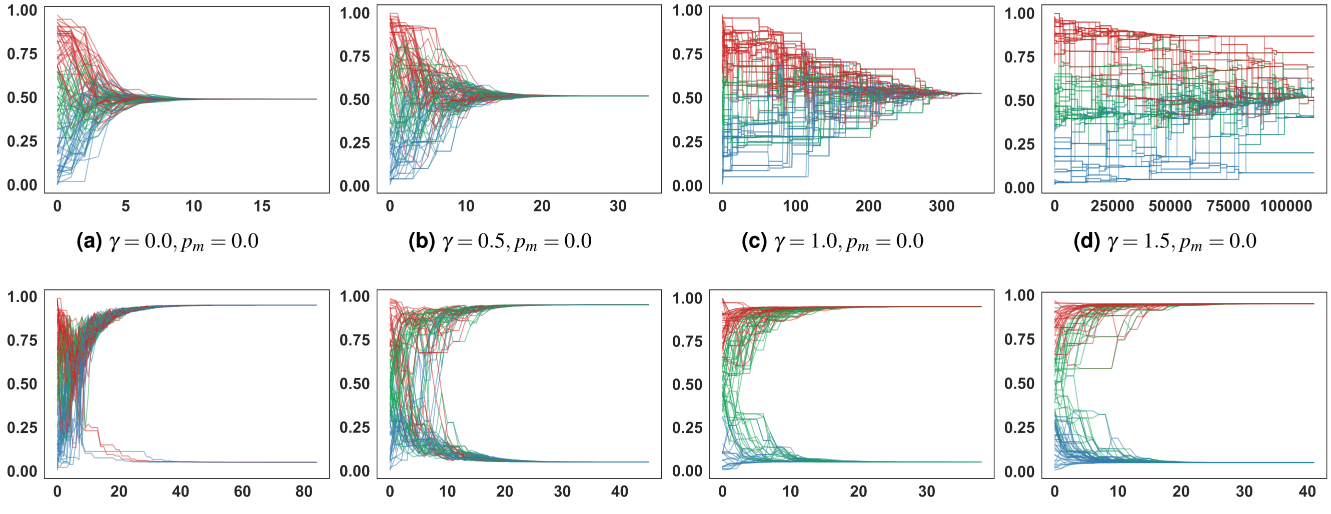

(a) $\gamma = 0.0, p_m = 0.0$ **(b)** $\gamma = 0.5, p_m = 0.0$ **(c)** $\gamma = 1.0, p_m = 0.0$ **(d)** $\gamma = 1.5, p_m = 0.0$

(e) $\gamma = 0.0, p_m = 0.5$ **(f)** $\gamma = 0.5, p_m = 0.5$ **(g)** $\gamma = 1.0, p_m = 0.5$ **(h)** $\gamma = 1.5, p_m = 0.5$

Supplementary Figure 32. Opinion evolution in the Algorithmic Bias model with media interactions in the polarised setting, i.e. with two media with opinion 0.05 and 0.95 respectively. Opinion evolution for $\varepsilon = 0.5$.

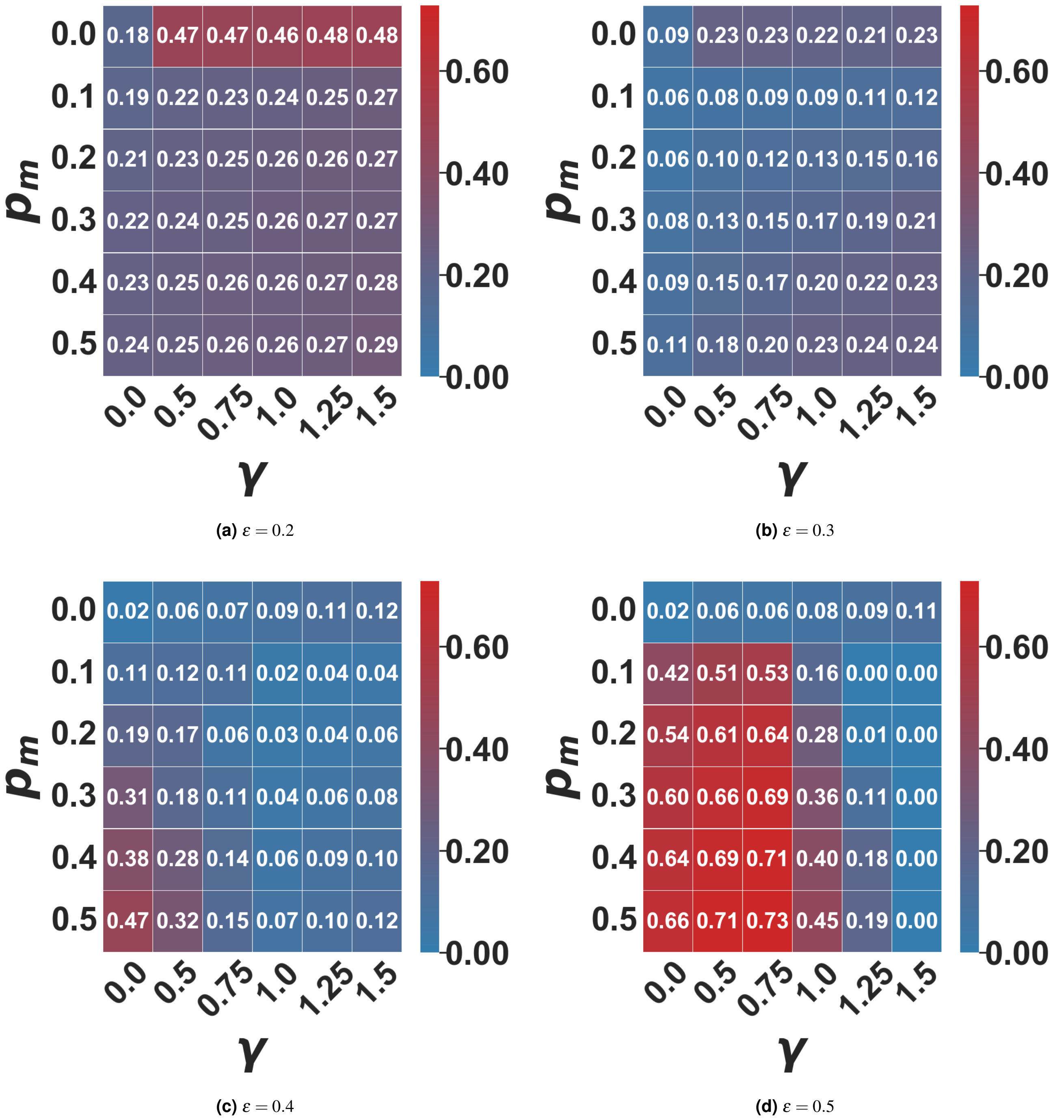

Supplementary Figure 33. Average normalised entropy in the Algorithmic Bias model with media interactions for different values of ε as a function of γ and p_m in the balanced setting. In the figure the average values of the entropy of the final opinion distribution are represented as a function of the algorithmic bias γ and the probability of user-media interaction p_m and are normalised in the range $[0,1]$ where 0 means that every agent holds the same opinion and 1 means that final opinions are uniformly distributed. Values are averaged on 100 independent runs of each setting.

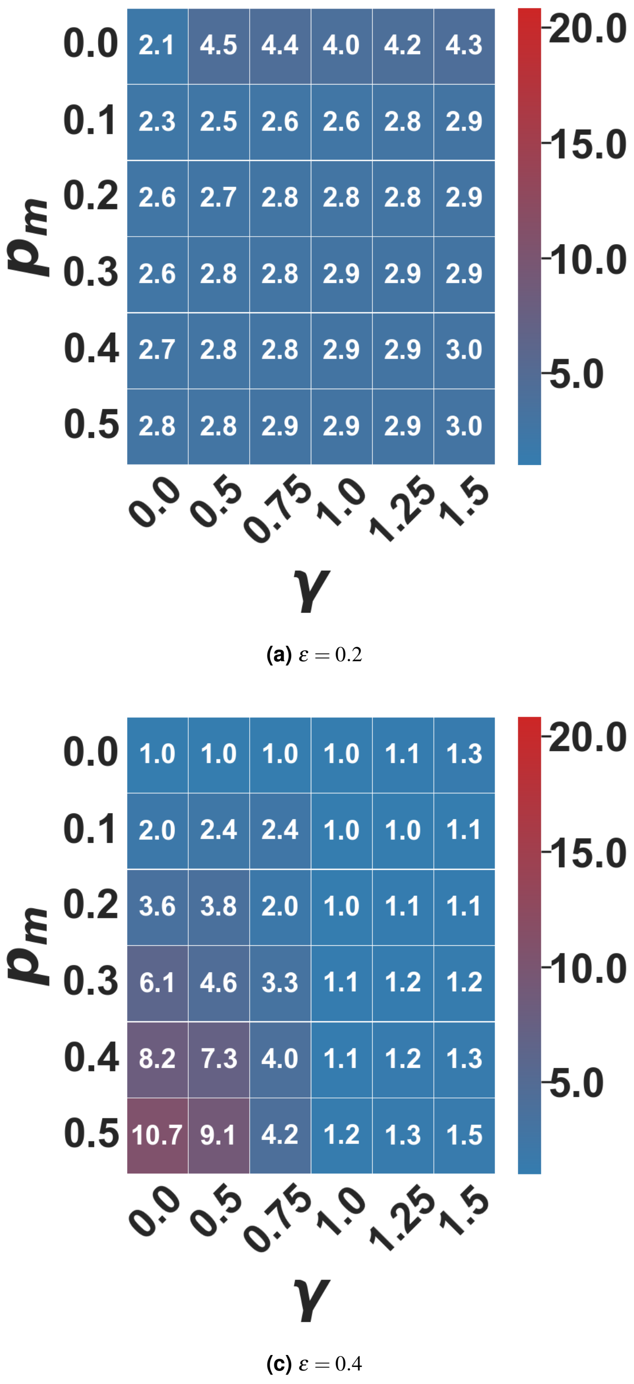

Supplementary Figure 34. Average number of clusters in the Algorithmic Bias model with media interactions for different values of ε as a function of γ and p_m in the balanced setting. In the figure the average number of clusters of the final opinion distribution are represented as a function of the algorithmic bias γ and the probability of user-media interaction p_m. Values are averaged on 100 independent runs of each setting.

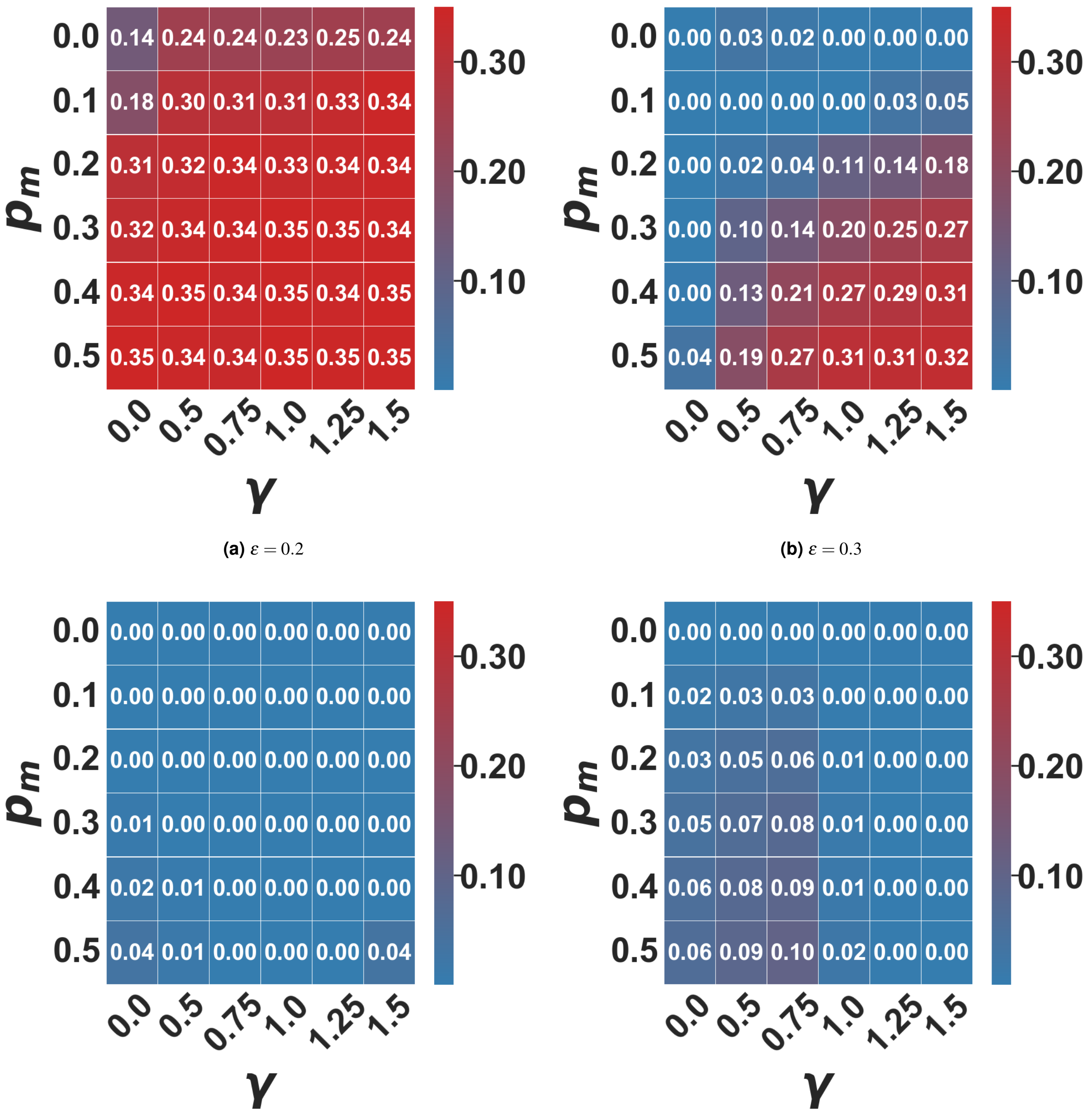

(a) $\varepsilon = 0.2$

(b) $\varepsilon = 0.3$

(c) $\varepsilon = 0.4$

(d) $\varepsilon = 0.5$

Supplementary Figure 35. Average pairwise opinion distance in the Algorithmic Bias model with media interactions for different values of ε as a function of γ and p_m in the balanced setting. In the figure the average pairwise opinion distance of the final distribution is represented as a function of the algorithmic bias γ and the probability of user-media interaction p_m. Values are averaged on 100 independent runs of each setting.

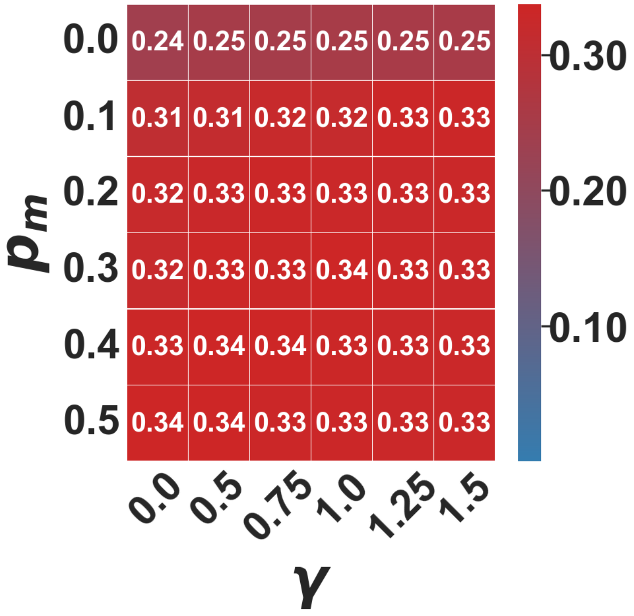

(a) $\varepsilon = 0.2$

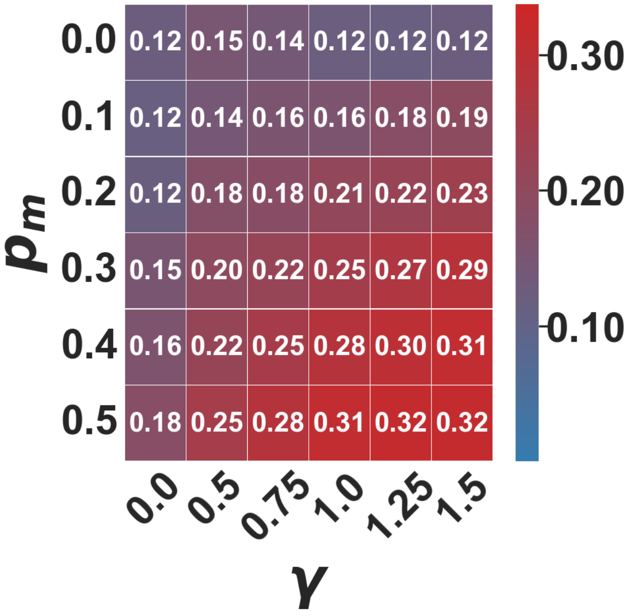

(b) $\varepsilon = 0.3$

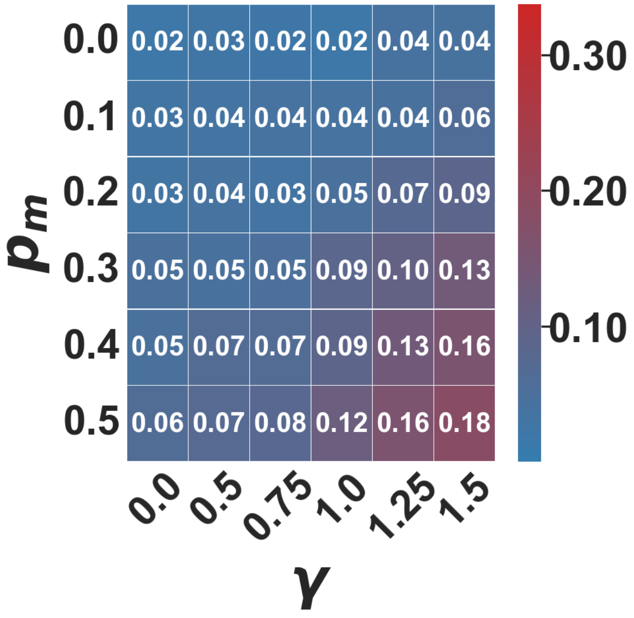

(c) $\varepsilon = 0.4$

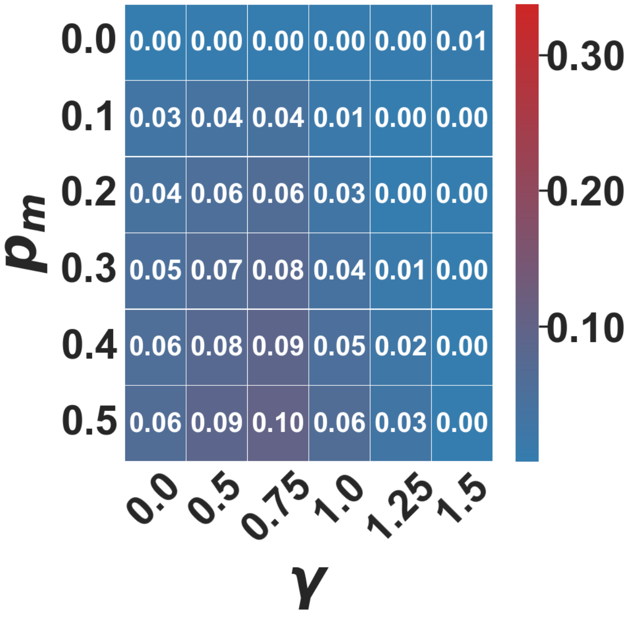

(d) $\varepsilon = 0.5$

Supplementary Figure 36. Average standard deviation of the final opinion distribution in the Algorithmic Bias model with media interactions for different values of ε as a function of γ and p_m in the balanced setting. In the figure the standard deviation of the final opinion distribution is represented as a function of the algorithmic bias γ and the probability of user-media interaction p_m. Values are averaged on 100 independent runs of each setting.

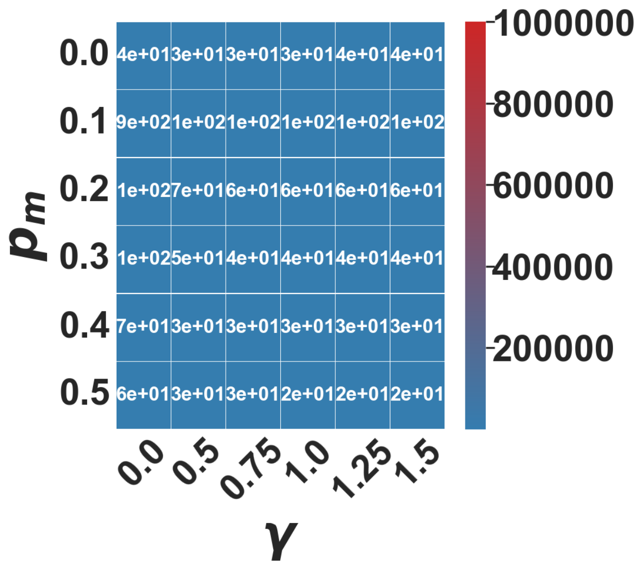

(a) $\varepsilon = 0.2$

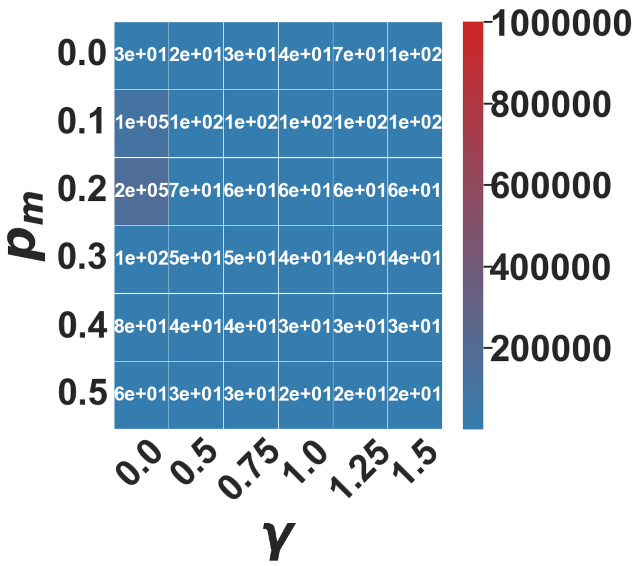

(b) $\varepsilon = 0.3$

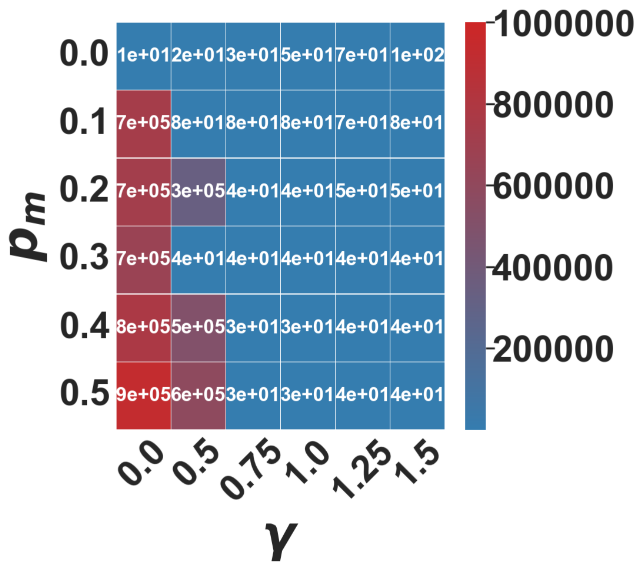

(c) $\varepsilon = 0.4$

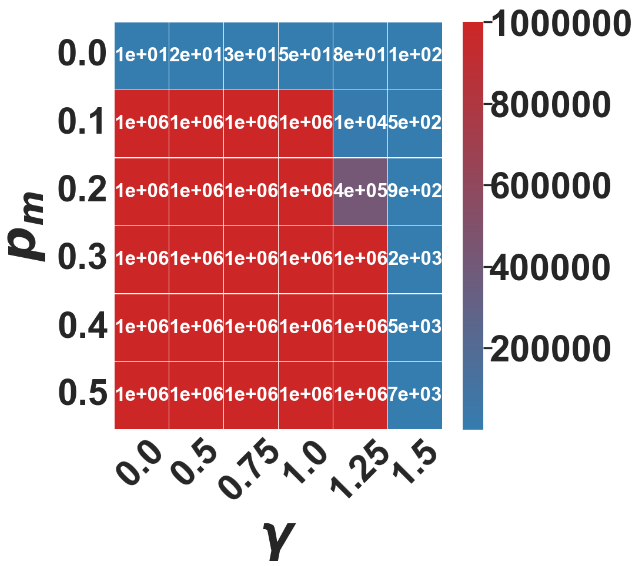

(d) $\varepsilon = 0.5$

Supplementary Figure 37. Average number of iterations at convergence of the final opinion distribution in the Algorithmic Bias model with media interactions for different values of ε as a function of γ and p_m in the balanced setting. In the figure the number of iterations at convergence is represented as a function of the algorithmic bias γ and the probability of user-media interaction p_m. Values are averaged on 100 independent runs of each setting.

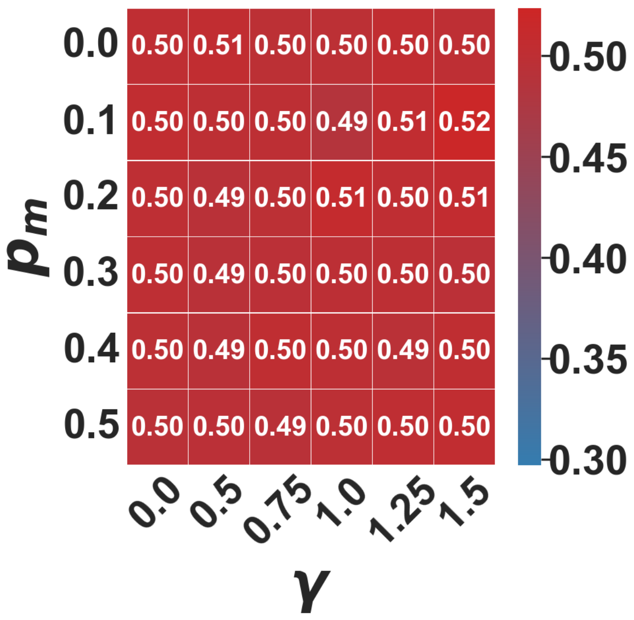

(a) $\varepsilon = 0.2$

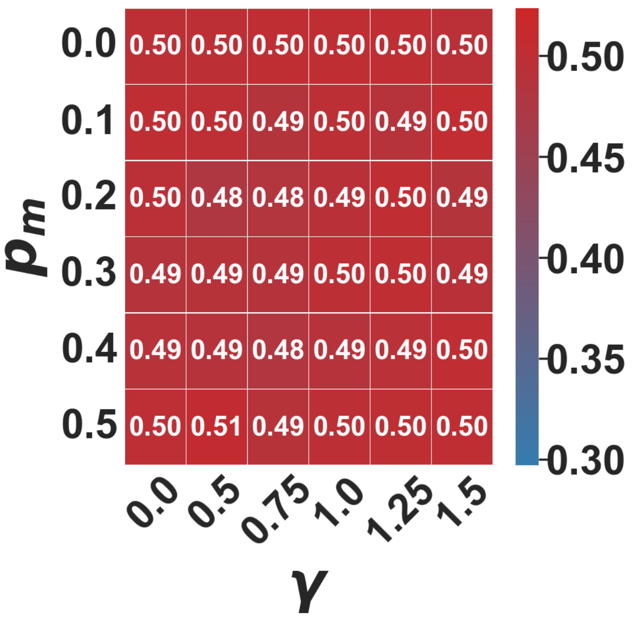

(b) $\varepsilon = 0.3$

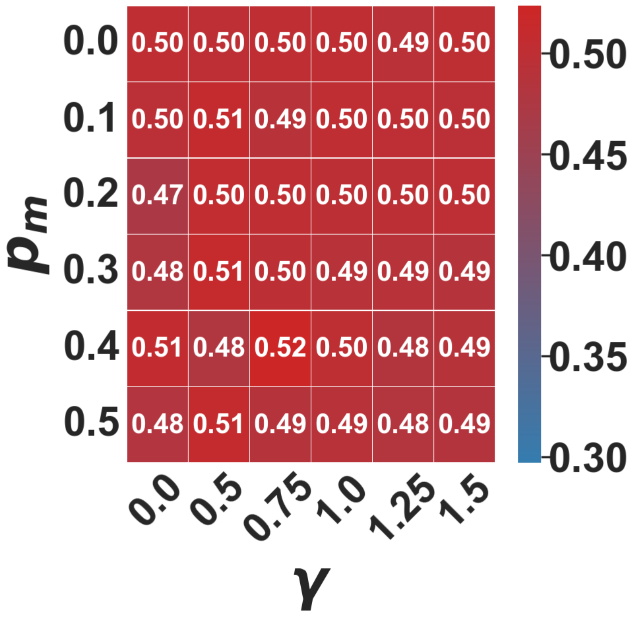

(c) $\varepsilon = 0.4$

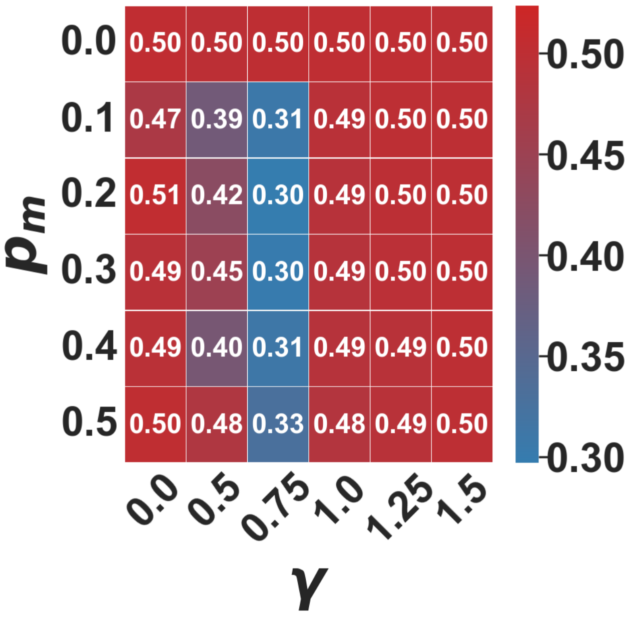

(d) $\varepsilon = 0.5$

Supplementary Figure 38. Average opinion in the Algorithmic Bias model with media interactions for different values of ε as a function of γ and p_m in the balanced setting. In the figure the average opinion of the final opinion distribution are represented as a function of the algorithmic bias γ and the probability of user-media interaction p_m. Values are averaged on 100 independent runs of each setting.

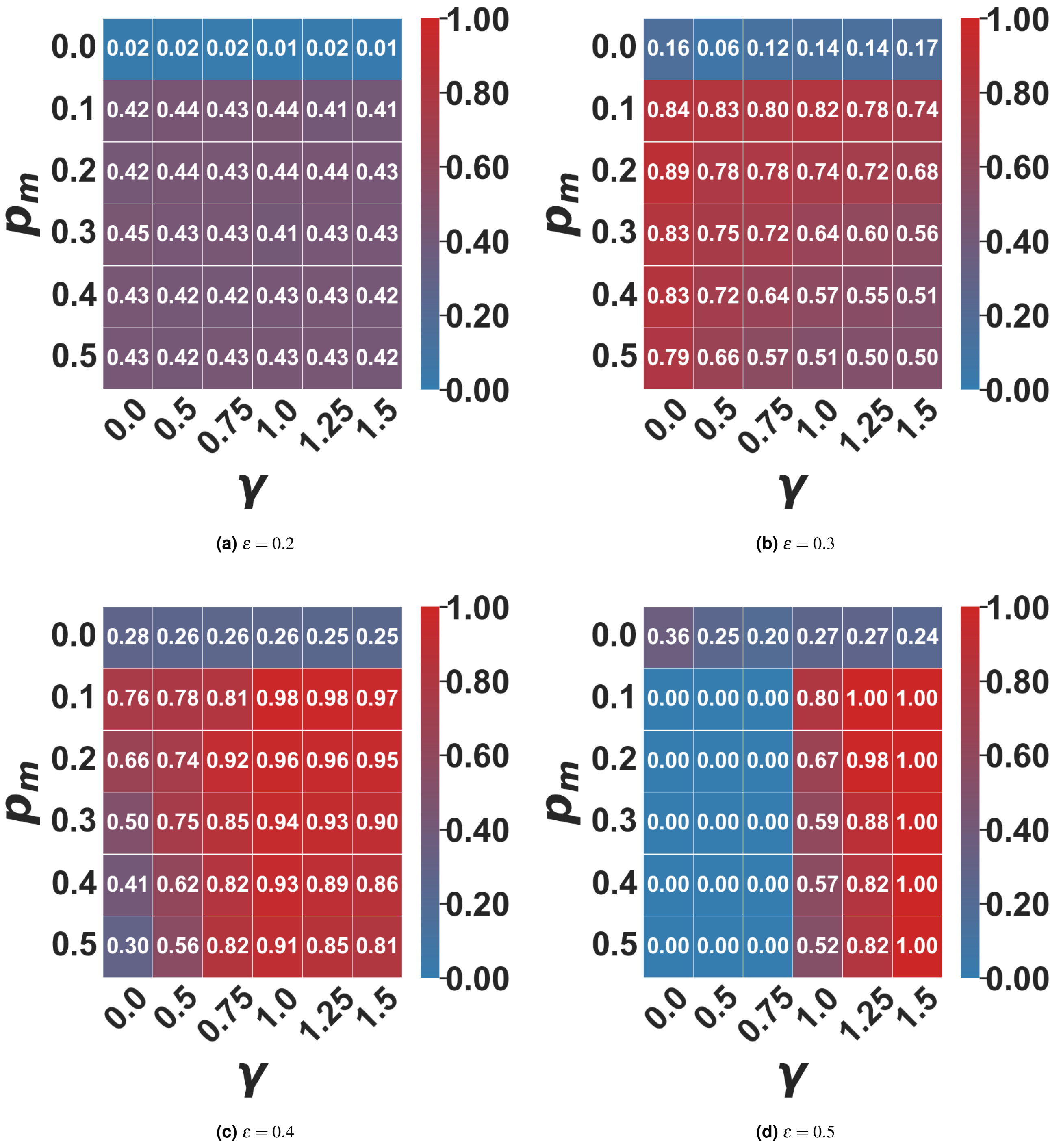

Supplementary Figure 39. Average percentage of agent in the 0.5 cluster in the Algorithmic Bias model with media interactions for different values of ε as a function of γ and p_m in the balanced setting. In the figure the average percentage of agents in the 0.5 cluster are represented as a function of the algorithmic bias γ and the probability of user-media interaction p_m. Values are averaged on 100 independent runs of each setting.

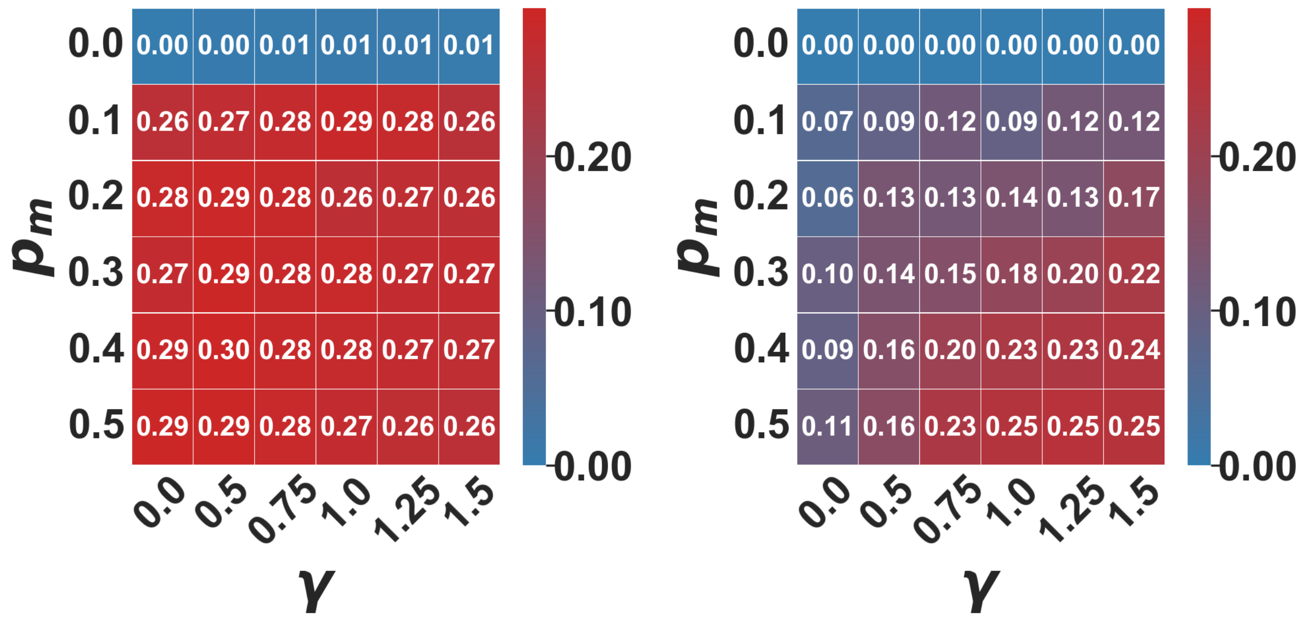

(a) $\varepsilon = 0.2$

(b) $\varepsilon = 0.3$

(c) $\varepsilon = 0.4$

(d) $\varepsilon = 0.5$

Supplementary Figure 40. Average percentage of agent in the 0.05 cluster in the Algorithmic Bias model with media interactions for different values of ε as a function of γ and p_m in the balanced setting. In the figure the average percentage of agents in the 0.05 cluster are represented as a function of the algorithmic bias γ and the probability of user-media interaction p_m. Values are averaged on 100 independent runs of each setting.

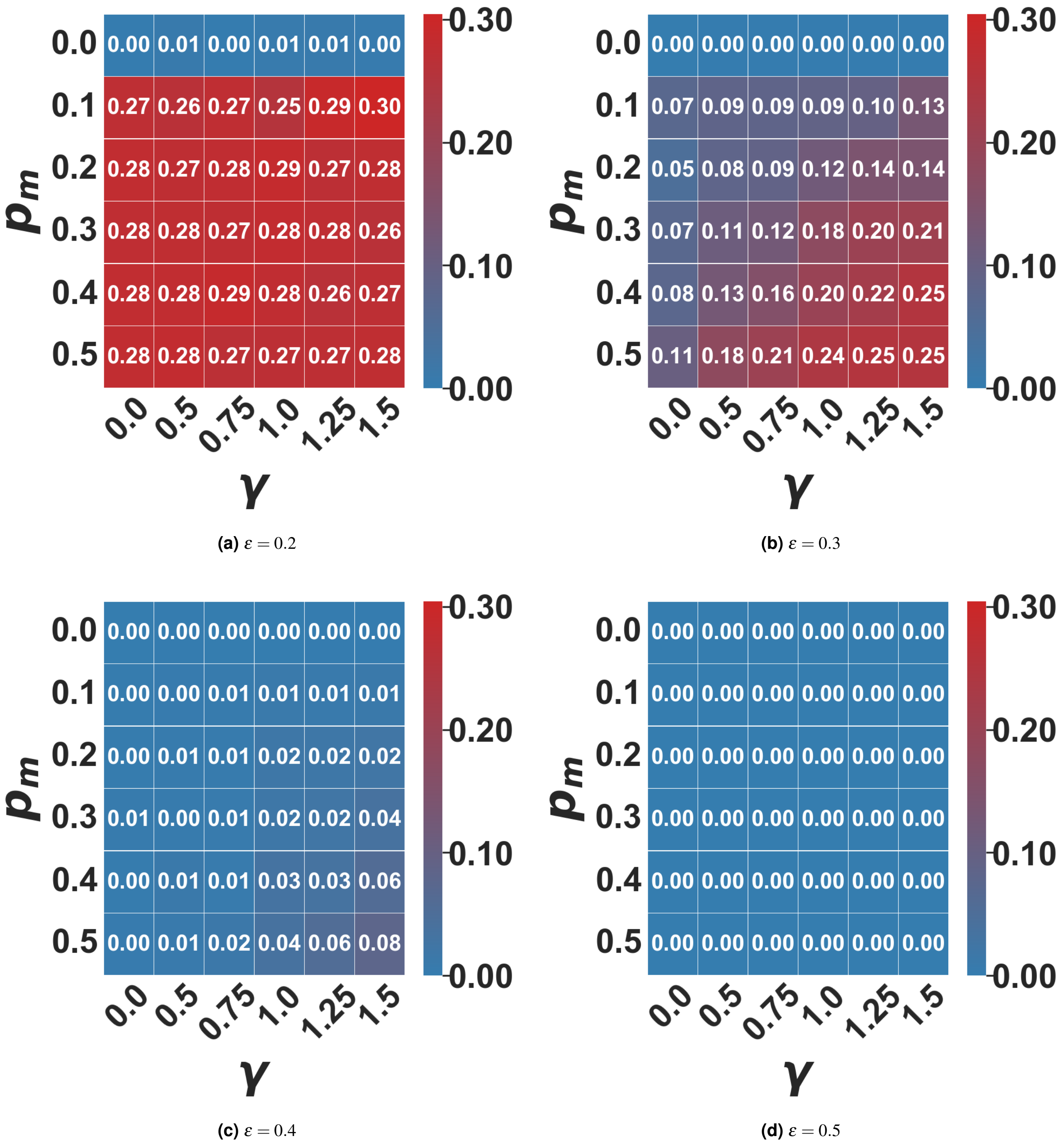

(a) $\varepsilon = 0.2$ (b) $\varepsilon = 0.3$ (c) $\varepsilon = 0.4$ (d) $\varepsilon = 0.5$

Supplementary Figure 41. Average percentage of agent in the 0.95 cluster in the Algorithmic Bias model with media interactions for different values of ε as a function of γ and p_m in the balanced setting. In the figure the average percentage of agents in the 0.95 cluster are represented as a function of the algorithmic bias γ and the probability of user-media interaction p_m. Values are averaged on 100 independent runs of each setting.

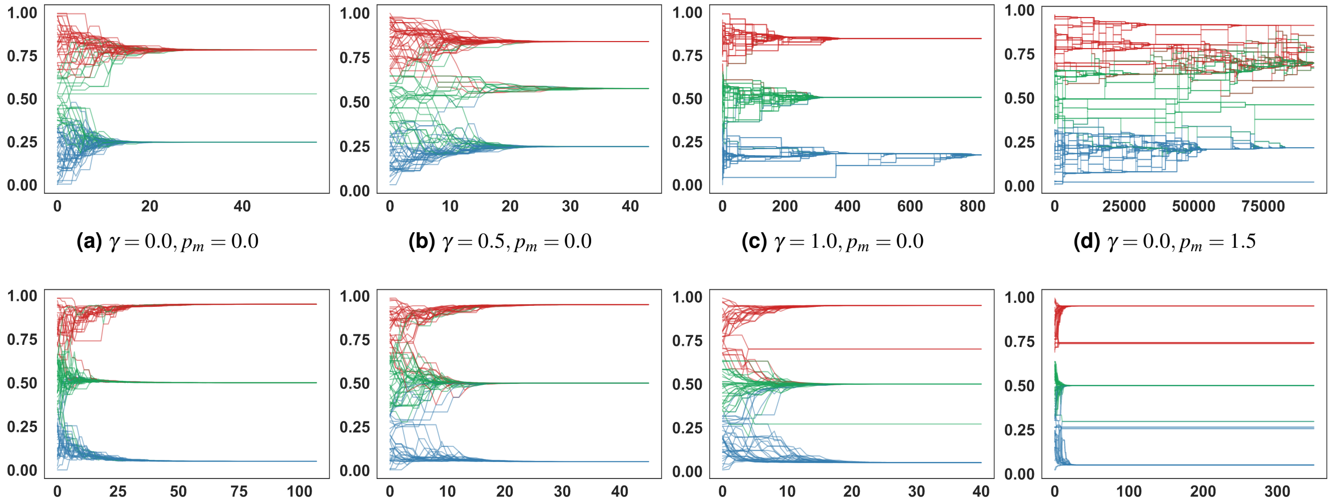

(a) $\gamma = 0.0, p_m = 0.0$ **(b)** $\gamma = 0.5, p_m = 0.0$ **(c)** $\gamma = 1.0, p_m = 0.0$ **(d)** $\gamma = 0.0, p_m = 1.5$

(e) $\gamma = 0.0, p_m = 0.5$ **(f)** $\gamma = 0.5, p_m = 0.5$ **(g)** $\gamma = 1.0, p_m = 0.5$ **(h)** $\gamma = 1.5, p_m = 0.5$

Supplementary Figure 42. Opinion evolution in the Algorithmic Bias model with media interactions in the balanced setting. Opinion evolution for $\varepsilon = 0.2$.

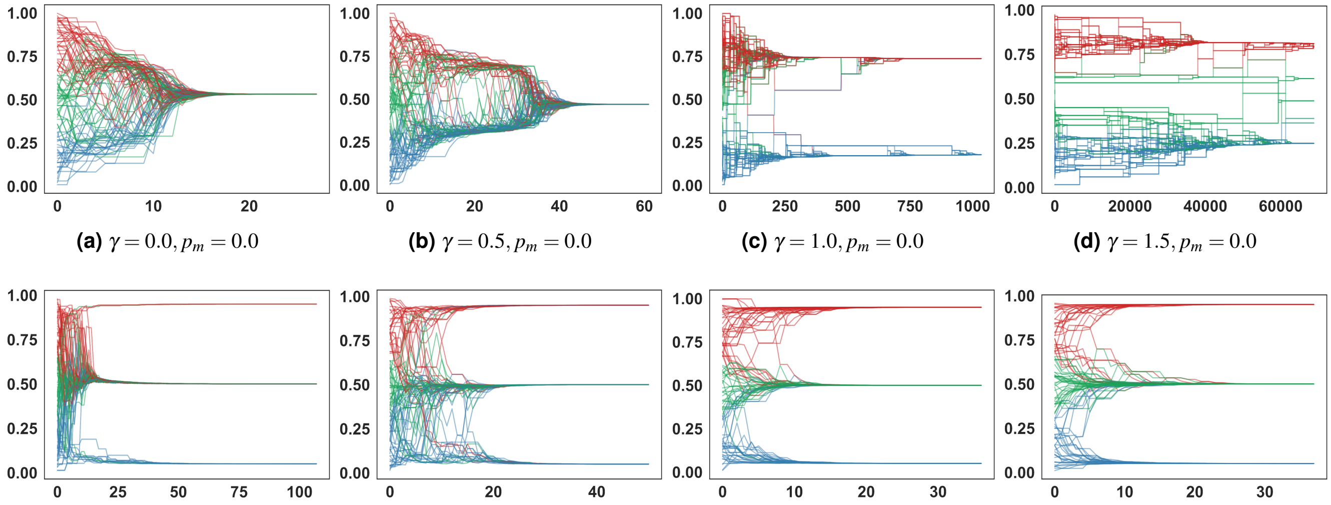

(a) $\gamma = 0.0, p_m = 0.0$ **(b)** $\gamma = 0.5, p_m = 0.0$ **(c)** $\gamma = 1.0, p_m = 0.0$ **(d)** $\gamma = 1.5, p_m = 0.0$

(e) $\gamma = 0.0, p_m = 0.5$ **(f)** $\gamma = 0.5, p_m = 0.5$ **(g)** $\gamma = 1.0, p_m = 0.5$ **(h)** $\gamma = 1.5, p_m = 0.5$

Supplementary Figure 43. Opinion evolution in the Algorithmic Bias model with media interactions in the balanced setting. Opinion evolution for $\varepsilon = 0.3$.

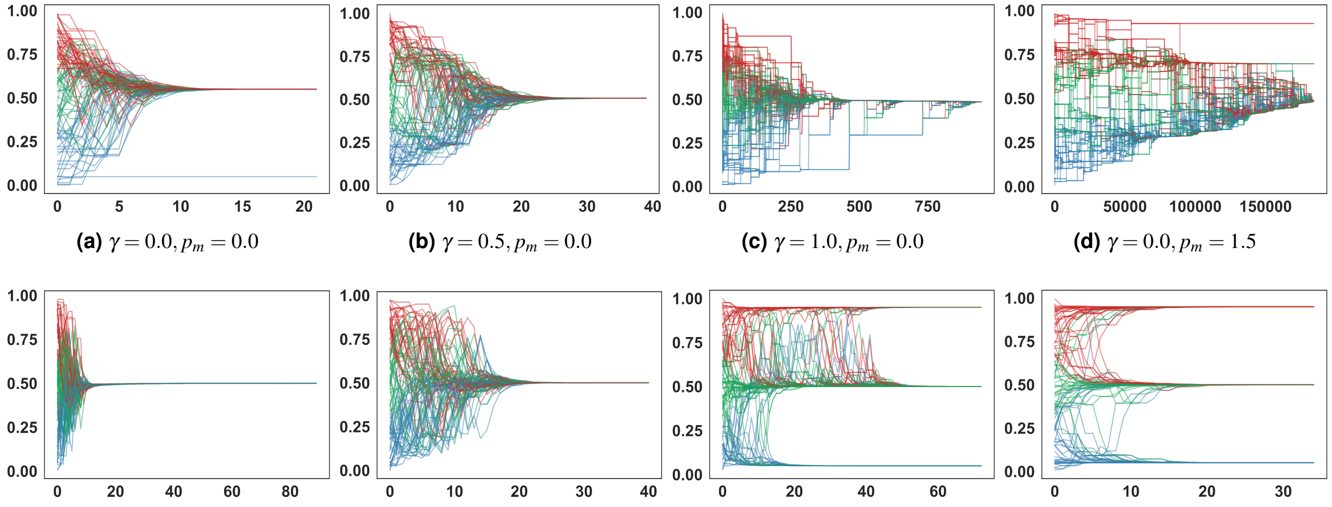

(a) $\gamma = 0.0, p_m = 0.0$ **(b)** $\gamma = 0.5, p_m = 0.0$ **(c)** $\gamma = 1.0, p_m = 0.0$ **(d)** $\gamma = 0.0, p_m = 1.5$

(e) $\gamma = 0.0, p_m = 0.5$ **(f)** $\gamma = 0.5, p_m = 0.5$ **(g)** $\gamma = 1.0, p_m = 0.5$ **(h)** $\gamma = 1.5, p_m = 0.5$

Supplementary Figure 44. Opinion evolution in the Algorithmic Bias model with media interactions in the balanced setting. Opinion evolution for $\varepsilon = 0.4$.

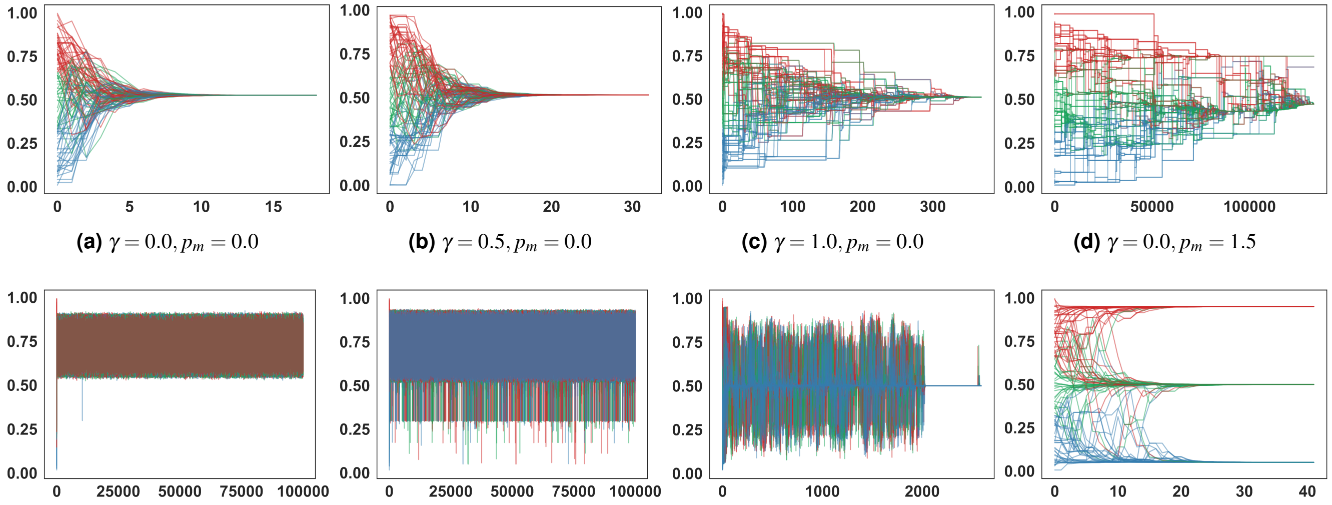

(a) $\gamma = 0.0, p_m = 0.0$ **(b)** $\gamma = 0.5, p_m = 0.0$ **(c)** $\gamma = 1.0, p_m = 0.0$ **(d)** $\gamma = 0.0, p_m = 1.5$

(e) $\gamma = 0.0, p_m = 0.5$ **(f)** $\gamma = 0.5, p_m = 0.5$ **(g)** $\gamma = 1.0, p_m = 0.5$ **(h)** $\gamma = 1.5, p_m = 0.5$

Supplementary Figure 45. Opinion evolution in the Algorithmic Bias model with media interactions in the balanced setting. Opinion evolution for $\varepsilon = 0.5$.

Case Study on Euro2020 echo chamber evolution on Twitter

In the following section we report details on data collection, labelling and network construction for the dataset employed in the case study that were not reported in the Materials and Methods section of the manuscript due to space limitations.

Data collection

The dataset covers a period of around one month, starting from June 10th and concluding on July 13th. We filtered the conversations included in the dataset using specific hashtags related to Italy's matches, the competition, and the topic of taking the knee. In total, we collected 38908 tweets from 16235 unique users. To analyze the opinions of Twitter users regarding taking the knee during EURO 2020, we employed a hashtag-based approach. We manually annotated 2,304 hashtags from the dataset and assigned a numerical value to each hashtag. Hashtags expressing a clear position for or against taking the knee were assigned a value of ± 3, hashtags closely associated with either faction were given a value of ± 1, while neutral or irrelevant hashtags were assigned a value of 0. For each tweet, we calculated its classification value (C_t) by averaging the non-neutral hashtag classification values (C_h) within it. Similarly, for each user (u), we determined their overall classification value (C_u) by averaging the classification values of their tweets. To utilize this dataset as a case study for our opinion dynamics model, we transformed the initial pro/against score, ranging from -3 to 3, into a normalized range of $[0,1]$. We also discretized these leanings into three intervals: "Pro" if $C_u \leq 0.4$, "Against" if $C_u \geq 0.6$, and "Neutral" otherwise. By incorporating a third label, we ensure the inclusion of users with highly polarized viewpoints.

Network Construction

We built an undirected graph, where nodes are the users, and the edges represent their interactions (i.e. retweet, mention, quote and reply). The network is composed by $N = 15378$ nodes and $L = 36496$ edges. We divided the network into two snapshots, namely the first one corresponding to the group stage and round of 16, and the second one corresponding to the period from quarterfinals to the final, in order to obtain initial and final situations for validating our model. For the purpose of this study, we made the decision not to consider the temporal evolution of links, as our model does not account for the creation and dissolution of links during the process of opinion exchange. Consequently, we retained only the nodes that are present in both snapshots. However, the links were flattened, as we actually have a single graph G, and the only temporal element considered is the changing opinion, thus creating two different undirected snapshot networks G_0, with nodes labelled according to their leaning in the first period and G_1, with nodes labelled according to their leaning in the second period. This approach ensures a sufficiently similar baseline to our model, which considers a static network. The two snapshot graphs consist of $N = 2925$ users (approximately 20% of the total) and $L = 9081$ edges. The number of nodes in the giant connected component is 2894 and the number of edges is 9054. The analysis reveals that the degree distribution of the graph exhibits properties consistent with a power law distribution. The power law fit confirms that the degree distribution can be effectively approximated by a power law model. The calculated power law exponent (α) of 2.47 indicates a slower decay of the distribution, suggesting the presence of a heavy-tailed degree distribution. The minimum degree required for the power law fit is identified as 5.0, indicating that the power law behavior is observed for degrees equal to or greater than this threshold. The p-value from both the log-likelihood ratio test and the Kolmogorov-Smirnov test is 0.03, indicating a relatively low probability of obtaining test results as extreme as the observed data under the assumption of a power law distribution. This suggests reasonable agreement between the observed degree distribution and the power law model (see Supplementary Figure 46). The analysis of the opinion distribution across the population, presented in Supplementary Figure 47, reveals noteworthy dynamics between the first (G_0) and second (G_1) period. Initially, there is a relatively balanced distribution between "pro" and "against" users, but in the second period, the number of "against" users decreases while the number of "neutral" and "pro" users increases as we can see from Supplementary Table 1. We also computed the average opinion for each leaning in G_0:

- $avg(pro) = 0.28$
- $avg(neutral) = 0.49$
- $avg(against) = 0.87$

which we then used to assign an opinion to the mass media in the simulations of the present model on this empirical network.

Supplementary Table 1. Number of users for each discretized leaning

	Pro	Neutral	Against
G_0	1004	413	1508
G_1	1248	451	1226

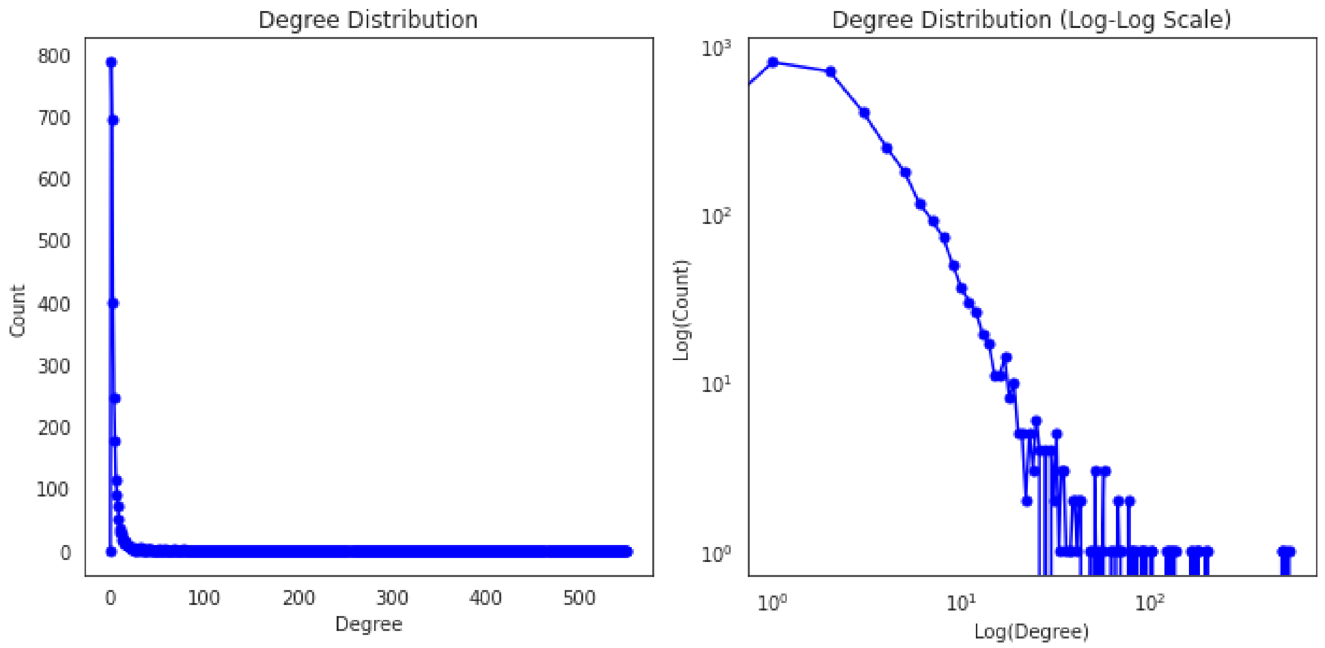

Supplementary Figure 46. Degree distribution (left) and log-log degree distribution (right).

To gain further insights, conformity distributions are examined. Conformity, defined in[1], in this context refers to a local measure quantifying the prevalence of homophilic connections among network nodes based on shared attributes. A conformity value of 1.0 indicates that a node is predominantly surrounded by neighbors who hold the same attribute value, while -1.0 suggests the opposite scenario. The analysis of conformity distributions during the first period, represented by the red histograms in Supplementary Figure 48, reveals a negatively skewed pattern among users against taking the knee. This indicates a prevalence of homophilic connections within this group, where individuals tend to be connected to others with similar viewpoints. Furthermore, as the parameter α increases, which governs the level of interaction between nodes with diminishing influence as distance increases, homophily becomes more pronounced among users against taking the knee. In other words, the impact of nearby nodes outweighs that of distant nodes in determining conformity patterns within this group. Neutral users ($0.4 < C_u < 0.6$), in contrast, exhibit a disassortative behavior, primarily connecting with users who hold well-defined positions on the matter rather than other neutral users. Users who support taking the knee display a varied range of conformity levels, with a tendency towards homophily as α increases. However, it is observed that this cluster of users interacts with both like-minded and opposing profiles without a clear preferential behavior. A similar analysis conducted during the second period, depicted in Supplementary Figure 49, reveals that neutral users still exhibit a disassortative behavior regarding their stance on taking the knee. On the other hand, users against taking the knee demonstrate a connectivity pattern that is randomly mixed, although it becomes more homophilic with higher values of α. However, the distribution is less skewed compared to the first snapshot. Users who support taking the knee also display an assortative behavior for lower values of α, but their conformity distribution becomes negatively skewed, similar to the previous period. In both cases, these distributions exhibit long tails when $\alpha \geq 2.0$, suggesting that, on average, user behavior tends to be homophilic. However, there is a portion of both subpopulations with a randomly mixed connectivity pattern, as well as another portion displaying a disassortative behavior. While the opinion distribution appears more balanced in the first period compared to the second period, an assessment of the population's level of polarization, as depicted in Supplementary Figure 50, reveals that the network is less polarized in the second period. This finding contrasts with the analysis in[2], where only the subset of users actively participating in the conversation over the entire duration of the study demonstrates reduced polarization over time, in contrast to the overall population.

Algorithmic Bias Model with Mass Media on Euro2020 Network

Supplementary Figures 51-56 represent the final opinion distribution of the Algorithmic Bias Model with Mass Media for homogeneous ε. As we can see from Supplementary Figures 51-54, when there are no media in the population or when there is a single media, we have consensus in the final state. An exception is the case where we have no media and $\gamma = 1.5$ where the final opinion distribution is fragmented with two main clusters around the average leaning of the "pro" faction and the average leaning of the "against" faction. When there are two polarized media (see Supplementary Figure 55) the final

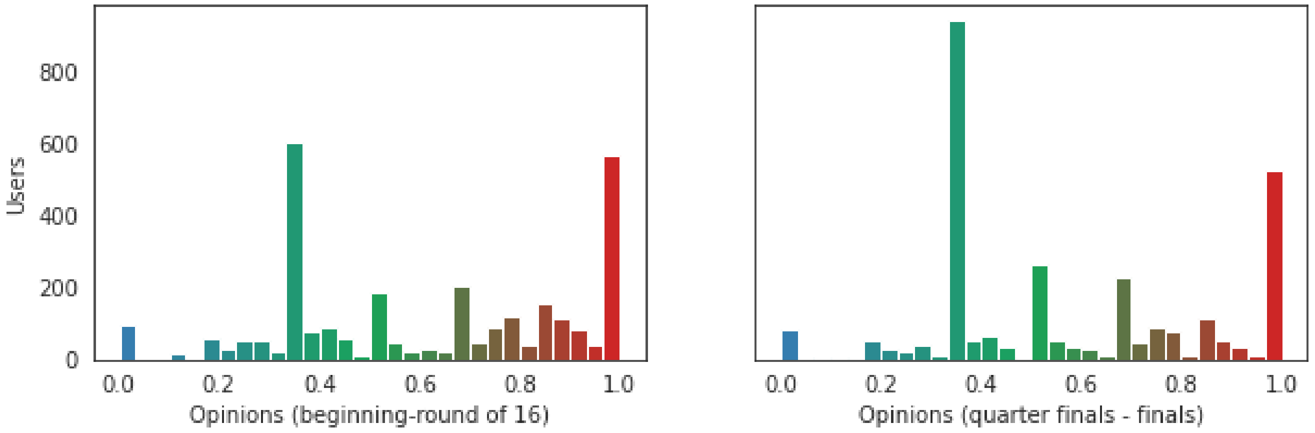

Supplementary Figure 47. Opinion distribution in the first period (left) and in the second period (right).

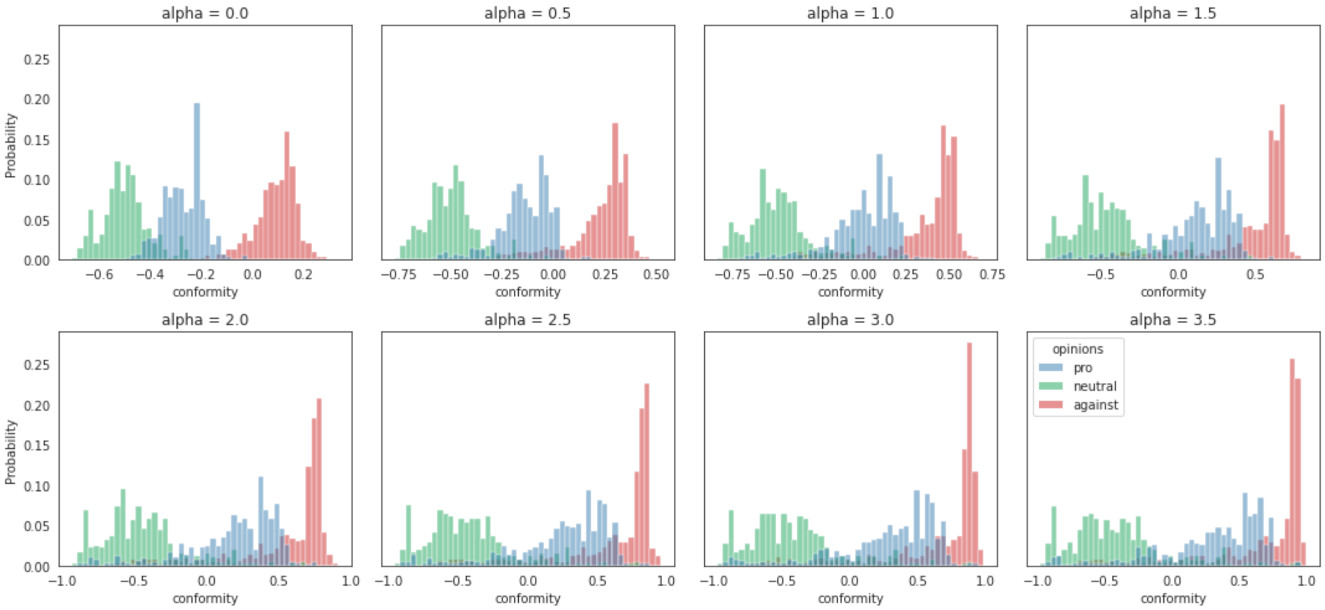

Supplementary Figure 48. Conformity distribution of users who are pro, neutral and against taking the knee. Conformity is computed for $\alpha \in [0.0, 4.0)$ with step = 0.5.

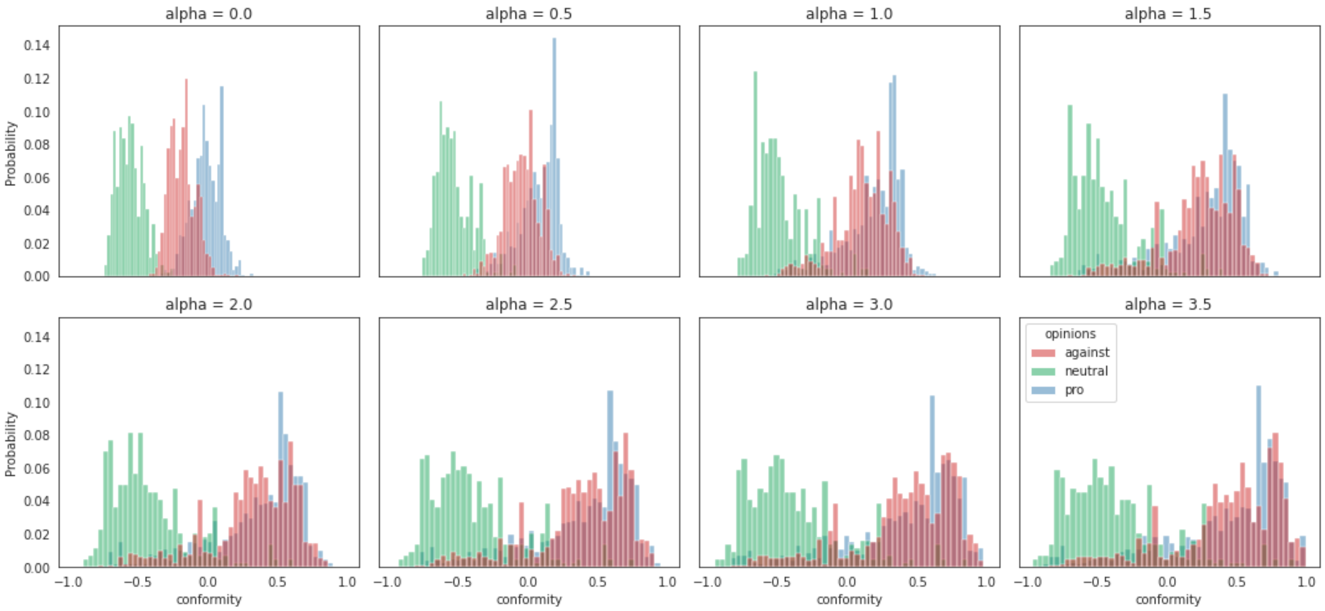

Supplementary Figure 49. Conformity distribution of users who are pro, neutral and against taking the knee. Conformity is computed for $\alpha \in [0.0, 4.0)$ with step = 0.5.

distribution resembles a Gaussian distribution with slightly higher average than the average opinion 0.5. However, we can see that polarization starts manifesting as the bias grows and that there are two well defined opinion clusters when $\gamma = 1.5$ for every value of ε. When there is a balanced media landscape the final opinion distribution is still symmetric and peaked around the center of the opinion spectrum, however it has a lower variance with respect to the previous setting. Moreover variance reduces as the bias grows and for $\gamma = 1.0$ there is one main opinion cluster in the final setting. However, if the bias grows more, i.e. $\gamma = 1.5$, the final distribution splits into well defined opinion clusters around the media opinion.

Bounded Confidence Estimation Procedure and Results

The methodology to estimate the user-level open mindedness employed in the present work was developed in[3] and is described in Alg. 1.

To quantify the influence of users' neighbors on their opinions, we devised a straightforward approach detailed in Algorithm 1. This method leverages the weighted interaction network at time t and the opinions of agents at both time t and $t+1$ as inputs.

Our estimation procedure focuses on nodes for which we possess opinions $x_u(t)$ and $x_u(t+1)$ (line 1 of Algorithm 1). This allows us to gauge the extent to which interactions during this time step influenced opinion changes and, consequently, estimate the level of bounded confidence.

Once a node u is selected, we arrange the interacting partners (i.e., neighbors in the snapshot network) in ascending order based on the absolute value of their opinion distance $d_{u,v}(t) = |x_u(t) - x_i(t)|$ (line 6 of Algorithm 1).

Subsequently, we iteratively compute an estimate $\widehat{x_u}(t+1)$ by averaging the newly obtained estimate with the opinions of the interacting neighbors (line 13 of Algorithm 1).

The final estimated value $\widehat{x_u}(t+1)$ is determined by minimizing the error with respect to the actual value $x_u(t+1)$ (lines 15-22 of Algorithm 1).

To establish a confidence bound, we measure the absolute distance to the neighbor that corresponds to the point of minimum in the sequence of estimation errors (line 25 of Algorithm 1).

This proposed approach enables the estimation of opinions only for the subset of users who are present in consecutive observations (e.g., months) and share at least one interaction in the snapshot graph.

Exploiting the procedure in Alg. 1 applied to G_0 and G_1 we are able to compute estimates of ε_u for each users u in G_0. Distributions of the estimated values for each subpopulation in the present network are depicted in Supplementary Figure 57.

Algorithmic Bias Models with Mass Media and Heterogeneous Confidence Bound on Euro2020 Network

In this section, we include supplementary figures that showcase simulation results not included in the main manuscript due to space limitations. Supplementary Figure 58 illustrates that the algorithmic bias reduces the level of polarization in the interaction network with these specific initial conditions (G_0) and without media influence ($p_m = 0.0$). Specifically, in Supplementary

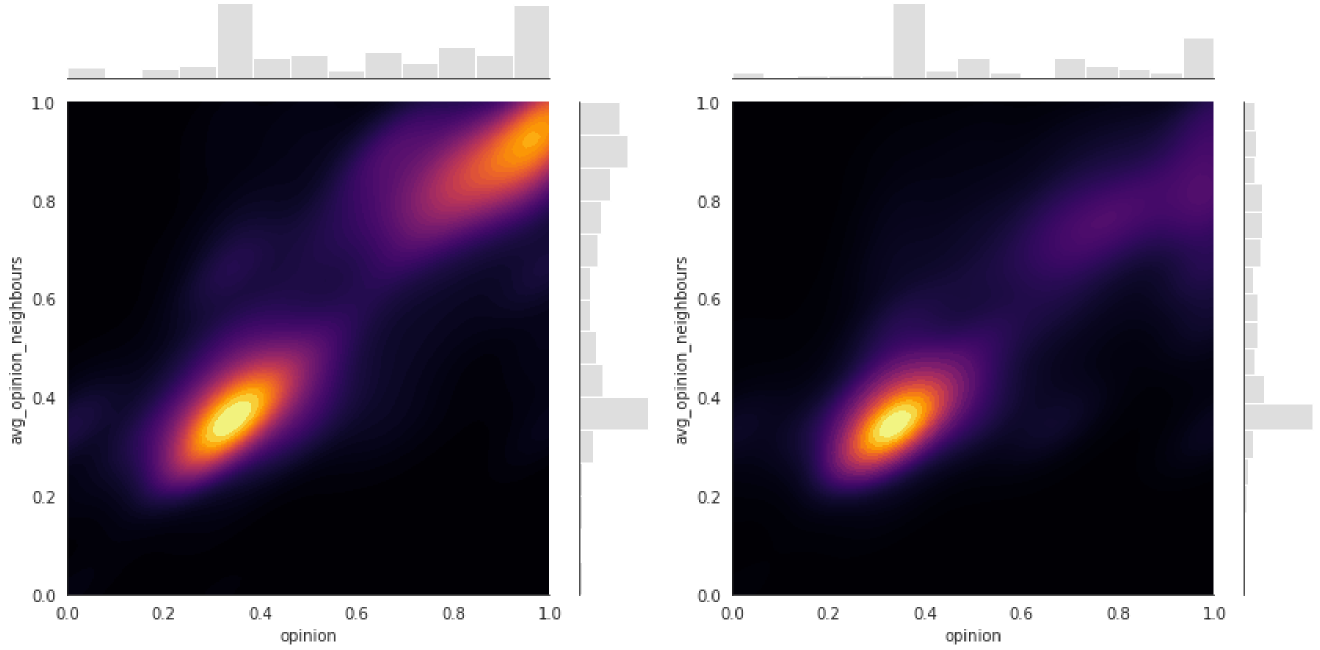

(a) First period G_0 **(b)** Second period G_1

Supplementary Figure 50. Joint distribution of users' opinions and the average leaning of their neighborhood. Joint distribution of users' opinions and the average leaning of their neighborhood in G_0 (a) and G_1 (b). The color intensity in the figures corresponds to the density of users, with lighter colors representing a higher number of users. The marginal distributions are plotted on the x and y axes to provide additional insights. Upon comparing the two snapshots, it is evident that the first period demonstrates a higher degree of network polarization in comparison to the second period. However, both networks exhibit homophilic patterns, indicating a tendency for users to connect with others who share similar viewpoints. More specifically, in G_0, the network displays the presence of two echo chambers. One echo chamber consists of users who hold a leaning in favor of taking the knee (”pro” users), while the other echo chamber comprises users who hold a leaning against taking the knee (”against” users). The ”against” echo chamber is positioned on more extremist stances compared to the ”pro” echo chamber, which is centered around more moderate positions. In G_1, the ”against” echo chamber becomes less pronounced, while the ”pro” echo chamber strengthens, indicating a shift in the network dynamics over time.

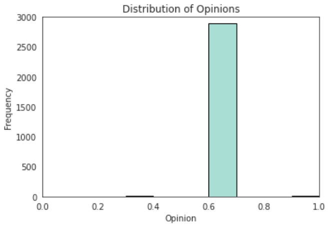

(a) $\varepsilon = 0.2, \gamma = 0.0, p_m = 0.0$

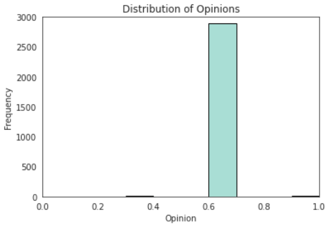

(b) $\varepsilon = 0.2, \gamma = 0.5, p_m = 0.0$

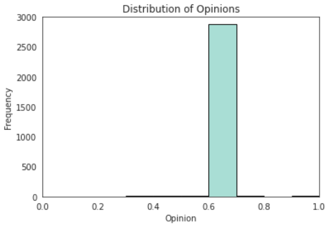

(c) $\varepsilon = 0.2, \gamma = 1.0, p_m = 0.0$

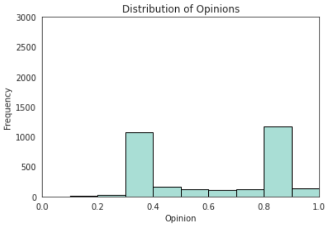

(d) $\varepsilon = 0.2, \gamma = 1.5, p_m = 0.0$

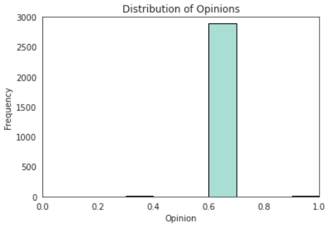

(e) $\varepsilon = 0.3, \gamma = 0.0, p_m = 0.0$

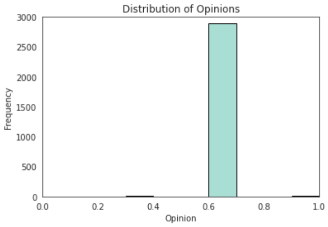

(f) $\varepsilon = 0.3, \gamma = 0.5, p_m = 0.0$

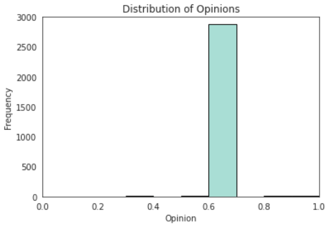

(g) $\varepsilon = 0.3, \gamma = 1.0, p_m = 0.0$

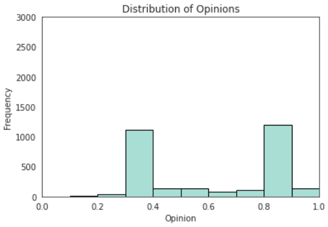

(h) $\varepsilon = 0.3, \gamma = 1.5, p_m = 0.0$

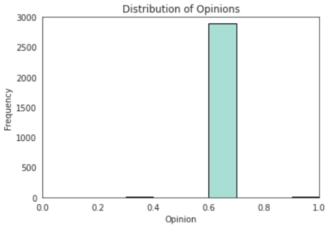

(i) $\varepsilon = 0.4, \gamma = 0.0, p_m = 0.0$

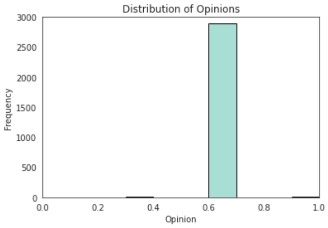

(j) $\varepsilon = 0.4, \gamma = 0.5, p_m = 0.0$

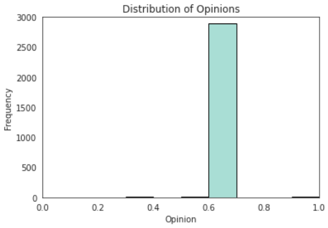

(k) $\varepsilon = 0.4, \gamma = 1.0, p_m = 0.0$

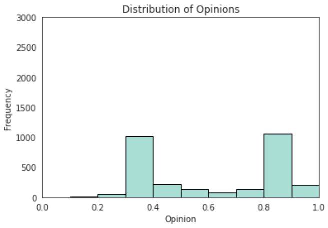

(l) $\varepsilon = 0.4, \gamma = 1.5, p_m = 0.0$

Supplementary Figure 51. Final opinion distribution in the Algorithmic Bias Model ($p_m = 0.0$) for different values of ε and γ. From top to bottom: $\varepsilon = 0.2$, $\varepsilon = 0.3$ and $\varepsilon = 0.4$. From left to right: $\gamma = 0.0$, $\gamma = 0.5$, $\gamma = 1.0$, $\gamma = 1.5$.

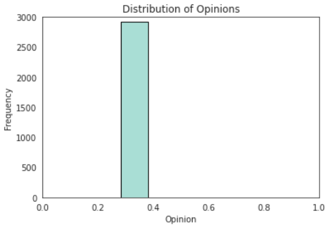

(a) $\varepsilon = 0.2, \gamma = 0.0, p_m = 0.5$

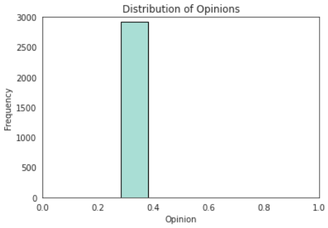

(b) $\varepsilon = 0.2, \gamma = 0.5, p_m = 0.5$

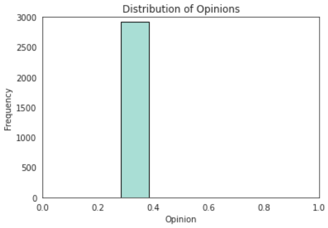

(c) $\varepsilon = 0.2, \gamma = 1.0, p_m = 0.5$

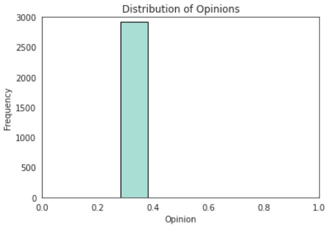

(d) $\varepsilon = 0.2, \gamma = 1.5, p_m = 0.5$

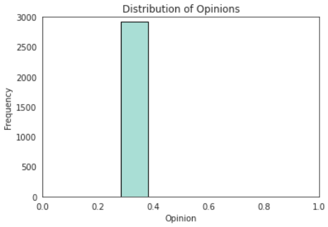

(e) $\varepsilon = 0.3, \gamma = 0.0, p_m = 0.5$

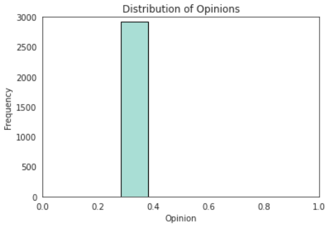

(f) $\varepsilon = 0.3, \gamma = 0.5, p_m = 0.5$

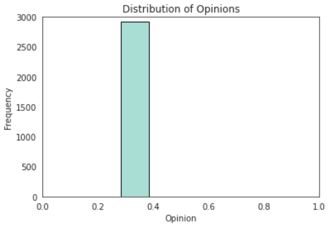

(g) $\varepsilon = 0.3, \gamma = 1.0, p_m = 0.5$

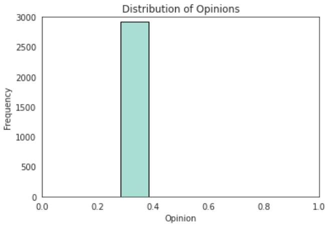

(h) $\varepsilon = 0.3, \gamma = 1.5, p_m = 0.5$

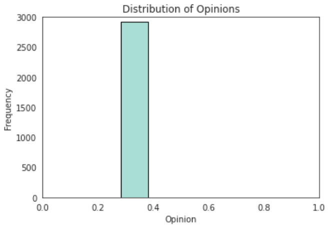

(i) $\varepsilon = 0.4, \gamma = 0.0, p_m = 0.5$

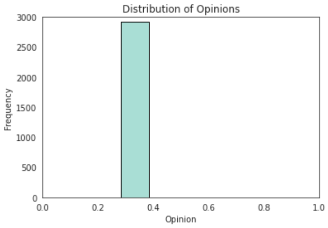

(j) $\varepsilon = 0.4, \gamma = 0.5, p_m = 0.5$

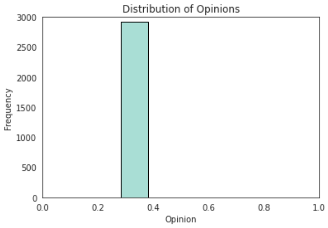

(k) $\varepsilon = 0.4, \gamma = 1.0, p_m = 0.5$

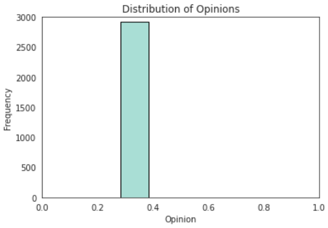

(l) $\varepsilon = 0.4, \gamma = 1.5, p_m = 0.5$

Supplementary Figure 52. Final opinion distribution in the Algorithmic Bias Model with Mass Media ($p_m = 0.5$) with a single mass media aligned with the average opinion of the pro faction. From top to bottom: $\varepsilon = 0.2$, $\varepsilon = 0.3$ and $\varepsilon = 0.4$. From left to right: $\gamma = 0.0$, $\gamma = 0.5$, $\gamma = 1.0$, $\gamma = 1.5$.

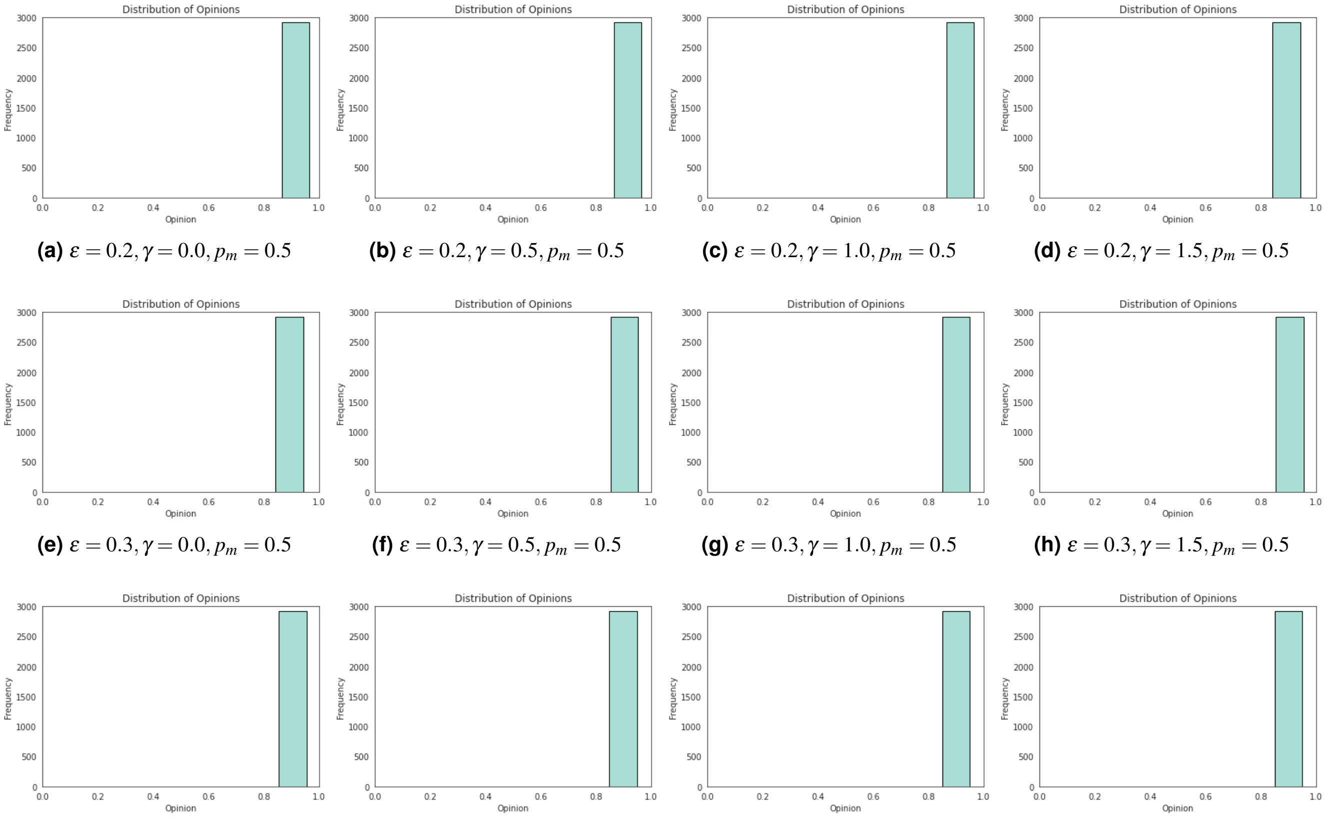

(a) $\varepsilon = 0.2, \gamma = 0.0, p_m = 0.5$ **(b)** $\varepsilon = 0.2, \gamma = 0.5, p_m = 0.5$ **(c)** $\varepsilon = 0.2, \gamma = 1.0, p_m = 0.5$ **(d)** $\varepsilon = 0.2, \gamma = 1.5, p_m = 0.5$

(e) $\varepsilon = 0.3, \gamma = 0.0, p_m = 0.5$ **(f)** $\varepsilon = 0.3, \gamma = 0.5, p_m = 0.5$ **(g)** $\varepsilon = 0.3, \gamma = 1.0, p_m = 0.5$ **(h)** $\varepsilon = 0.3, \gamma = 1.5, p_m = 0.5$

(i) $\varepsilon = 0.4, \gamma = 0.0, p_m = 0.5$ **(j)** $\varepsilon = 0.4, \gamma = 0.5, p_m = 0.5$ **(k)** $\varepsilon = 0.4, \gamma = 1.0, p_m = 0.5$ **(l)** $\varepsilon = 0.4, \gamma = 1.5, p_m = 0.5$

Supplementary Figure 53. Final opinion distribution in the Algorithmic Bias Model with Mass Media ($p_m = 0.5$) with a single mass media aligned with the average opinion of the against faction. From top to bottom: $\varepsilon = 0.2$, $\varepsilon = 0.3$ and $\varepsilon = 0.4$. From left to right: $\gamma = 0.0$, $\gamma = 0.5$, $\gamma = 1.0$, $\gamma = 1.5$.

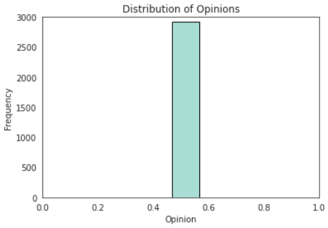

(a) $\varepsilon = 0.2, \gamma = 0.0, p_m = 0.5$

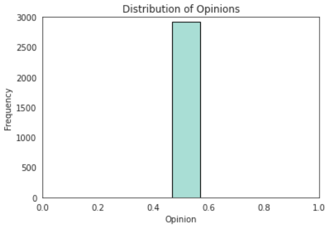

(b) $\varepsilon = 0.2, \gamma = 0.5, p_m = 0.5$

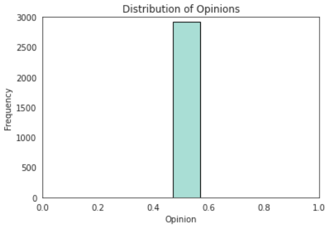

(c) $\varepsilon = 0.2, \gamma = 1.0, p_m = 0.5$

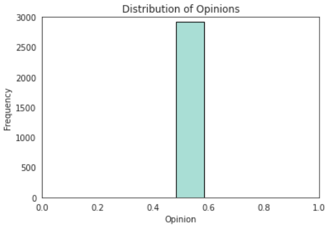

(d) $\varepsilon = 0.2, \gamma = 1.5, p_m = 0.5$

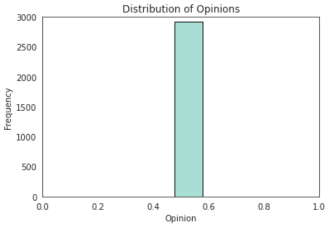

(e) $\varepsilon = 0.3, \gamma = 0.0, p_m = 0.5$

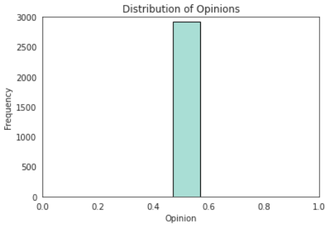

(f) $\varepsilon = 0.3, \gamma = 0.5, p_m = 0.5$

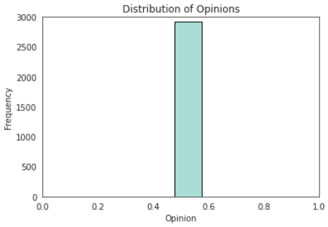

(g) $\varepsilon = 0.3, \gamma = 1.0, p_m = 0.5$

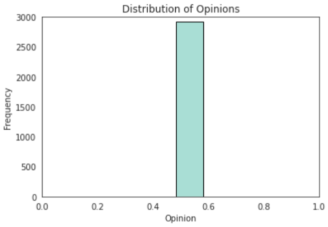

(h) $\varepsilon = 0.3, \gamma = 1.5, p_m = 0.5$

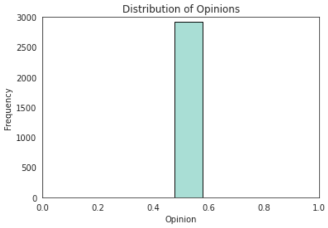

(i) $\varepsilon = 0.4, \gamma = 0.0, p_m = 0.5$

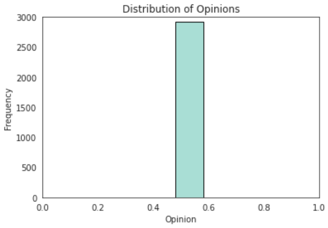

(j) $\varepsilon = 0.4, \gamma = 0.5, p_m = 0.5$

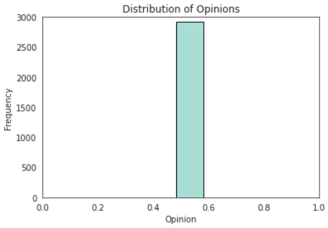

(k) $\varepsilon = 0.4, \gamma = 1.0, p_m = 0.5$

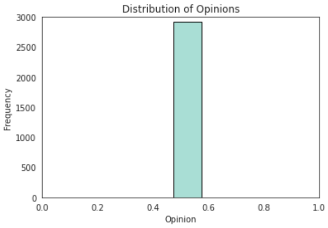

(l) $\varepsilon = 0.4, \gamma = 1.5, p_m = 0.5$

Supplementary Figure 54. Final opinion distribution in the Algorithmic Bias Model with Mass Media ($p_m = 0.5$) with a single mass media aligned with the average opinion of the neutral faction. From top to bottom: $\varepsilon = 0.2$, $\varepsilon = 0.3$ and $\varepsilon = 0.4$. From left to right: $\gamma = 0.0$, $\gamma = 0.5$, $\gamma = 1.0$, $\gamma = 1.5$.

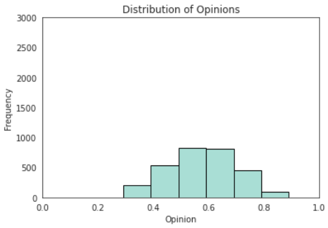

(a) $\varepsilon = 0.2, \gamma = 0.0, p_m = 0.5$

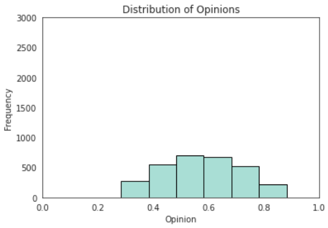

(b) $\varepsilon = 0.2, \gamma = 0.5, p_m = 0.5$

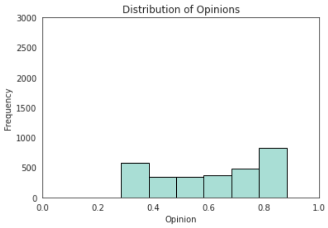

(c) $\varepsilon = 0.2, \gamma = 1.0, p_m = 0.5$

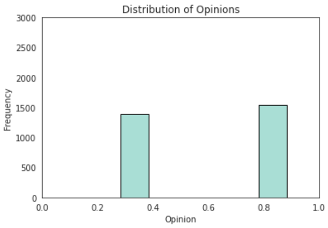

(d) $\varepsilon = 0.2, \gamma = 1.5, p_m = 0.5$

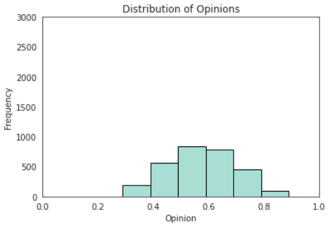

(e) $\varepsilon = 0.3, \gamma = 0.0, p_m = 0.5$

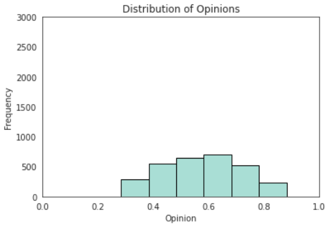

(f) $\varepsilon = 0.3, \gamma = 0.5, p_m = 0.5$

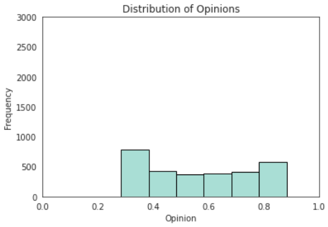

(g) $\varepsilon = 0.3, \gamma = 1.0, p_m = 0.5$

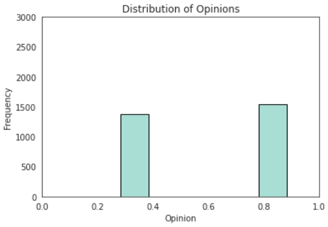

(h) $\varepsilon = 0.3, \gamma = 1.5, p_m = 0.5$

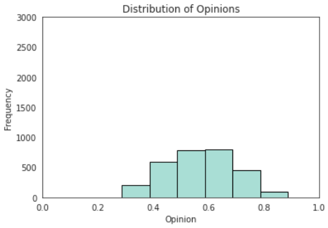

(i) $\varepsilon = 0.4, \gamma = 0.0, p_m = 0.5$

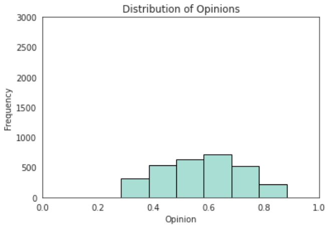

(j) $\varepsilon = 0.4, \gamma = 0.5, p_m = 0.5$

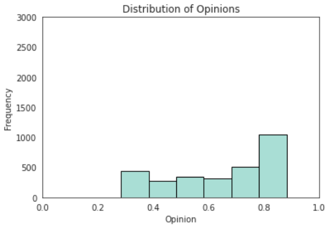

(k) $\varepsilon = 0.4, \gamma = 1.0, p_m = 0.5$

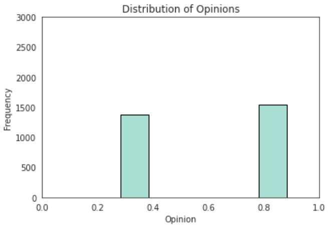

(l) $\varepsilon = 0.4, \gamma = 1.5, p_m = 0.5$

Supplementary Figure 55. Final opinion distribution in the Algorithmic Bias Model with Mass Media ($p_m = 0.5$) with two polarized mass media aligned with the pro and against average opinion, respectively. From top to bottom: $\varepsilon = 0.2$, $\varepsilon = 0.3$ and $\varepsilon = 0.4$. From left to right: $\gamma = 0.0$, $\gamma = 0.5$, $\gamma = 1.0$, $\gamma = 1.5$.

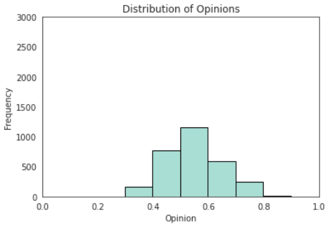

(a) $\varepsilon = 0.2, \gamma = 0.0, p_m = 0.5$

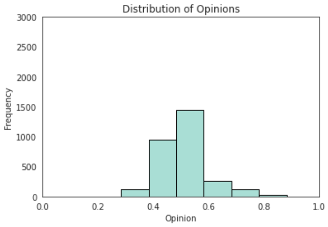

(b) $\varepsilon = 0.2, \gamma = 0.5, p_m = 0.5$

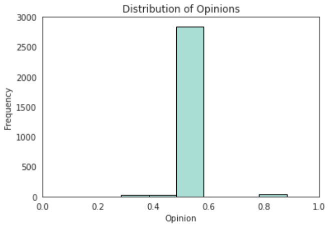

(c) $\varepsilon = 0.2, \gamma = 1.0, p_m = 0.5$

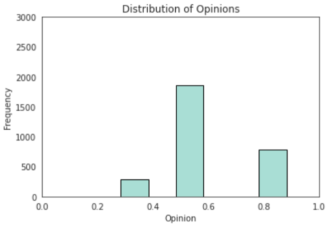

(d) $\varepsilon = 0.2, \gamma = 1.5, p_m = 0.5$

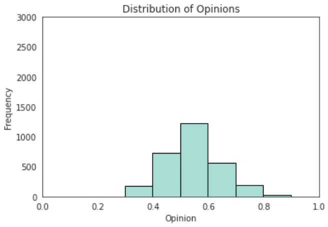

(e) $\varepsilon = 0.3, \gamma = 0.0, p_m = 0.5$

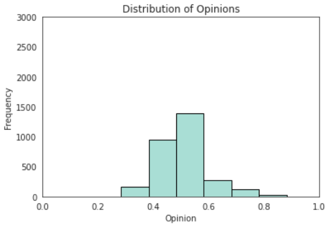

(f) $\varepsilon = 0.3, \gamma = 0.5, p_m = 0.5$

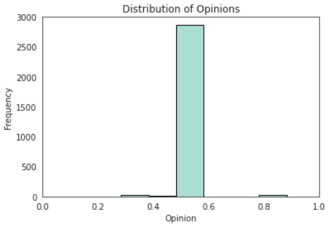

(g) $\varepsilon = 0.3, \gamma = 1.0, p_m = 0.5$

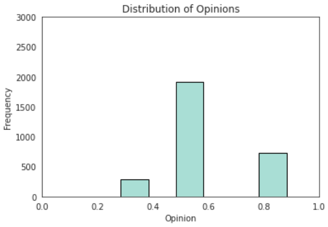

(h) $\varepsilon = 0.3, \gamma = 1.5, p_m = 0.5$

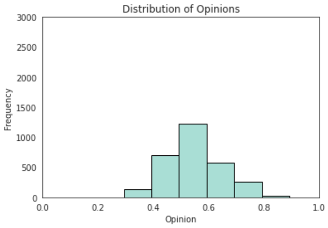

(i) $\varepsilon = 0.4, \gamma = 0.0, p_m = 0.5$

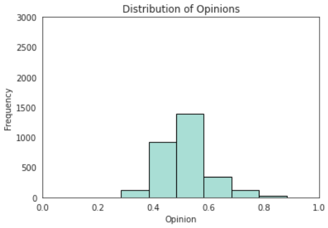

(j) $\varepsilon = 0.4, \gamma = 0.5, p_m = 0.5$

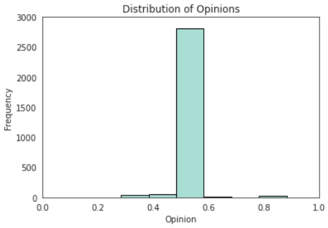

(k) $\varepsilon = 0.4, \gamma = 1.0, p_m = 0.5$

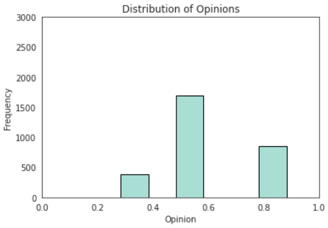

(l) $\varepsilon = 0.4, \gamma = 1.5, p_m = 0.5$

Supplementary Figure 56. Final opinion distribution in the Algorithmic Bias Model with Mass Media ($p_m = 0.5$) with two polarized mass media aligned with the pro and against average opinion, and a mass media aligned with the neutral average opinion. From top to bottom: $\varepsilon = 0.2$, $\varepsilon = 0.3$ and $\varepsilon = 0.4$. From left to right: $\gamma = 0.0$, $\gamma = 0.5$, $\gamma = 1.0$, $\gamma = 1.5$.

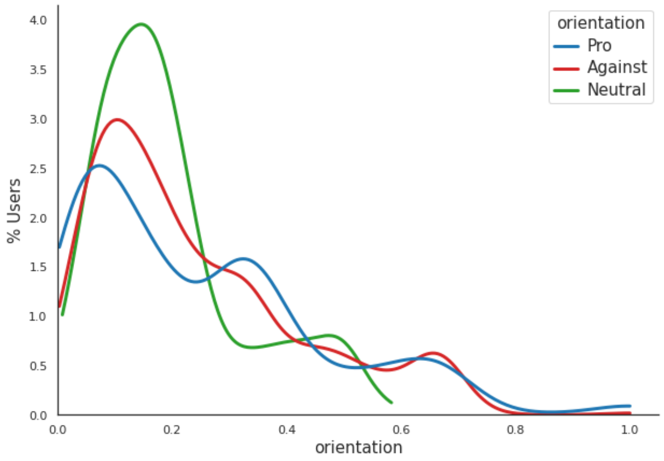

Supplementary Figure 57. Estimated open-mindedness ε distributions for each leaning. Each subpopulation presents a positively skewed distribution, with the highest proportion of users having $\varepsilon \leq 0.3$, which in the Deffuant-Weisbuch model[4] is considered a sufficient condition to have opinion polarization in the long term. Neutrals (green) present values ranging between 0 and 0.6, while Pro (blue) and Against (red) users present values between 0 and 1, but with a peak on lower levels of ε with respect to Neutrals.

Algorithm 1 Confidence bound estimation algorithm.

G_t = Weighted undirected interaction network at time t;
V_t = set of nodes at time t;
E_t = set of weighted edges at time t;
$x_u(t)$ = opinion of agent u at time t;
$d_{u,v} = |x_u(t) - x_v(t)|$ = opinion distance between $u, v \in V$ at time t;
$\widehat{CB}$ = estimated confidence bounds.

if $u \in V_{t+1}$ **then**
  Procedure to estimate $\widehat{x_u}(t+1)$ and $\widehat{CB}_u$
  $N_{u,t} = \{v | (u,v) \in E_t\}$; $|N_{u,t}| = n$
  $X_{N_{u,t}}[1...n]$ = Array of opinions of nodes $v \in N_{u,t}$
  if $N_{u,t} \neq \emptyset$ **then**
    Sort $X_{N_{u,t}}[1...n]$ *by* $d_{u,v}$ in ascending order.
    $\widehat{X_u}(t+1)[0...n]$ array of estimated opinions
    $\widehat{X_u}(t+1)[0] = x_u(t)$
    $E = [0...n]$ array of estimation errors
    $E[0] = 1.0$
    for i=1; i=n; i++ **do**
      $x_v = X_{N_{u,t}}[i]$
      $\widehat{X_u}(t+1)[i] = \frac{\hat{X}_u(t+1)[i-1] + x_v}{2}$
      $E[i] = |\widehat{X_u}(t+1)[i] - x_u(t+1)|$
      $min_e = E[n]$
      for i=n; i=0; i– **do**
        $e = E[i]$
        if $e \leq min_e$ **then**
          $min_e = e$
          $j = i$
        end if
      end for
    end for
    $\widehat{x_u}(t+1) = \hat{X}_u(t+1)[j]$
    $\widehat{CB} = |x_u(t) - X_{N_{v,t}}[j]|$
  end if
end if

Figure 58(a) with $\gamma = 0.0$, the opinion evolution process exacerbates the existing polarization from the initial condition G_0. However, for $\gamma \geq 0.5$, the neighbors of users aligned with the opposing faction exhibit a more diverse range of opinions, thus reducing the polarization within this subpopulation and the overall network. This effect intensifies as γ increases. Similar outcomes are observed when users can interact with a single mass media source promoting an opinion in favor of "taking the knee" ($x_m = 0.28$, the average opinion of the pro faction). Notably, the presence of a "stubborn agent" with an opinion of 0.28 facilitates the dissolution of the echo chamber among users opposing "taking the knee", resulting in outcomes similar to the real situation for $\gamma \geq 1.0$. The correlation between nodes' opinions and the average opinions of their nearest neighbors is 0.87 in the real setting, 0.93 in Supplementary Figure 58(d), and 0.90 in Supplementary Figure 59(d). These values are quite similar, indicating that algorithmic bias primarily drives this depolarization, but such a process is further aided by promoting an opinion aligned with the pro faction. In the case of a mass media source promoting the opinion of the opposing faction ($x_m = 0.87$), the depolarization of the pro echo chamber is less pronounced, and two distinct echo chambers persist in the network (see Supplementary Figure 60). The presence of a neutral media, as observed in Supplementary Figure 61, leads to the most drastic change in the distribution of opinions across the network in the final state. In the absence of bias (Supplementary Figure 61(a)), the correlation between nodes' opinions and their neighbors' is lower than in previous scenarios, and users form a single community primarily aligned around moderate, central positions. However, as the bias γ increases, homophilic behavior emerges in the network, although most users remain in the moderate community. As depicted in Supplementary Figure 62, polarization is higher than the starting conditions in all cases, yet the against echo chamber becomes less prominent as the bias grows. Similar outcomes are observed in the case of three media sources (see Supplementary Figure 63), where the presence of a neutral media alone is insufficient to disrupt the two echo chambers.

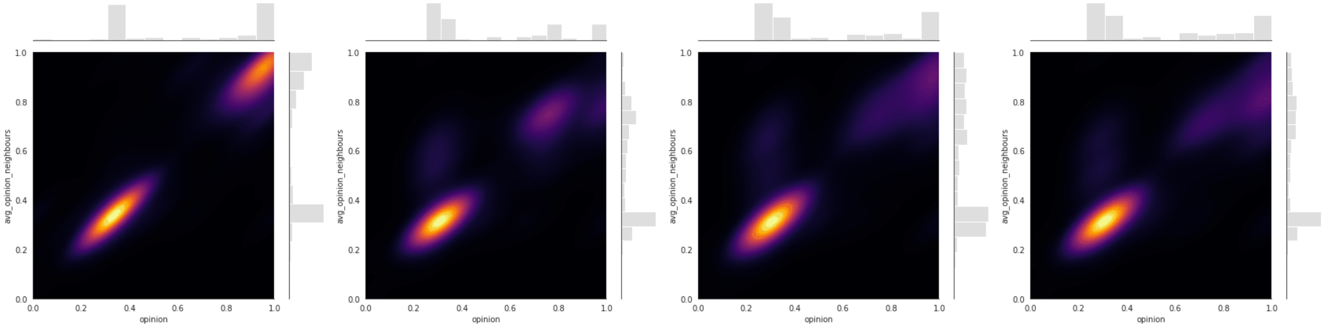

(a) $p_m = 0.0, \gamma = 0.0$ **(b)** $p_m = 0.0, \gamma = 0.5$ **(c)** $p_m = 0.0, \gamma = 1.0$ **(d)** $p_m = 0.0, \gamma = 1.5$

Supplementary Figure 58. Joint distribution of the opinion of users and average leaning of their neighborhood in absence of a mass media source $p_m = 0.0$. Joint distribution of the opinion of users and average leaning of their neighborhood for $p_m = 0.0$ and increasing values of γ (from left to right). For $\gamma = 0.0$ the level of polarization in the network increases with respect to the starting condition G_0. Instead, a positive γ reduces the level of polarization in the network.

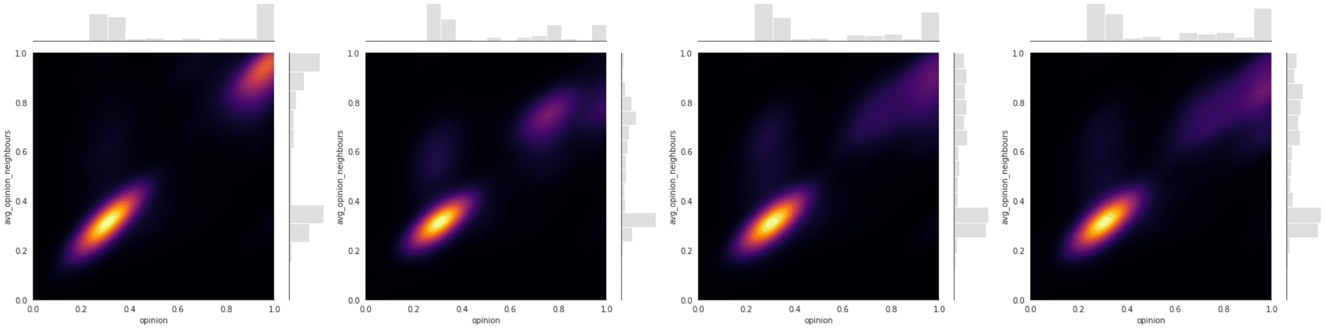

(a) $p_m = 0.0, \gamma = 0.0$ **(b)** $p_m = 0.0, \gamma = 0.5$ **(c)** $p_m = 0.0, \gamma = 1.0$ **(d)** $p_m = 0.0, \gamma = 1.5$

Supplementary Figure 59. Joint distribution of the opinion of users and average leaning of their neighborhood with a random probability to interact with a mass media ($p_m = 0.5$) aligned with the average opinion of the pro faction. Joint distribution of the opinion of users and average leaning of their neighborhood with a random probability to interact with a mass media ($p_m = 0.5$) aligned with the average opinion of the pro faction and increasing values of γ (from left to right).

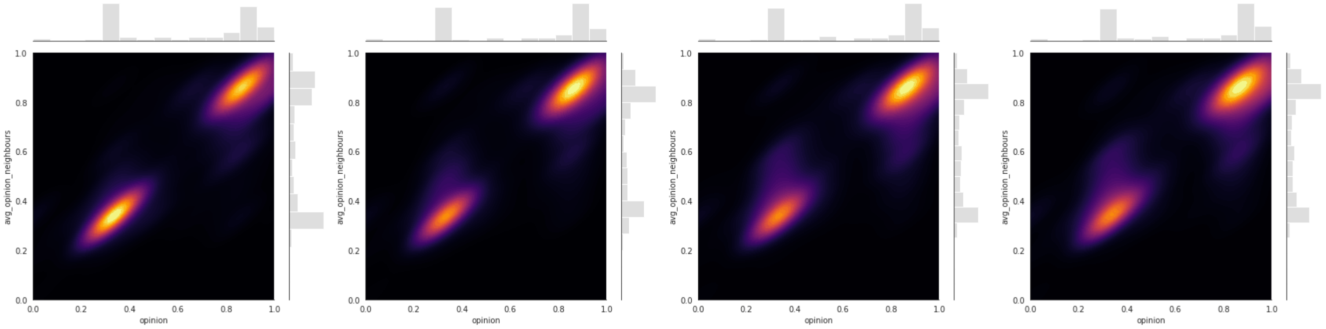

(a) $p_m = 0.0, \gamma = 0.0$ **(b)** $p_m = 0.0, \gamma = 0.5$ **(c)** $p_m = 0.0, \gamma = 1.0$ **(d)** $p_m = 0.0, \gamma = 1.5$

Supplementary Figure 60. Joint distribution of the opinion of users and average leaning of their neighborhood with a random probability to interact with a mass media ($p_m = 0.5$) aligned with the average opinion of the against faction. Joint distribution of the opinion of users and average leaning of their neighborhood with a random probability to interact with a mass media ($p_m = 0.5$) aligned with the average opinion of the against faction and increasing values of γ (from left to right).

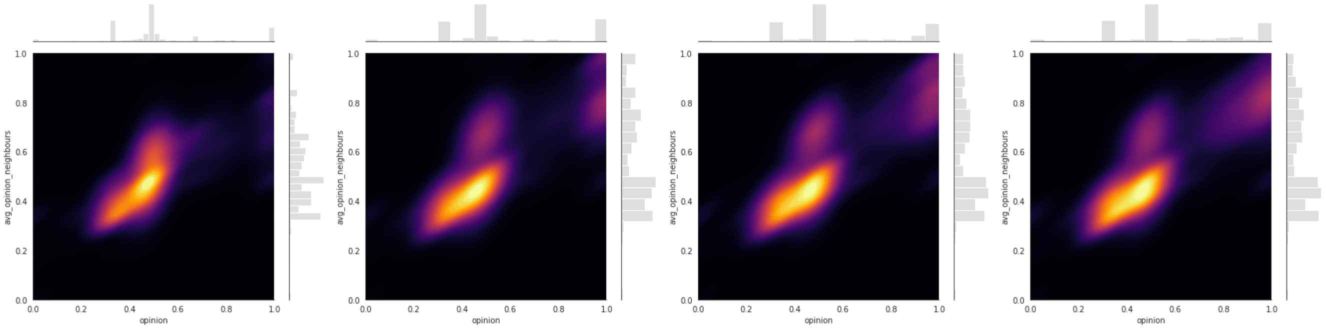

(a) $p_m = 0.0, \gamma = 0.0$ **(b)** $p_m = 0.0, \gamma = 0.5$ **(c)** $p_m = 0.0, \gamma = 1.0$ **(d)** $p_m = 0.0, \gamma = 1.5$

Supplementary Figure 61. Joint distribution of the opinion of users and average leaning of their neighborhood with a random probability to interact with a mass media ($p_m = 0.5$) aligned with the average opinion of the neutral faction. Joint distribution of the opinion of users and average leaning of their neighborhood with a random probability to interact with a mass media ($p_m = 0.5$) aligned with the average opinion of the neutral faction and increasing values of γ (from left to right).

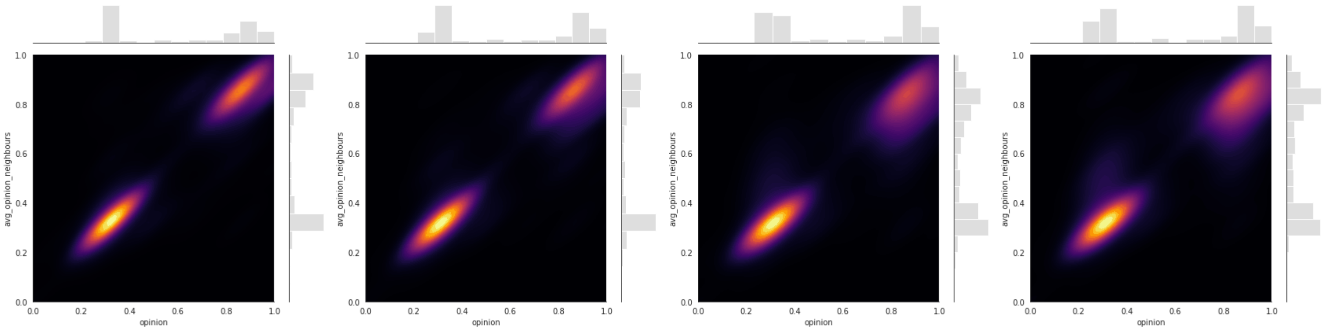

(a) $p_m = 0.0, \gamma = 0.0$ **(b)** $p_m = 0.0, \gamma = 0.5$ **(c)** $p_m = 0.0, \gamma = 1.0$ **(d)** $p_m = 0.0, \gamma = 1.5$

Supplementary Figure 62. Joint distribution of the opinion of users and average leaning of their neighborhood with a random probability to interact with a mass media ($p_m = 0.5$) and two polarized mass media. Joint distribution of the opinion of users and average leaning of their neighborhood with a random probability to interact with a mass media ($p_m = 0.5$) aligned with the average opinion of the pro faction and the average leaning of the against faction and increasing values of γ (from left to right).

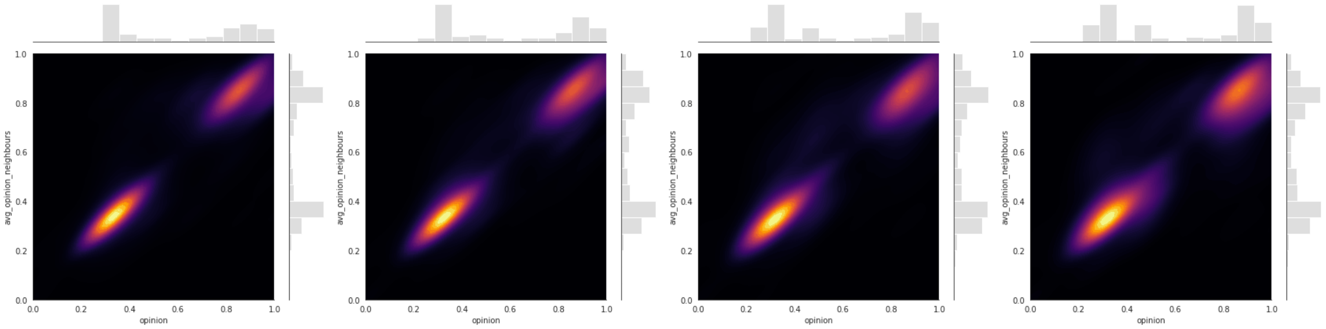

(a) $p_m = 0.0, \gamma = 0.0$ **(b)** $p_m = 0.0, \gamma = 0.5$ **(c)** $p_m = 0.0, \gamma = 1.0$ **(d)** $p_m = 0.0, \gamma = 1.5$

Supplementary Figure 63. Joint distribution of the opinion of users and average leaning of their neighborhood with a random probability to interact with a mass media ($p_m = 0.5$) and 3 mass media. Joint distribution of the opinion of users and average leaning of their neighborhood with a random probability to interact with a mass media ($p_m = 0.5$). The opinions of the three mass media are respectively: the average opinion of the pro faction, the average opinion of the neutral faction and the average opinion of the against faction.

References

1. Rossetti, G., Citraro, S. & Milli, L. Conformity: a path-aware homophily measure for node-attributed networks. *IEEE Intell. Syst.* **36**, 25–34 (2021).

2. Buongiovanni, C. *et al.* Will you take the knee? italian twitter echo chambers' genesis during euro 2020. In *Complex Networks and Their Applications XI: Proceedings of The Eleventh International Conference on Complex Networks and Their Applications: COMPLEX NETWORKS 2022—Volume 1*, 29–40 (Springer, 2023).

3. Pansanella, V., Morini, V., Squartini, T. & Rossetti, G. Change my mind: Data driven estimate of open-mindedness from political discussions. In *Complex Networks and Their Applications XI: Proceedings of The Eleventh International Conference on Complex Networks and Their Applications: COMPLEX NETWORKS 2022—Volume 1*, 86–97 (Springer, 2023).

4. Deffuant, G. & Weisbuch, G. Mixing beliefs among interacting agents. *Adv. Complex Syst.* **3**, 87–98, DOI: 10.1142/S0219525900000078 (2000).
